# The Evolving Faces of the SARS-CoV-2 Genome

**DOI:** 10.3390/v13091764

**Published:** 2021-09-03

**Authors:** Maria Schmidt, Mamoona Arshad, Stephan H. Bernhart, Siras Hakobyan, Arsen Arakelyan, Henry Loeffler-Wirth, Hans Binder

**Affiliations:** 1 IZBI, Interdisciplinary Centre for Bioinformatics, Universität Leipzig, Härtelstr. 16–18, 04107 Leipzig, Germany; ma68lyke@studserv.uni-leipzig.de (M.A.); bernhart@izbi.uni-leipzig.de (S.H.B.); wirth@rz.uni-leipzig.de (H.L.-W.); 2 Armenian Bioinformatics Institute (ABI), 7 Hasratyan Str., Yerevan 0014, Armenia; siras.hakobyan@abi.am (S.H.); arsen.arakelyan@abi.am (A.A.); 3 Research Group of Bioinformatics, Institute of Molecular Biology of the National Academy of Sciences of the Republic of Armenia, 7 Hasratyan Str., Yerevan 0014, Armenia

**Keywords:** COVID-19, virus sequencing, single nucleotide variants, SARS-CoV-2 lineages genomic surveillance, self-organizing maps portrayal, machine learning

## Abstract

Surveillance of the evolving SARS-CoV-2 genome combined with epidemiological monitoring and emerging vaccination became paramount tasks to control the pandemic which is rapidly changing in time and space. Genomic surveillance must combine generation and sharing sequence data with appropriate bioinformatics monitoring and analysis methods. We applied molecular portrayal using self-organizing maps machine learning (SOM portrayal) to characterize the diversity of the virus genomes, their mutual relatedness and development since the beginning of the pandemic. The genetic landscape obtained visualizes the relevant mutations in a lineage-specific fashion and provides developmental paths in genetic state space from early lineages towards the variants of concern alpha, beta, gamma and delta. The different genes of the virus have specific footprints in the landscape reflecting their biological impact. SOM portrayal provides a novel option for ‘bioinformatics surveillance’ of the pandemic, with strong odds regarding visualization, intuitive perception and ‘personalization’ of the mutational patterns of the virus genomes.

## 1. Introduction

As of July 2021, severe acute respiratory syndrome coronavirus 2 (SARS-CoV-2), the causative agent of COVID-19 pandemic, accounted for more than 190 million infections and more than four million deaths worldwide. Day by day nearly half a million new cases were diagnosed and more than 8000 people die, a rate which is roughly as high as during the first wave of the pandemic in spring 2020. During 2020, the first year of the pandemic spread, research efforts focused on three major issues: firstly sequence analysis of the early root-variants of the virus to discover its origin, develop PCR-tests and to design vaccines; secondly, monitoring epidemic numbers (daily incidence, deaths etc.) of the pandemic to identify factors which reduce its spread and local outbreak events in the context of non-pharmaceutical interventions (NPI, e.g., mask wearing, social distancing, lock-down measures) including prognostic modelling and epidemiological surveillance; and thirdly, understanding the clinics and the molecular mechanisms of the disease to improve treatment and medical interventions from short (-ICU) to long (-COVID) time scales. Systematic sequencing was not among the top research and surveillance issues on global scale, presumably because the emergence of SARS-CoV-2 in late 2019 was followed by a period of apparent evolutionary stasis of the virus genome lasting nearly one year [1]. Compared to other viruses such as HIV, SARS-CoV-2 was found to change much more slowly during its spread.

‘The coronavirus is mutating—does it matter?’ *Nature* was asking in September 2020 [2]. It seemed that slightly varying SARS-CoV-2 strains did not have major impact on the course of the pandemic, ‘…but they might in future’, it had been concluded [2]. The ’future’ just began immediately after this statement in autumn 2020: SARS-COV-2 evolution emerged into ‘variants of concern’ (VOCs), which developed mutations that impact virus characteristics in terms of increased transmissibility and changed antigenicity [3,4,5,6]. VOCs were nick-named as ‘British’, ‘Brazilian’, ‘South-African’ and ‘Indian’ according to the region of first appearance or first documentation. They replaced previous variants and gave rise to oscillating waves of incidence around the world until now.

Sequencing and the use of pathogen genomes on large scale became a ‘first-need’ task to track the spread of the virus, to study local outbreaks, to track transmission, to flag key mutations and, last but not least, to support political decision-making [7]. Moreover, the prospect of reduced vaccine potency from fast-spreading SARS-CoV-2 variants now has spurred a global rush to increase genomic surveillance. Virus sequences are now being generated and shared at an unprecedented rate and opened a new age of virus genomic studies. More than two million SARS-CoV2 sequences are available in total and thousands of new sequences coming in each day via GISAID (the Global Initiative on Sharing All Influenza Data) to permit a near real-time surveillance of the pandemic [8,9] for a better understanding of the dynamics of viral spread and evolution [10]. Sequencing provided a detailed picture of the changing virus, presumably the best documented virus evolutionary process so far. Phylogenies are updated and published on a daily basis on nextstrain.org, which is crucial for quickly identifying and tracking emergent strains.

Bioinformatics tools and opportunities are buckling under the flood of coronavirus genome sequences and under the pressure of task they are needed for; e.g., to help control the pandemic [11]. It is also difficult to infer a reliable phylogeny due to the large number of sequences in conjunction with the relatively low number of mutations in a relatively small genome. Methods to disentangle the evolution and spread of COVID-19 should be considered and interpreted with caution [11]. *Nature* now asked ‘How to fix the bioinformatics bottleneck?’ and suggested that researchers must move beyond the limitations of existing tools [11].

We here aim at glimpsing at SARS-CoV-2 genome diversity in time and space using ‘Self Organizing Map (SOM) portrayal’, a machine-learning based method, which has been proven in numerous applications in omics-bioinformatics, mostly transcriptomic studies of genomic regulation in health and disease [12,13,14]. The method offers two major opportunities: firstly, it ‘portrays’ high-dimensional data by providing personal images visualizing, e.g., the faces of personalized tumor transcriptomes. Portraits then can be inspected and compared without deeper bioinformatics expertise. Secondly, it reduces dimensionality in a harmonized way, meaning that all relevant aspects of information are maintained and remain hidden but available for detailed downstream analysis [15]. We recently adjusted the method to infer developmental trajectories in sample and gene state space to describe tissue differentiation [16]. Application of SOM portrayal to large worldwide collections of genomic data, namely of humans [17] and vine accessions [18], deciphered genomic footprints of human migration and of dissemination vine cultivation over geographic regions during the last thousands of years. In continuation of this concept, we aimed at characterizing footprints of the spread and evolution in the SARS-CoV-2 genome since its emergence in late 2019 by means of SOM portrayal. After introducing the method, we delineate the distribution of virus variants in space and time, chart the genomic landscapes to draft trajectories of virus evolution. We provide an interactive tool for browsing the SOM portraits of the virus variants, and we extend the method to add new genomes to the existing landscape.

## 2. Materials and Methods

### 2.1. SARS-CoV-2 Genome Data and Preprocessing

**Figure 1:**
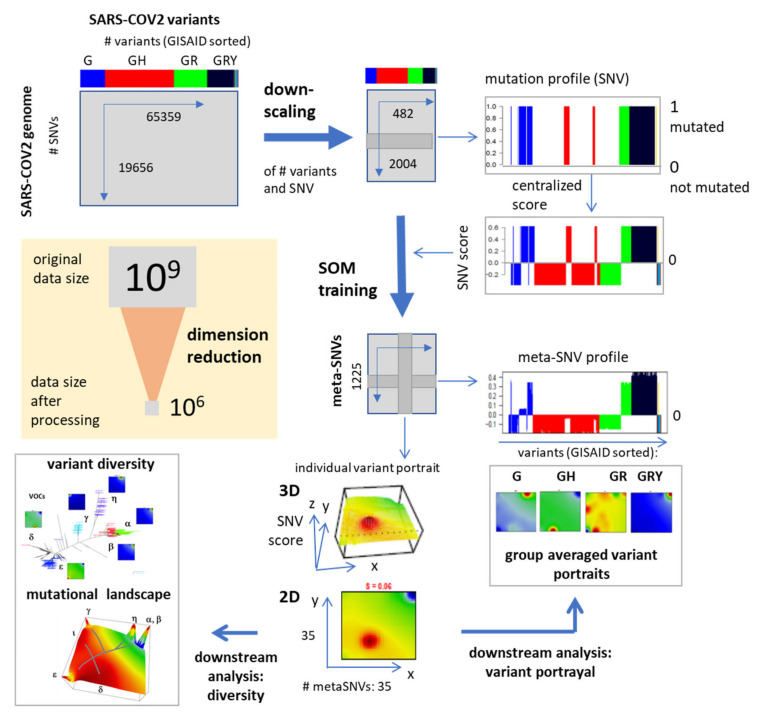
SOM pipeline for portraying the SARS-CoV-2 genomes. Data processing includes downscaling of the number of available variants and transformation of the relevant genome size by SOM training. It clusters mutation profiles across variants and visualizes them as ‘portraits’, i.e., three dimensional images of the mutation score as a function of the relevant mutations. We use projection of the SNV-score into the x-y plane spanned the meta-SNVs in the SOM grid in the following. Data size reduces by a factor of 10^3^ after processing.

SARS-CoV-2 genome data were taken from NCBI virus database on 14 April 2021. After removing short sequence snippets, the original data set consisted of 65,359 SARS-CoV-2 genomes assigned to different labeling schemes (Table A1) [6,7,8,9,10,17], namely those (i) of the World Health Organization (WHO) using Greek letters for Variants of Concern (VOCs) and Variants of Interest (VOIs); (ii) clades proposed by the GISAID (Global Initiative on Sharing All Influenza Data) [9], (iii) lineages suggested by the PANGOLIN (Phylogenetic Assignment of Named Global Outbreak LINeages) tool [19], (iv) the years-and-letter nomenclature code introduced by Nextstrain, all combined with information about date and geographic location, when and where the respective samples were collected. Sequences were downloaded as FASTA files and mapped to the reference genome (NC_045512.2m WIV04, [20]) using BLAST to obtain the mutated positions in terms of SNVs (Single Nucleotide Variants) for each variant of the genome. Overall 19,656 SNVs out of the full genome length of 30,402 nts were found mutated at minimum once in the whole data set. For efficient computation, we downscaled the number of SARS-CoV-2 variants by selecting around 10–80 genomes from the pool of each of the VOC/VOI clades and by selecting the same number of variants randomly from the remaining not-VOC/VOI clades, which results in 483 genomes in the final data set overall mutated at 2004 SNV positions. The final data matrix for subsequent SOM training thus consists of 2004 SNVs x 483 variants of the virus (Figure 1). Genomic data for analysis extension were downloaded from GISAID’s EpiCoV Database (https://www.gisaid.org/, accessed on 16 June 2021) and processed as mentioned above.

### 2.2. Mutation Coding, SOM Training and Genome Portrayal

Next, we coded each sequence position in each variant by its mutation status using a binary code with the value ‘0′ for not-mutated ones and ‘1′ for mutated ones, which provides a SNV- profile for each genome position across all variants of the virus (Figure 1, top right). Then, each SNV-profile was centralized by subtracting the respective mean SNV-score averaged over all variants in order to highlight the variability of individual strains and prepare data for efficient clustering. In the next step, the centralized SNV-profiles were used to train a Self-Organizing Map (SOM). SOM training translates the original data matrix into a data matrix of reduced dimensionality of K = 35 × 35 = 1225, so-called meta-SNV profiles. Hereby, the term ‘profile’ denotes the vector of SNV score values across the virus variants. The SOM training algorithm distributes the SNV-profiles over the K meta-SNPs by minimizing the Euclidean distance as cost function. Each meta-SNV profile of the trained SOM can be interpreted as the mean profile averaged over all SNV profiles of the respective meta-SNV cluster. The meta-SNV values of each variant are visualized by arranging them into a quadratic K = 35 × 35 grid and using a red to blue color- code for maximum to minimum SNV score-values in each of the images. This way they ‘portray’ the genetic landscape of each virus genome studied where red areas refer to predominantly mutated and blue areas to predominantly not mutated sequence positions. As an alternative, we applied ‘coastline’ images which use a logarithmic scale highlighting areas of mainly mutated and not mutated meta-SNVs in red and blue colors, respectively [15].

**Figure A1:**
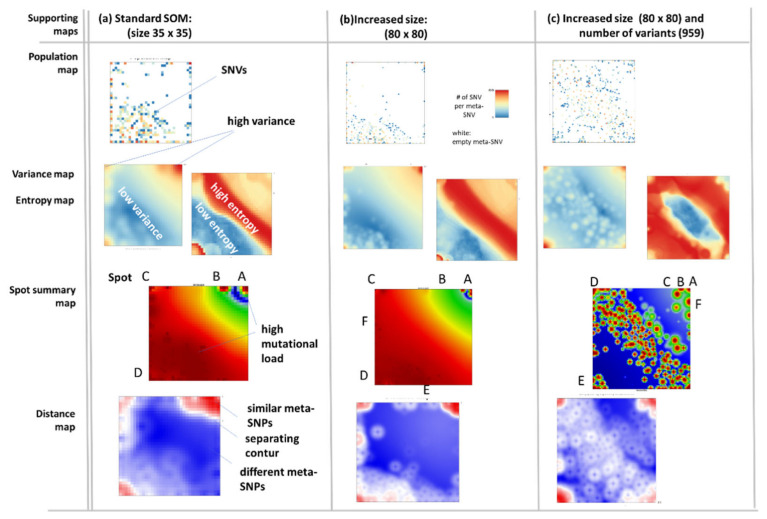
Comparison of SOM- and sample-size using supporting maps. Increasing size of the SOM retains its internal structure. In conclusion, SOM of standard size (35 × 35) well reproduces the landscape of the data. Supporting maps were described previously [15]. In short: the population map shows the number of SNVs per pixel/meta-SNV. Increasing size increases the amount of empty meta-SNVs (white) but virtually leaves the occupied meta-SNV unchanged. The variance and entropy maps color code the variance and entropy of the meta-SNV. Largest variance (red) is found in the highly populated ‘spot’ areas. Entropy is maximum in the areas of intermediate variance. The spot summary map provides an overview about the spots of high mutational load seen in the individual portraits of variants. The distance map color codes the intrinsic distance metric of the SOM. White ‘halos’ surround clusters of SNV-modes collecting meta-SNV of similar profiles. A nearly doubled number of variants (949) mostly of non-VOC types shifts the SNV-floor towards the central part of the SOM-image. In conclusion, the particular size of the SOM and number of variants considered distorts the SNV-landscape obtained but reproduces its basic features. Their interpretation relates consequently to the particular parametrization of the SOM.

We used the SOM analysis pipeline as implemented in the publicly available R package oposSOM [21]. Variants were labelled according to their GISAID, Pangolin or VOC/VOI (variant of concern/variant of interest) assignment, and by using our pattern type (PAT, see below) classification (Table A1, Appendix A). Mean class portraits were obtained by averaging the meta-SNV values of the respective individual variant portraits over the respective class. The effect of parameter variation in terms of SOM and sample size optimization is addressed in Figure A1 (Appendix B).

### 2.3. Spot Detection, Pattern Types (PATs) and Diversity Analysis

The self-organization during the SOM training distributes the SNV profiles over the map such that similar profiles are mapped to neighboring positions whereas dissimilar ones are located more distantly. This leads to spot-like regions (red areas in the portraits) referring to correlated SNV-profiles showing high SNV scores in the respective variant. We used previously developed segmentation algorithms [15,22] to extract the so-called spot-clusters from these regions. Each of the spot-clusters includes typically a few dozen to hundreds of SNVs. One portrait can contain more than one spot. Variants can be subsequently classified by mutual similarity of their spot patterns into pattern types (PATs, see Results section). Sample diversity analysis was performed based on the variant portraits using phylogenetic similarity tree, independent component analysis and sample SOM plots as implemented in oposSOM [21,23] and also using URD-pseudotime analysis (program ‘URD’ [24]).

### 2.4. Extension SOM (xSOM)

The extension SOM method (xSOM) [25] aims at adding new, secondary data (e.g., newly sequenced SARS- COV2 variants) to an already existing SOM in order to maintain original distribution of SNV in the map and therefore also the spot-clusters defined for the sake of comparison. For this, the original SOM algorithm was adapted to realize standard meta-SNV training for the variants already contained in the original SOM training and a passive, ‘piggyback’ training of the meta-SNVs for the extension data. This approach provides unchanged meta-SNV scores for the original variant data and appropriately trained meta-SNV scores for the additional data. We used xSOM to portray the SARS-CoV-2 genomes collected from 36 COVID-19 patients in Armenia (24 in January, 12 in March) [26] and to extend the world data with selected variants such as the ‘Indian’ variant delta.

### 2.5. SARS-CoV-2 oposSOM Browser and Epidemiological Numbers

Further details of the analysis of the SARS-CoV-2 genome addressed in this publication can be interactively discovered using the oposSOM browser [27] available online via the IZBI web page (https://www.izbi.uni-leipzig.de/opossom-browser/ and https://apps.health-atlas.de/opossom-browser/?dataset=12). The browser enables selection and visualization of SNVs in the genome landscape, and assessment of similarity relations between the variants and lineages together with their individual SOM portraits (see also Appendix B, Figure A11 and Figure A12).

Plots of the numbers of cases (incidence) and number of deaths (death toll) as a function of time from early 2020 to summer 2021 were generated using the COVID-19 viewer (https://www.izbi.uni-leipzig.de/current-projects/covid19-viewer/, accessed on 17 July 2021) [28]. Composition of variants were downloaded as genomic metadata from GISAID’s EpiCoV Database (https://www.gisaid.org/, accessed on 5 July 2021) to generate stacked area plots for each region (R-package ggplot2) by plotting the proportions of total number of sequences over time from January 2020 until June 2021, colored by Variants and GISAID Clade’.

## 3. Results

### 3.1. The Pandemic until Summer 2021: Waves of Incidence and Variants

**Figure 2:**
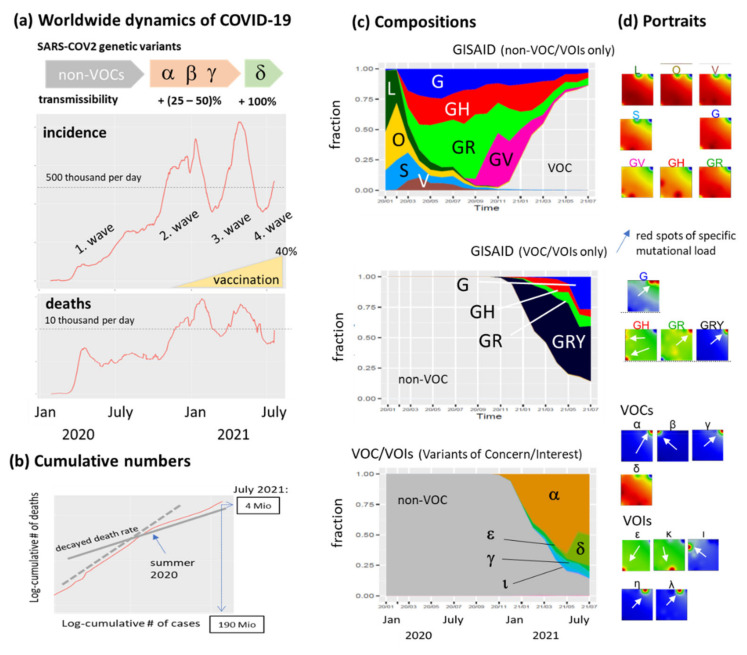
The pandemic in summer 2021. (**a**) Incidence (reported new cases per day) and deaths (per day) worldwide divide into four major waves. The relatively low incidence in the first wave presumably attributes to the relatively small number of tests available until summer 2021. Genetic variants of SARS-CoV-2 evolve roughly from non-VOCs (variants of concern) towards VOCs assigned by Greek letters. The increment of transmissibility is given in units of the effective reproduction number [31]. (**b**) The cumulative number of deaths is plotted as a function of the cumulative number of cases (in double logarithmic scale). The decreased slope after summer 2020 indicates reduced death rates. (**c**) The composition of cases is split according to genetic groups using GISAID and WHO VOC/VOI nomenclatures. Cases from GISAID lineages were separately considered for VOC and non-VOC memberships. (**d**) Mean SOM portraits of the different groups visualize the respective mutational landscapes which are partly similar, especially for non-VOC GISAID classes but markedly different for most VOC/VOI-lineages. Red spot areas of high mutational load are shown by white arrows.

After the first wave of COVID-19 incidence in winter/spring 2020, another two waves of the pandemic were observed worldwide so far, and a fourth wave is presently emerging (Figure 2a). The death toll of the plague directly follows the incidence in similar waves oscillating about 10,000 victims per day worldwide. The plot of cumulative deaths versus incidence increases linearly until summer 2020 which indicates no fundamental improvement of the worldwide situation. The steeper slope until summer 2020 presumably reflects the underestimation of incidence numbers because of relatively small test rates in the first months of the pandemic (Figure 2b).

According to GISAID nomenclature system, most of the currently sequenced SARS-CoV-2 genomes were assigned to one of eight major clades (not clustered genomes were assigned to a ninth clade O), which include the SARS-CoV-2 virus reference strain (clade L) and other early variants and thus better resolving the non-VOC genomes appearing especially in 2020 [29,30]. The initial variants of the virus L, S, O and V were replaced progressively by clades G, GH, GR and GV (Figure 2c). The amount of these early variants S, V, and GH decays with time while GR, GV and GRY (including the VOCs) increases with differences between the geographic regions (see next subsection). Consideration of variants of concern/interest (VOC/VOI) shows that particularly these variants appear in the second half of 2020 and became the dominating ones in 2021. VOCs, assigned by Greek letters according to WHO recommendation [3], were often named by their region of appearance such as the ‘British’ variant alpha, the ‘South African’ variant beta, the ‘Brazilian’ variant gamma and the ‘Indian’ variant delta. While the first three variants partly distribute in parallel (see next subsection), the delta strain drives the fourth wave on global scale. Mean SOM portraits of the different classes were generated by applying machine learning to the data set of nearly 500 variants to visualize their mutational landscapes. The non-VOC portraits are virtually similar and show extended red areas of elevated SNV-load in the left lower part of the map (Figure 2d). In contrast, the portraits of the VOC groups show specific spot areas of increased SNV load (see white arrows in Figure 2d). These changing mutational patterns reflect the fact that the emergence of SARS-CoV-2 in late 2019 was followed by a period of relative evolutionary stasis lasting nearly one year. Since late 2020, however, SARS-CoV-2 evolution has been characterized by the emergence of sets of mutations, in the context of ‘variants of concern’ (VOCs), that impact virus characteristics, including transmissibility and antigenicity, probably in response to the changing immune profile of the human population [1].

### 3.2. COVID-19 in Time and Space

**Figure 3:**
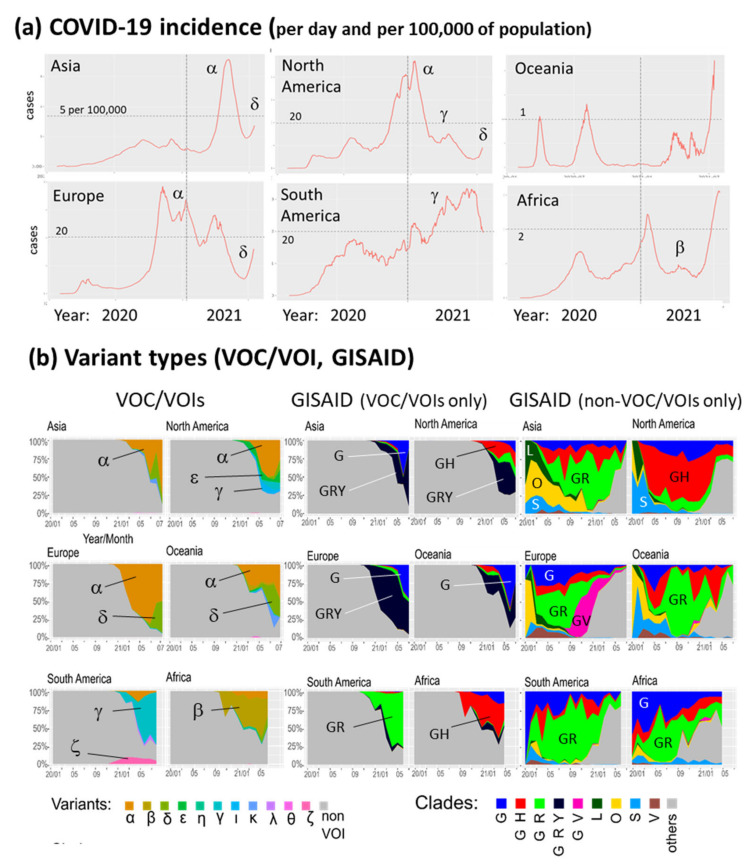
COVID-19 in time and space. (**a**) Incidence (in units of reported COVID-19 cases per day and per 100,000 of population) since January 2020 in different regions of the world. VOCs/VOIs (Greek letters) refer to the most abundant variants taken from part b. Plots were generated using the COVID-19 viewer (https://www.izbi.uni-leipzig.de/current-projects/covid19-viewer/, downloaded at 17 July 2021) [28]. (**b**) Composition of COVID-19 cases regarding VOC/VOIs (left part) and GISAID (right part) grouping schemes. GISAID-clades were separately specified for VOC/VOI and non-VOC containing groups. SOM portraits of the different classes taken from the different regions were shown in Figure A2.

**Figure A2:**
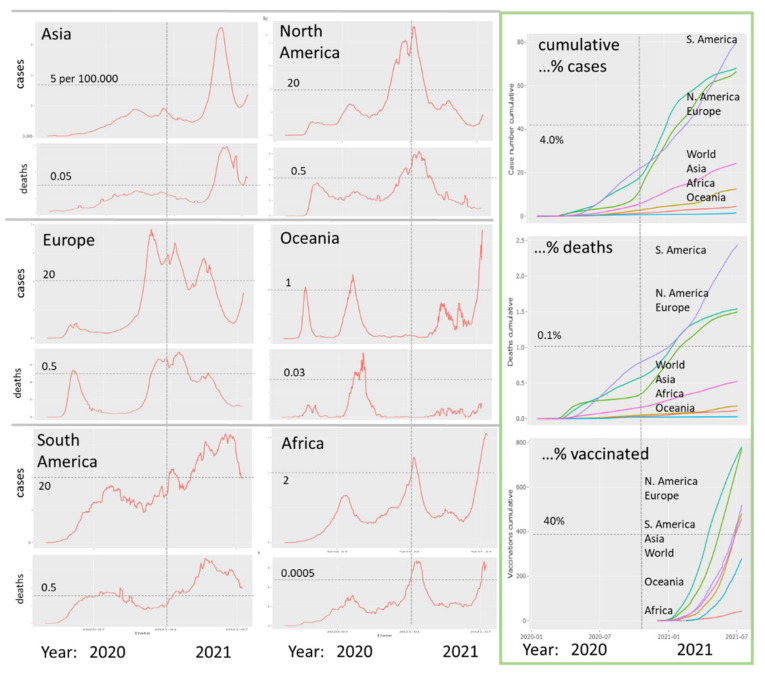
COVID-19 incidences and deaths across the world: number of cases (incidence) and number of deaths (both numbers are counted per day and per 100,000 of population) in different regions of the world were plotted as a function of date since January 2020. One sees the different waves of the pandemic arising, e.g., in Europe in spring 2020 and from autumn 2020 to summer 2021. Cumulative incidences, deaths and percentages of vaccinated people (first shot) across the world are shown in the right (green frame). Graphs were generated using the COVID-19 viewer (https://www.izbi.uni-leipzig.de/current-projects/covid19-viewer/, downloaded on 17 July 2021) [28]. Note: Number of cases before summer 2020 (1st. wave) is biased towards small values because of the relatively small number of tests. Number of deaths roughly followed the number of cases in Asia, South America, Oceania and Africa. In Europe and North America, the deaths in the last (summer) wave 2021 are relatively small presumably because of non-pharmaceutical interventions (NPIs), medical measures and also progressing vaccination. South America is leading the number of cases and of deaths at present. North America and Europe are leading the vaccination (first dose).

**Figure A3:**
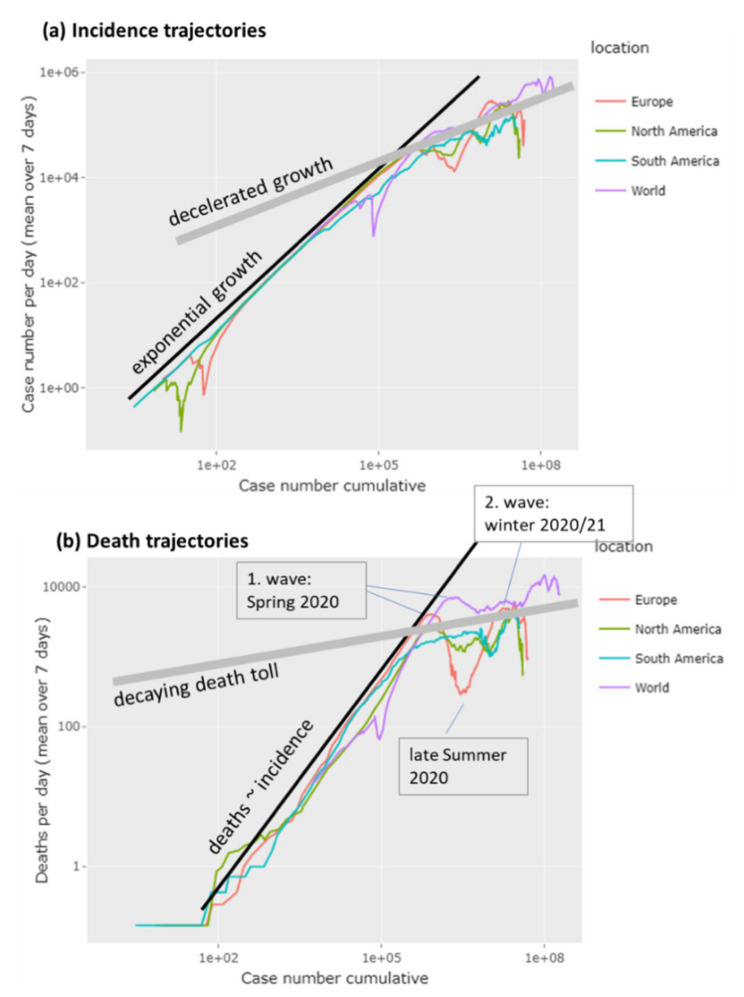
Trajectories of the pandemic in log-log scale worldwide and for selected regions (Europe, North and South America). (**a**) The plot of the number of new cases (per day) as a function of cumulative cases visualizes the exponential character of the pandemic as a line (see [28] for the detailed description of the method). The exponential growth decelerates since summer 2020 owing to NPIs, medical services and vaccination. (**b**) The number of deaths followed the incidence at the beginning of the pandemic (black line) until summer 2020. Then it markedly deviates towards smaller values, but it still grows in absolute numbers (the thick grey line serves as guide for the eye).

**Figure A4:**
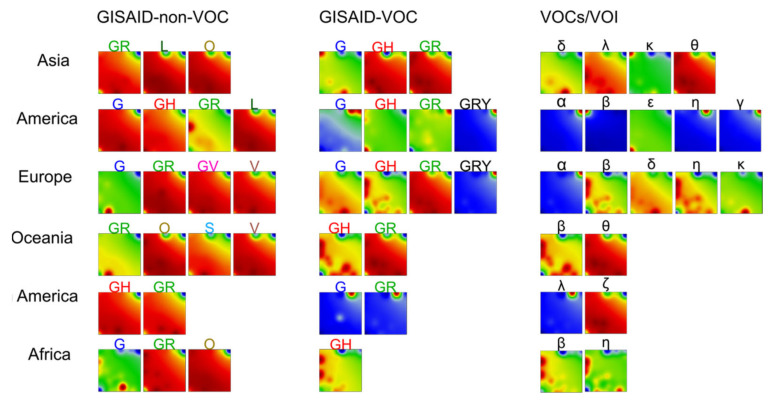
Portraits of the SARS-CoV-2 genomes from different regions of the world for GISAID non-VOC, VOC and WHO VOC/VOI groups. The patterns roughly agree with the overall worldwide portraits (Figure 2d). The portraits of the beta (‘South African’) variant slightly differ between Europe, Africa and North America because of unknown reasons. Lambda (South America) resembles the eta variant.

Next, we resolve the dynamics of COVID-19 incidences (Figure 3a), deaths (Figure A2) and variant composition (Figure 3b) between different regions of the world. The global patterns of the four-waves are modified into specific courses, of, e.g., relatively small incidences in Asia and Africa in 2020 followed by a strong wave in 2021 or of the steadily increasing (until spring 2021) incidence in South America. The death toll virtually follows the courses of incidence as a rule of thumb (Figure A2). Early GISAID-lineages (L, S, O, V) were found in Asia with relatively high abundances, while GISAID non-VOC variants GH and GV appeared specifically in North America and Europe, respectively. Gamma (‘Brazilian’) and beta (‘South African’) VOC lineages distributed specifically in South America and Africa, respectively, while alpha (‘British’) and later delta (‘Indian’) variants dominated in the other regions of the world. Region-specific genomic portraits confirm the global picture (Figure A4). Plots of cumulative data and trajectory views are provided in Appendix B to complete the number-characteristics of the pandemic (Figure A2 and Figure A3). Note also that one observes even inside a certain region marked differences, e.g., between the respective countries. This level of resolution is beyond the scope of this work. However, the interested reader can generate incidence- and death-courses for more than 180 countries based on daily updated data at https://www.izbi.uni-leipzig.de/current-projects/covid19-viewer/.

In summary, dynamics of COVID-19 in space and time show both common and specific features. The pandemic manifested different waves since spring 2020 around the world with ups- and downs in a region-specific fashion, having mostly direct consequences on death toll (Figure A2). In parallel, the SARS-CoV-2 genome mutated giving rise to a sequence of variants arising and being replaced by others afterwards. Variants of concern (VOCs) became dominating since late 2020 where alpha, beta, gamma arising in parallel all becoming presently replaced by the ‘Indian’ delta variant. Often appearance of VOCs is associated with increased incidence reflecting the evolution of the virus towards increased infectivity (transmissibility) and/or better adaption to hosts physiology and virulence [10,32,33,34,35].

### 3.3. SOM Portrayal of the SARS-CoV-2 Mutational Patterns

**Figure 4:**
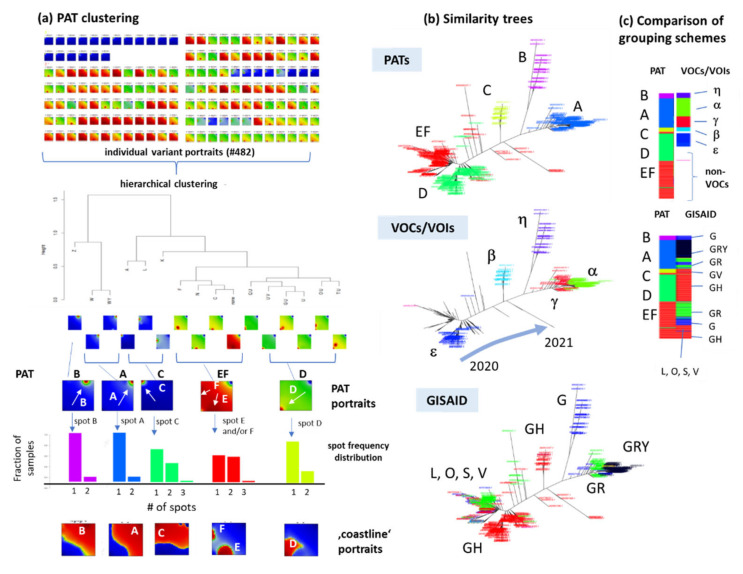
Pattern type (PAT) clustering and comparison with VOCs/VOIs. (**a**) PAT clustering is applied to all variant portraits to obtain five major PATs labelled by letters A–D and EF in agreement with the dominating spot(s) in each of them. Spot frequency distributions reveal that most portraits show only one spot of co-mutated SNV as indicated by the arrows. Coastline portraits use a smoother color scale to better visualize the borderline between positive (red) and negative (blue) values of the SNV score. (**b**) The PATs occupy different branches of the similarity tree, which mostly agree with classification schemes using variants of concern and interest (VOCs/VOIs) labelled by Greek letters and the GISAID clades. The temporal evolution along the tree is indicated by the arrow in correspondence to the composition dynamics shown in Figure 3b. (**c**) Pairwise mapping of the different classes illustrate their mutual correspondence.

**Figure A5:**
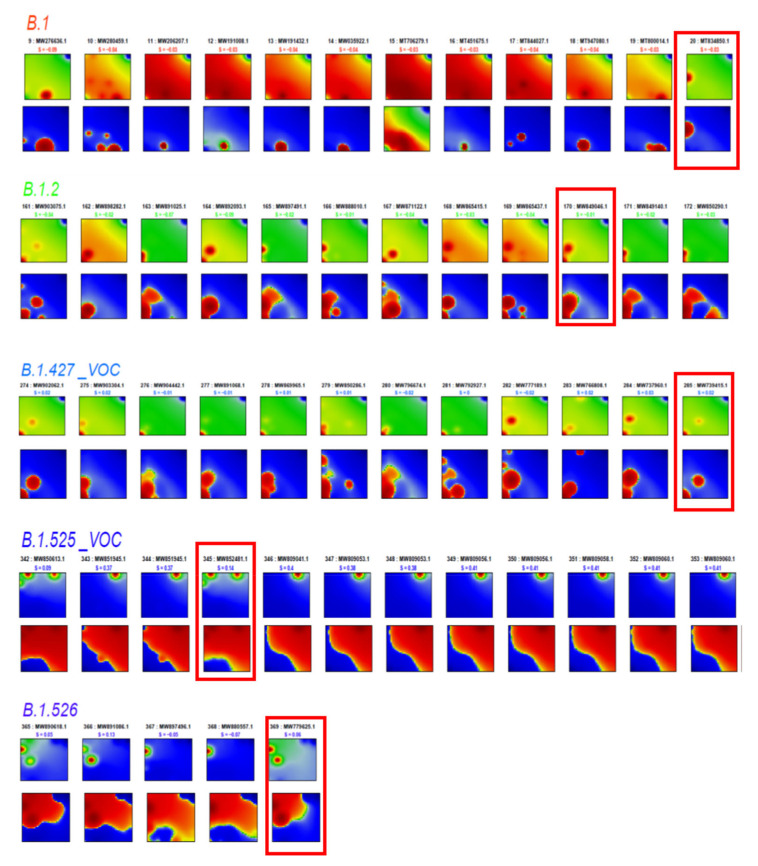
Examples of SOM-portraits of individual SARS-CoV-2 genomes of selected Pangolin-variants in standard and ‘coastline’ scale (upper and lower raw, respectively). The portraits illustrate individual features and partly deviating or even showing outlier properties (red frames). Deviations can appear because of variable mutations, methodical differences (sequencing depths affecting mutations detected) or classification errors. Portrayal thus enables control of the genomes on a ‘personalized’ basis. The full gallery of SOM portraits of SARS-CoV-2 genomes studied is provided in Supplementary Materials: File S1.

Our SOM method provides an individual ‘portrait’ of the mutation patterns of each of the virus genomes explicitly considered in this study. (Supplementary Materials: File S1; examples are shown in Figure A5. The portraits illustrate individual features and partly deviating or even showing outlier properties.). The SOM portrayal method combines supervised and un-supervised clustering in a two-step approach. Firstly, the SNVs were distributed on the quadratic grid of 35x 35 micro-clusters each collecting mutually similar SNV-profiles. These so-called meta-SNVs cluster together into red spot-like areas of high mutational load in the individual portraits owing to the self-organizing properties of the algorithm. These ‘spot-clusters’ collect co-mutated SNVs across the virus genomes. Most of the portraits, especially of the VOC/VOI variants, show only one out of six dominant spots observed in the different portraits (Figure 2d and Figure 4a), which were labeled by capital letters A–F. Clustering of the SOM-portraits provided five major pattern types (PATs) where four were dominated by one of the spots and a fifth one by two spots (see spot frequency distributions in Figure 4a). PATs were named by their dominating spot A–D and EF, respectively, e.g., PAT A type portraits preferentially express spot A and PAT EF types express spots E and/or F (Figure 4a).

Based on the portraits, we generated a similarity tree to visualize their relatedness (Figure 4b). Most of the variants from each of the PATs occupy a separate side branch of the similarity tree which virtually agree with VOC/VOI and partly GISAID lineages, namely, PAT C with beta (β, B.1.351, ‘South-African’ variant), PAT B with eta (η, B.1.525, ‘Nigerian’ variant) and PAT D with epsilon (ε, B.1.427 and B.1.429, ‘Californian’ variant, since July 2021 not further considered as VOI by WHO) (Figure 4b). PAT A splits into two VOCs (alpha and gamma), and GISAID clades (GRY and GR), referring to ‘British’ and ‘Brazilian’ Pangolin lineages B.1.117 and P1, respectively. In summary, data driven clustering of the SOM portraits of the SARS-CoV-2 genomes provides five major pattern types corresponding to accepted classification schemes of the virus. Hereby it was our aim to see whether dimension reduction as provided by SOM modifies previous classification schemes of the SARS-CoV-2 genomes. It turned out that dimension reduction by means of SOM-portrayal virtually preserves accepted classifications of the variants. Mutual relations between PATs, GISAID and VOCs/VOIs mostly, but not always, match (Figure 4c), mainly due to slightly different grouping criteria such as geographic appearance (GISAID), ‘concern’-characteristics and strict genetic similarity (PAT) which will be discussed below.

### 3.4. Relation to the SARS-CoV-2 Genome: Spots and SNV-Floor

**Figure 5:**
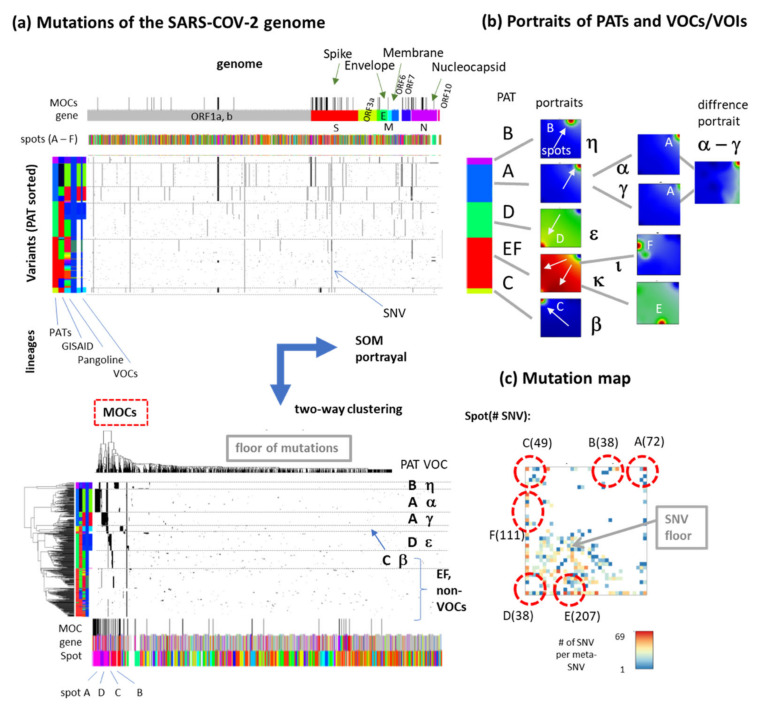
Mutations of SARS-CoV-2 and spot patterns. (**a**) Mutations along the virus genome were grouped by PATs (part above) and by hierarchical two-way clustering (part below). Most mutations of concern/interest (MOCs/MOIs, taken from https://covariants.org/ accessed on 17 July 2021 as non-synonymous mutations across the SARS-CoV-2 genome) group together confirming their relevance to distinguish PATs and VOCs. (**b**) SOM portrayal provides five major PATs, each showing a characteristic portrait with one characteristic spot of co-mutated sequence positions. PATs and VOCs mostly map in a one-to-one fashion except VOCs alpha and gamma both included in PAT A. The difference map indicates a slight shift of the mutational load across the meta-SNV between both VOCs (**c**) The mutation map visualizes the number of SNVs per metagene. Empty metagenes are white. Red circles indicate the spots with the number of included SNVs.

In the SARS-CoV-2 genome, mutations distribute over genes coding basic structural proteins of the virus and ORFs (open reading frames) [36,37,38]. ORFs are defined as contiguous stretches with a start and a stop codon and a ‘protein-coding’ meaning, i.e., translation into a functional protein that contributes to viral transmission, replication, immune avoidance or overall fitness or that can encode an antigen detectable by the immune system or a diagnostic test [39]. The longest, ORF1ab, occupies more than two thirds of the genome. The genes encode the spike glycoprotein (S), the envelope small membrane protein (E), the membrane protein (M) and nucleoprotein (N) (Figure 5a). ‘Mutations Of Concern’ (MOCs) were selected from https://covariants.org/ (accessed on 17 July 2021) as non-synonymous mutations across the SARS-CoV-2 genome. Group portraits are shown in Figure 5b where the portraits of VOCs/VOIs eta, beta and epsilon virtually match the portraits of PATs B, C and D, respectively (see also Figure 4c). PAT A splits into VOCs alpha (B.1.117) and gamma (P1) both showing very similar portraits with a slight shift of the mutational load as revealed by the difference portrait. PAT EF (non-VOC) splits into two major portraits, referring, e.g., to lineage B.1.526 (Iota, ι), expressing spot F and of lineage B.1.617.1 (kappa, κ), expressing spot E. VOCs/VOIs and MOCs cluster together in the heatmap after two-way hierarchical clustering, thus indicating mutual impact of MOCs and VOCs, where the latter ones accumulate in the spots (Figure 5a). Less discriminative SNVs form a sort of ‘floor’ of mutations. The population map visualizes the distribution of SNVs in the SOM (Figure 5c). The mutation floor overall occupies a region in the left lower half of the map while the spots accumulate SNV in localized areas containing between 38 (spot B and D) and 207 (spot E) SNVs (Table A2). Hence, a set of SNV across the SARS-CoV-2 genome accumulates in the spot areas and drives the grouping of variants into PATs and VOCs/VOIs.

### 3.5. Cartography of the Mutational Landscape

**Figure 6:**
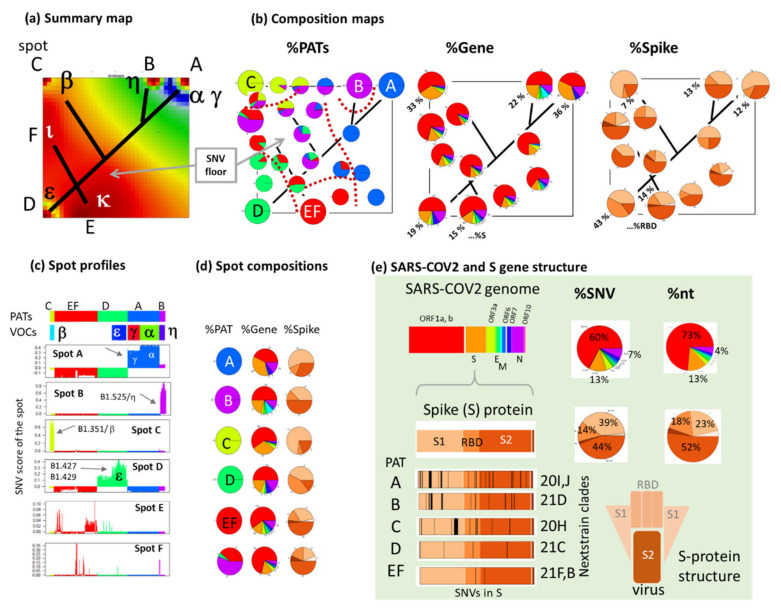
Cartography of the SARS-CoV-2 mutational landscape. (**a**) The summary map visualizes the mutational load across the SOM. The tree inside the SOM serves as a guide for the eye to illustrate similarity relations in analogy to the similarity tree in Figure 4b with WHO (Greek) lettering for VOCs/VOIs. (**b**) The composition maps visualize local composition SNVs regarding PATs, genes and parts of the spike gene across the SOM as pie-diagrams in units of percent. The dotted curves in the PAT-map separate regions of virtually unique PAT composition. The numbers in the %gene and %spike maps indicate percentages of the S-gene and of the receptor binding domain (RBD) in the spot areas, respectively. They vary markedly, e.g., between spot A (right upper corner) and D (left lower corner). (**c**) The spot profiles of the SNV score show the mutational load of the included SNVs across the variants. High load of the spots assigns them to the respective PATs. (**d**) The compositions of SNV regarding PATs, gene and spike region across in the different spots in analogy to part (**b**). (**e**) The legend (green background) assigns the color code for the genes, the regions of the spike protein, the distribution of mutations across the S-gene in the different PATs (SNVs are assigned in Figure A7) and the percentages of SNVs and nucleotides in the different SARS-CoV-2 genes and the S-genes. The plot of the SNV along the S-gene reflects their accumulation particularly in the spike and RBD-parts [1].

Next, we characterize the mutational landscape as provided by the SOM more in detail. The spot summary map visualizes high mutational load across all genomes in red and low load in blue (green refers to intermediate values, Figure 6a). The landscape accumulates MOCs in the spots and distributes floor-SNV in a more extended area. Interestingly, the spots can be positioned along a tree-structure resembling the similarity trees in Figure 4b. It reflects correspondence between the diversity space spanned by the variants and the SNVs, respectively. The composition maps of PATs (calculated as local percentage of SNVs referring to the different PATs and visualized as pie-charts) reveals virtually 100% enrichment of different PATs around the spots except spot F and an area of mixed composition referring to the SNV-floor (Figure 6b, left part and Figure 6d). The percentage of SNVs in the S-gene (coding the S-glycoprotein) is nearly twice as large in spot A (and thus PAT A) compared with spot D reflecting an increase of the relative mutational load in this gene from PATs D, and EF towards PATs A–C paralleled by the decrease of the mutational load in ORF1a,b (Figure 6b and middle and Figure 6d for comparison with the respective percentages across all SNV and nucleotides of the SARS-CoV-2 genome). The percentage of SNVs of the N gene is large in PAT A, B and D indicating subtle shifts between the different genes as a result of evolutionary adaptation [39]. SNVs in N involve a B-cell epitope, suggesting immune-avoidance selection [39,40]. The S-genes divides into different parts, namely, S1 coding the ‘spike’ (pointing towards the host, see scheme in Figure 6e) and including the RBD (receptor binding domain), as well as the S2 region anchoring the protein in the virus membrane. The RBD, in particular, is the target of most therapeutics and is the major antigen against which the virus-host innately generates neutralizing antibodies [10].

**Figure A6:**
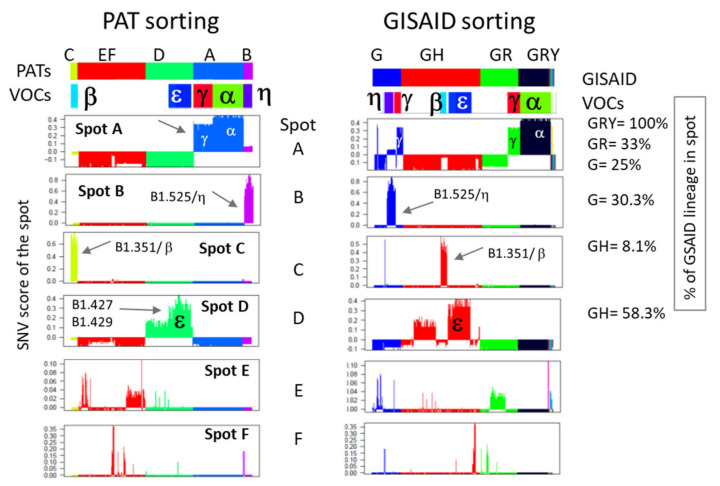
Spot SNV-profiles across the variants sorted and colored using PAT (**left**) and GISAID (**right**) classes. WHO VOC/VOI classes are indicated by the second color bar to link both sides. VOC/VOI classes uniquely assign to PATs except PAT A which splits into VOCs alpha and gamma. In contrast, the relation between GISAID and VOC/VOI groups is fuzzier. For example, gamma splits between GISAID GR and GH, which, in turn, contains also epsilon and beta.

Percentage of SNVs in S1 markedly increased in PAT A and C compared with the other PATs. Hence, detailed segmentation of the SOM with respect to the mutational load and distribution of SNVs across the PATs and genes of the SARS-CoV-2 genome characterize the mutational landscape of the variants in a systematic fashion. The spot profiles of the SNV score provide a perpendicular view on the landscape across the variants: a high score value is found for the enriched PATs and VOCs/VOIs (Figure 6c). The profiles reveal also differences between VOCs alpha and gamma (systematically smaller score) in PAT A and of VOI epsilon in PAT D (score biased towards larger values). Resorting of variants according to the GISAID nomenclature links it with VOC/VOI and PAT groupings and indicates the partly fuzzy relationships (Figure A6). In summary, SOM provides a highly resolved mutational landscape showing the distribution of SNVs across the SARS-CoV-2 genome and their appearance in the different virus types.

### 3.6. SNV Mapping of the SARS-CoV-2 Genes

**Figure 7:**
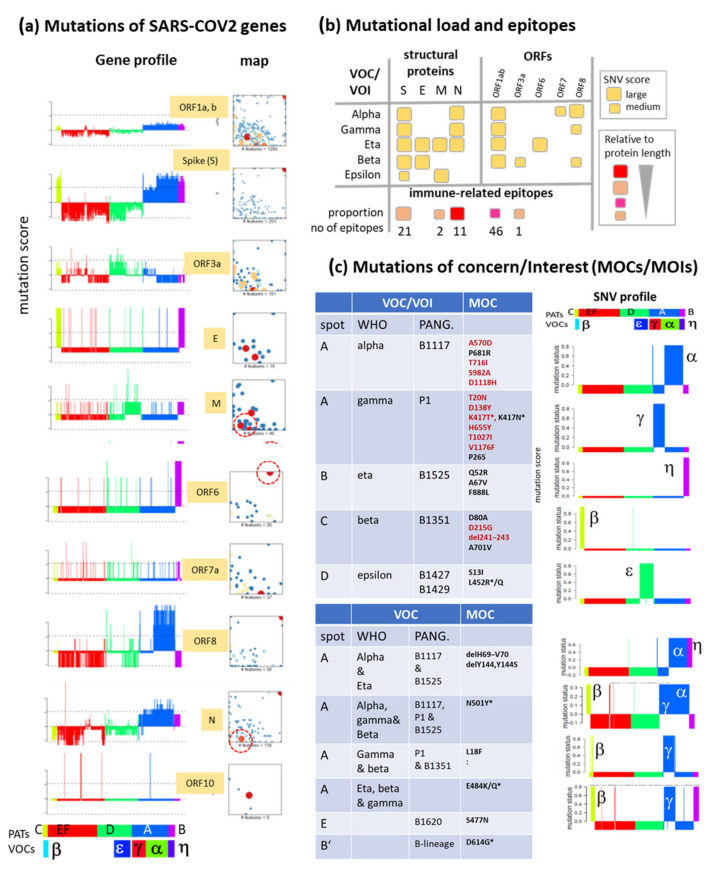
Mapping mutations. (**a**) SNVs from the SARS-CoV-2 genes are mapped into the SOM and depicted as SNV-score profiles sorted using PATs and VOCs/VOIs. Specifically increased mutational load is found for the different genes except ORF7a and 10. (**b**) Summary of the mutational load and of immunogenic epitopes of structural proteins and ORFs across the variant types as estimated using the SNV-score and data taken from [40], respectively. The spike and nucleocapsid proteins are most affected by SNV. VOI eta (PAT B) shows widest effect across the SARS-CoV-2 genome. Epitopes are enriched in N, S and orf1a/b. (**c**) Mutations of concern (MOC) were selected from https://covariants.org/ accessed on 17 July 2021 (Figure 5a). Spot location and VOC/VOI(s) showing the mutations (only S-gene) were listed. Part of SNVs appear either in one VOCs/VOIs (table above) or combinations of them (table below) as illustrated by their SNV-score profiles. Red text color marks high-confidence SNVs taken from [39] (Figure A8), asterisk marks MOCs as assigned in https://www.nytimes.com/interactive/2021/health/coronavirus-variant-tracker.html#Q677.

**Figure A8:**
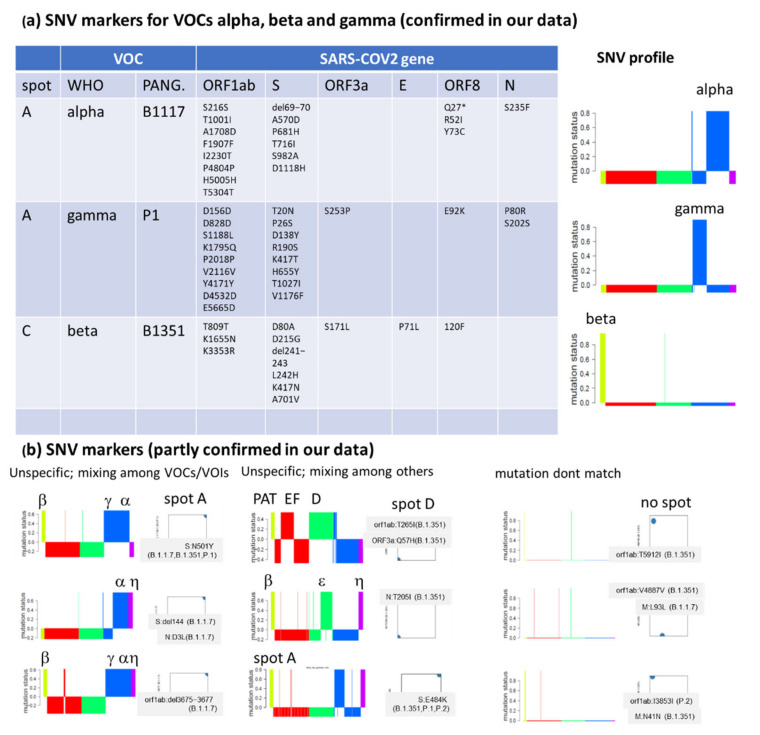
Map of the high-confidence gene set of mutations taken from [39]. (**a**) Most of the SNV are confirmed in our data as specific for the intended VOC. The respective SNV profiles show mutational load in respective variants. (**b**) A small fraction of suggested SNVs are not confirmed in our data showing mutations in more than one VOC/VOI, in different PATs or even lack marked mutational load.

Gene-wise SNV-maps and -profiles provide information about their distribution in the mutational landscape and biased appearance in the different PATs and lineages (Figure 7a). SNV of most genes are found either in different spots or the area of the SNV-floor. These distributions in the SNV-landscape transform into mean SNV-profiles of the different genes reflecting their mutational load across the variants. For example, the profiles of the S- and the N-genes resemble a combination of the profiles of spots A–C with increased mutational load in the respective PATs A–C (compare with Figure 6c), which reflects the enrichment of mutations in the S- and N-genes (and, partly also in ORF1a,b) in the VOCs/VOIs alpha, beta, gamma, eta see also Figure 6b, middle). The E-gene shows a high SNV-score in PATs B and C (VOC/VOIs eta and beta, respectively) and the M-gene in PATs B and D (VOC/VOIs eta and epsilon, respectively). ORF8 shows a specifically enlarged SNV-score in PAT B (VOI eta) and ORF8 in PAT A (VOC alpha). ORF10 lacks specific association with PATs or VOCs/VOIs. The overview table in Figure 7b reveals that the S- and N-genes are widely mutated across the VOCs/VOIs. VOI eta (PAT B) shows broadest mutational load across the genes encoding the structural proteins of the virus. Interestingly, these distributions resemble the distribution of a high-confidence protein-coding gene set obtained recently by comparative genomics to consider evolutionary constraint, and to prioritize functional mutations [39] (Figure A8). Single SNV-profiles of the MOCs of the S-gene assign their appearance in single VOCs/VOIs or combinations of them (Figure 7c). For example, deletions delH69 and delH70, both located in spot A, are found in VOCs/VOIs eta and alpha while SNV T20N appears in VOC gamma only. Notably, immunogenic epitopes targeting hosts immune response enrich in the N-, S- and also ORF1a,b genes and associate with the high mutational load especially in the alpha, beta and gamma VOCs [40] suggesting their immune evading potency (Figure 7b). Note also that genes without structural impact can host such epitopes and play roles in immune response.

In summary, SNV maps and profiles of the SARS-CoV-2 genes reveal mutational hotspots in the different variants with potential functional impact related to evolution-driven virulence, transmission and/or immune evasion. Mapping of a set of high-confidence SNV markers mostly from the ORF1a,b- and S-genes for different VOCs to the SOM confirms this view [39] (Figure A8).

### 3.7. Development SARS-CoV-2 in Variant and SNV Space

**Figure 8:**
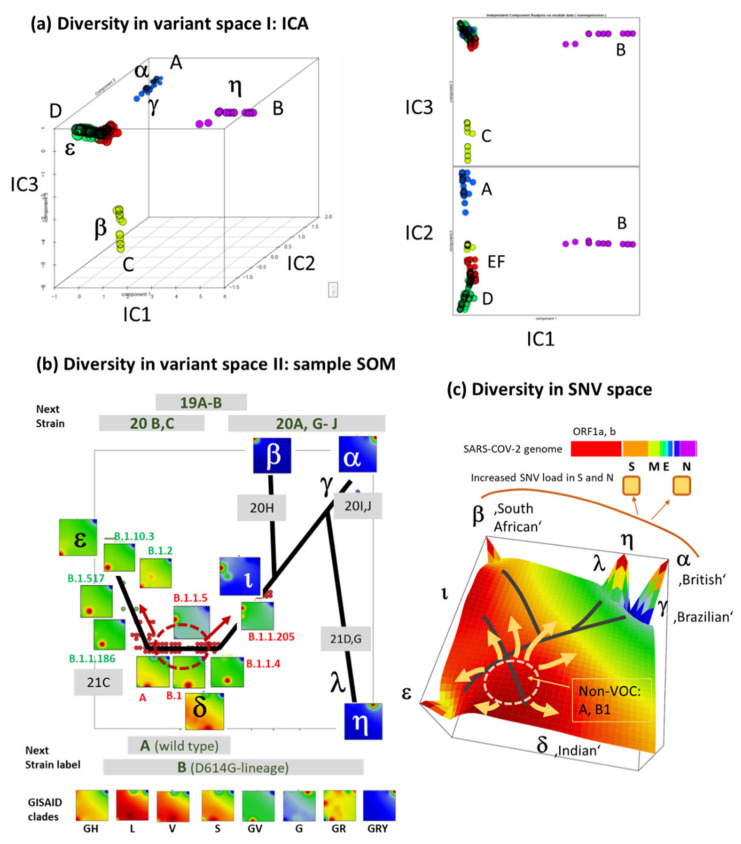
Similarity landscapes of SARS-CoV-2 genomes in variant and SNV space. (**a**) Independent Component Analysis (ICA) revealed that genomes of PATs A, B and C evolve each along one of the first three independent components IC1–IC2 indicating mutual independent mutational patterns. VOC gamma (‘Brazilian’ variant) is followed by alpha (‘British’ variant) along IC2. (**b**) The sample SOM distributes variants in two dimensions in non-linear scale to better resolving details of the non-VOC root areas of SARS-CoV-2 evolution. Early SARS-CoV-2 variants (GISAID clades L, V, S) locate in the center (red ellipse). Development proceeded towards PAT D to the left and A, B, C to the right. PATs A–D refer to VOCs/VOIs as indicated in part a of the figure. The tree (black lines) is adapted from Figure 4b. NextStrain nomenclature is indicated using grey background, where A-labeled clades refer to the early detected ones. The number indicates the year of first report and the letter is the count of variants reported, e.g., 20H assigns the South African variant B.1.1351 as detected in 2020 just before the Brazilian (20I) and British (20J) ones. Spike-protein substitutions D614G defines an early SNV producing the dominant pandemic forms of the virus [39]. (**c**) The summary SOM visualizes the mutational landscape in SNV-space. VOC/VOI-related SNV protrude as ‘peaks’ of their SNV-score while the root region of non-VOC SNV referring to the SNV floor form an extended area of moderately enhanced SNV-score serving as root area of the evolving VOCs/VOIs. Increased mutational load is observed for the structural protein genes S and N (Figure 7b). See also Table A2 for the gallery of portraits (Pangolin classes).

In the next step, we tried to describe development of the SARS-CoV-2 genome from its early root variants towards the VOCs/VOIs appearing in the last months. Above, we applied phylogenetic tree analysis to visualize similarities between the different SARS-CoV-2 types and their dynamics (Figure 4b). In addition, we applied independent component analysis (ICA) of the SOM portraits, which overall reveals another interesting detail: PATs A, B and C (and the respective VOCs/VOIs alpha/gamma, eta and beta) each distributes along one of the first three independent component coordinate axes (IC1–IC3) meaning that their genomes evolve virtually independent of each other (Figure 8a). This result is not really surprising because each of these three PATs is characterized by only one major single spot of co-mutated SNVs which do not mutually mix and thus appear virtually independently (Figure 4a). The same argument applies to PAT D (VOI epsilon) which distributes roughly along IC2 as PAT A, however, over a much smaller distance and in opposite direction.

To better resolve the root area of SARS-CoV-2 variants involving the early split region, we applied the so-called sample SOM, which applies SOM to the collection of virus variants instead of to their SNV [15]. The sample SOM obtained provides similarity relations in variant space. Because of the non-linear scaling, this map ‘amplifies’ the area occupied by PATs EF and D collecting non-VOC variants [15] (Figure 8b). Particularly, the non-VOC area forms a sort of source from where the viral genomes developed towards different directions, namely towards PAT D/VOI epsilon and towards PATs A, B, C/variants alpha, gamma, eta and beta, respectively. Detailed inspection of the portraits of selected variants revealed that the developmental tree in variant space (Figure 8b) transforms into a similarly-shaped tree in SNV-space pointing towards SNV arising in the VOCs/VOIs (Figure 8c). These latter SNVs locate in ‘peaks’ of high specific mutational load while the root area distributes over a wider range assigned as SNV-floor. Hence, the two different similarity plots in variant and SNV space visualize two closely related, but different aspects of the evolving SARS-CoV-2 genomes. In summary, profiling of the mutational load of the different genes indicates mutational drifts with potential functional impact such as increased transmissibility (e.g., promoted by the S-gene) or immune evasive functionalities (see below).

### 3.8. Pseudotime Describes Development of the Virus Genomes

**Figure 9:**
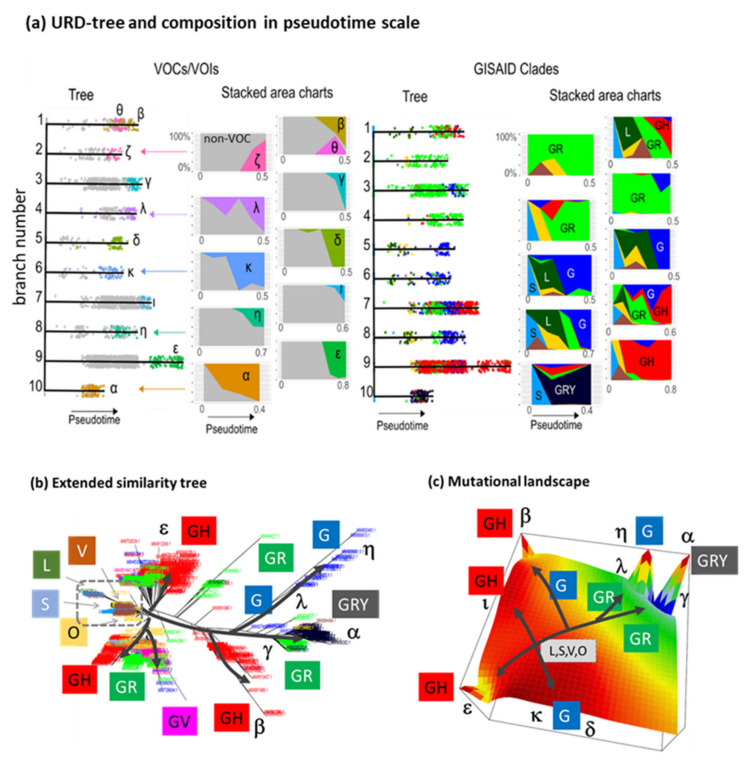
Pseudotime (PT) analysis of SARS-CoV-2 development. (**a**) Tree sorts the variants along ten branches each leading to one VOC/VOI. The variants were colored using WHO VOC/VOI (left part) or GISAID groups. The stacked composition plots reveal that the non-VOC variants were progressively replaced with VOC variants with increasing PT in a branch specific fashion. (**b**) The similarity tree reflects development of the virus in direction of the arrows. For better resolution, the number of SARS-CoV-2 genomes was increased to 1241 using xSOM (see next subsection). (**c**) Development in SNV-state space between GISAID clades. See also Figure A9 and Figure A10.

**Figure A10:**
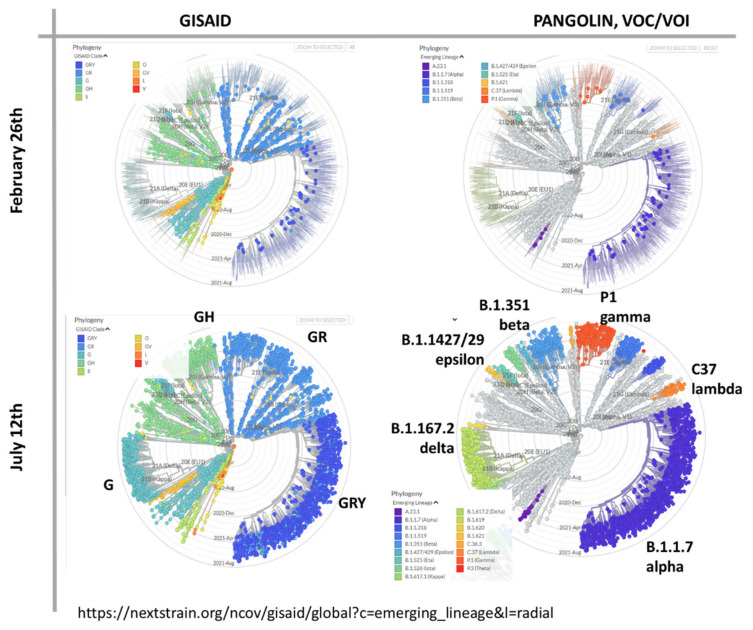
Radially displayed phylogenetic tree of of SARS-CoV-2 available in February (**above**) and July 2021 (**below**). The various types are color coded (using GISAID (left part) and PANGOLIN (right part) nomenclatures (Source: https://nextstrain.org; 27 July 2021). Only GSAID lineage GRY virtually one-to-one matches with VOC alpha. The others VOCs/VOIs distribute over different GISAID lineages. Our SNV-spot profiles (Figure A6) reflect these relationships.

**Figure A11:**
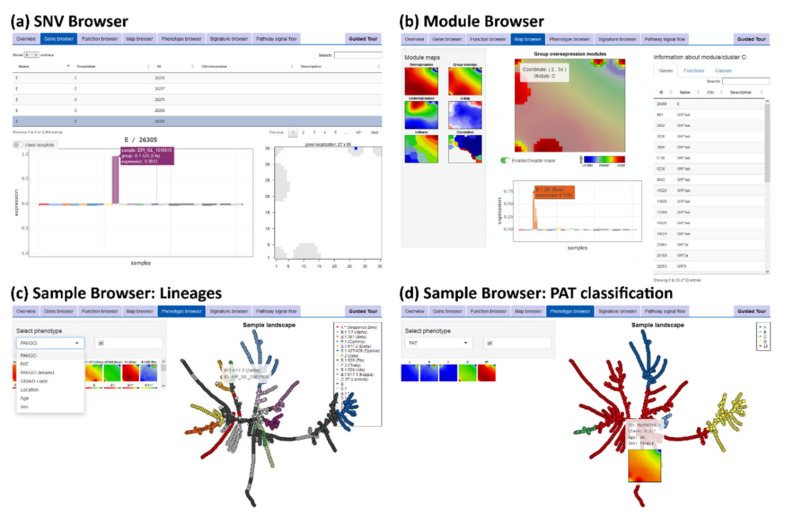
Screenshots of the different oposSOM browser functionalities to explore the SARS-CoV-2 variants data. (**a**) The gene browser shows the SNV profiles and mapping into the SOM grid (localization) of selected sequence positions. (**b**) The module browser provides access to the different SOM segmentation methods and shows spot-cluster SNV profiles and associated SNV for the interactively selected spot. (**c)** The sample landscape shown in the phenotype browser can be grouped according to a selection of different phenotypes. Here the PANGOLIN classification is shown. Hovering the nodes of the landscape provides the variant ID and the corresponding class. (**d**) The sample landscape grouped according to our PAT classification. Clicking any node in the graph (i.e., a particular variant sample) reveals further information and the respective meta-SNV portrait. See also Figure A12.

**Figure A12:**
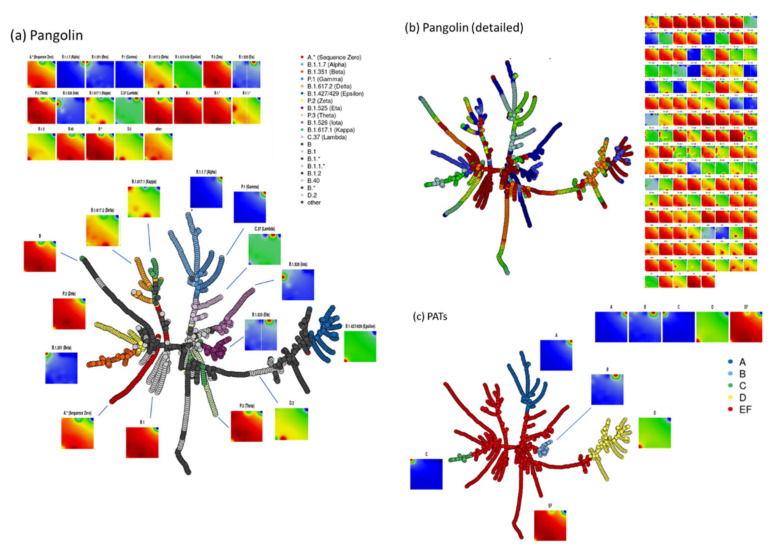
Exploring the spanning tree in the oposSOM browser (see also Figure A11d). The tree is a similarity presentation of SARS-CoV-2 variants enabling an alternative view to phylogenetic tree, second level SOM and independent component analysis (see main paper). (**a**) Tree is colored according to pangolin classification. Group portraits around the tree are shown for VOCs/VOIs and early lineages A and B. (**b**) The tree is colored according to detailed pangolin classes. Their portraits are listed on the left. Please use the browser tool for detailed inspection. (**c**) The tree is colored according to PATs. PATs A, B, C and D accumulate at the tips of side branches while PAT EF forms the central part.

Pseudotime (PT) subsumes bioinformatics analysis concepts to extract dynamic information from cross-sectional omics data [41]. It is based on similarity measures between the virus genomes in multidimensional SNV-space which is downscaled into a low dimensional directed tree topology. We applied the URD-method [24] to describe developmental paths from non-VOC root groups towards different VOC/VOIs lineages. It provided ten branches 1–10 which are also assigned by Greek letters according to the final VOC/VOI states accumulating at the end of the branches (Figure 9a). The composition plots of the branches as a function of PT indicate the progressive growth of the VOC/VOI types at higher PT-values. Coloring using GISAID-nomenclature enabled an alternative view which particularly resolves ‘early’ root variants and links them with the ‘late’ VOC/VOI types. Overall, one finds four groups of GISAID-specific branches governed by clades GR (branches 2–4), G (5, 6, 8), GH (1, 7, 9) and GRY (10), where each branch is characterized by its own specifics of dynamically changing composition. The URD-tree topology maps onto the phylogenic similarity tree (Figure 9b), which has been extended by additional variants using xSOM (see next subsection) to better resolve details, especially in the root area referring to GISAID-clades L, S, V and O. Accordingly, PT-development proceeds mainly from root area at the left to the VOC-tips at the right. As already mentioned in the previous subsection, the obtained tree-topology is reproduced in the mutational landscape (Figure 9c). The GR-clades occupy pre-tip areas in both, sample and SNV space in agreement with the nextstrain-tree analysis (Figure A10). Note also that the PT-range is largest for branch no. 9 leading to VOI epsilon and shortest for branch no. 10 ending in VOC alpha. This scaling corresponds to the number of genomes passed in-between, meaning that a larger PT-range reflects evolution in ‘small’ steps while the shorter PT-range associates with evolutionary hops due to the underlying functional gains in virus fitness. In summary, PT-analysis enabled a view of developmental paths of the virus which links the different nomenclature schemes and scales development in a sort of ‘fitness’ measure inversely related to the PT-increment.

### 3.9. Extending the Data: xSOM

**Figure 10:**
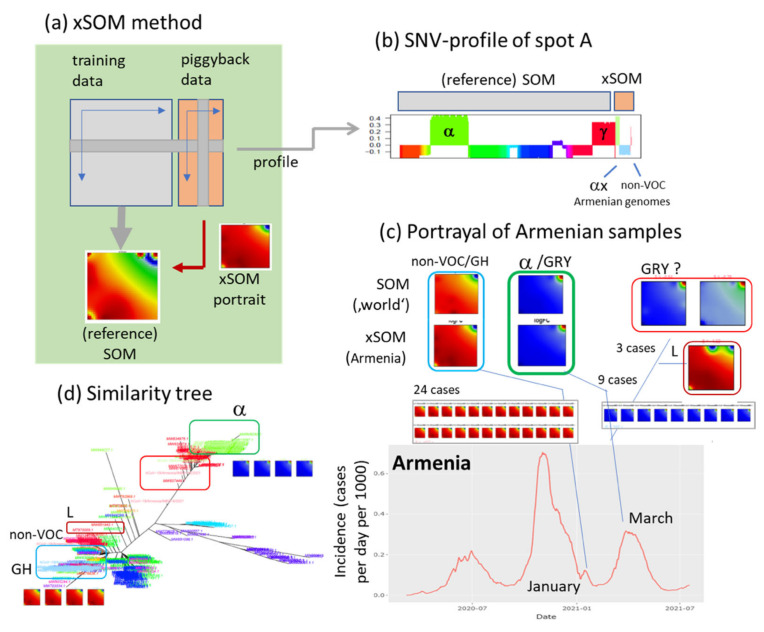
Adding new variants to an existing SOM (extension SOM, xSOM). (**a**) The xSOM method uses an always trained SOM as reference (here our 483 variants were used for initial training, Figure 1). SNVs of the genomes of new variants were distributed among the meta-SNVs according to the reference SOM. Their meta-SNV scores were adapted such that they meet the criterion of minimum *Euclidian* distance. For each of the new variants one gets a xSOM portrait. (**b**) The profile, e.g., of spot A, splits into variants of the reference SOM and of the xSOM. (**c**) The genome SARS-CoV-2 portraits of 36 COVID-19 patients collected in Armenia in January and March 2021 assigned to non-VOC/GH and predominantly to the ‘British’ variant (alpha/GRY) by comparison with ‘world’-reference portraits, respectively [26]. (**d**) Their location in the similarity tree confirms these assignments.

SOM is trained based on a set of genomes referring to a certain deadline-date and based on a certain selection of ‘individual’ virus genomes. There is interest to consider new cases collected after the deadline date to estimate evolving virus genomes or to add variants from the past not explicitly considered in the training data. As a first option, one can train a new SOM based on the completely new data. That would, however, require the full new analysis of SOM topology including spot patterns, their profiles and the distribution of SNV across the meta-SNV. As an alternative option, we developed the extension SOM (xSOM) method which maintains the always existing SOM and fits new data to its topology in a sort of piggyback approach (Figure 10a). We applied xSOM to generate portraits of a series of variants not available in the primary data such as VOC delta (Table A1). For a worked example of xSOM, we made use of 36 SARS-CoV-2 genomes sequenced in Armenia in spring 2021 [26]. Twenty-four variants collected in January were assigned to non-VOC genomes (GISAID GH clade) and 9 out of 12 variants collected in March to the ‘British’ alpha variant (GISAID GRY-clade), which becomes obvious always after visual inspection of their individual portraits (Figure 10b,c). One of the remaining cases assigns to non-VOC L-clade and the two others resemble the alpha-variant, however, with slightly modified mutation patterns which presumably results from insufficient sequencing depth and shortcomings of nanopore sequencing [26]. Hence, xSOM provides an option to add new samples of virus genomes to the presented SOM either retro- or prospectively, however, under the restriction that novel genomes differ not too largely from the reference genomes.

## 4. Discussion

### 4.1. Trade-Offs Shaping the Diversity and Evolution of SARS-CoV-2

Mutations of the SARS-CoV2 genome are increasingly documented around the world, enabling systematic views on the evolving virus. They were categorized by different nomenclature schemes: GISAID applies a 9-level labeling for major clades based on phylogenetic marker mutations from the early variants S, L, V and O via G and GH towards GR and GRY. Nextstrain uses a Year-Letter nomenclature to label clades that persist for at least several months and have significant geographic spread [6]. The Pangolin scheme aided in the understanding of patterns and determinants of the global spread of the pandemic strains by a broad-brush categorization of globally circulating diversity using a not-easy to pronounce letter-number code such as B.1.117 or B.1.351 [19]. For the sake of simplicity, these latter variants were re-named by many media outlets, e.g., as ‘British’ and ‘South African’ variants, respectively. Part of Pangolin-variants were then assigned as ‘variants of concern’ (VOCs), others as ‘variants of interest’ (VOIs) or ‘Variants of high consequence’ (https://www.cdc.gov/coronavirus/2019-ncov/variants/variant-info.html accessed on 17 July 2021) to characterize their potential impact on critical SARS-CoV-2 countermeasures. To quell this sort of ‘Babylonian confusion of tongues’ regarding virus names and to avoid geographical stigmas, SARS-CoV-2 variants got Greek letters in May 2021 by WHO [3], e.g., alpha and beta for the ‘British’ and ‘South African’ VOCs, respectively, not to replace scientific labels but mainly to serve as a handy shorthand for non-experts who are increasingly losing track of different variant names.

**Figure 11:**
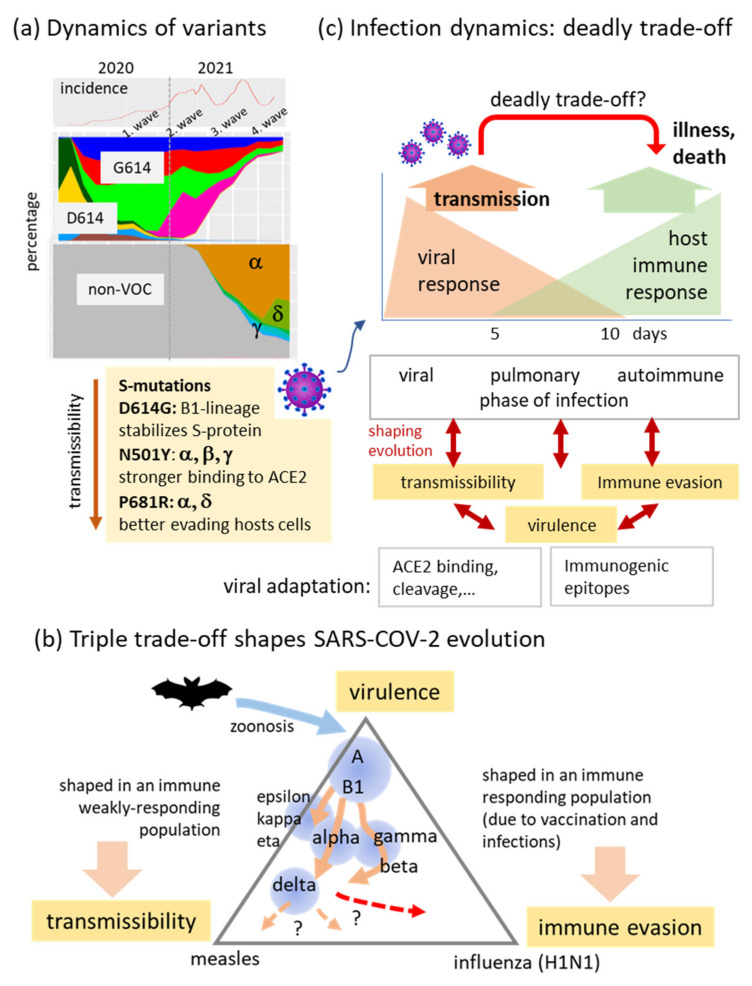
Understanding the past and predicting the future of COVID-19? (**a**) Dynamics of variants reflect a sequential ‘hostile takeover’ related mainly to increased transmissibility (infectiousness) after mutations of the spike protein: D614G made SARS-CoV-2 a bit more infectious thus promoting spread of the virus around the world causing the first wave of pandemic in 2020. The following waves of incidence relate to VOCs which replace each other often in serial order mainly due to increasing transmissibility. (**b**) Evolution of SARS-CoV-2 can be roughly understood in terms of a trade-off between three factors, virulence, transmissibility and immune evasion (reduced neutralization sensitivity): Zoonosis (presumably) from bat to humans causes the initial outbreak in Wuhan (A-lineage) followed by the spread over the world of B-variants and later in 2020 by mutational diversifications into VOI/VOCs mostly driven by increasing transmissibility but also affected by immunogenic adaptation. VOCs gamma and beta seem to better evade immune response than alpha and VOIs but all became virtually replaced by the highly infectious delta variant during 2021 [44]. Future developments are difficult to predict. Possible ways can lead towards further increasing transmissibility (measles-like behavior) or towards immune evasive variants (influenza-like, red dashed arrow). In an immune-responding population, the latter options seem more probably requiring repeated vaccinations to overcome escape variants. See text and [44] for a detailed discussion. (**c**) Evolution of SARS-CoV-2 can cause more severe variants if transmission from infected persons takes place before severe illness as observed for COVID-19 with an early viral response phase followed by pulmonary and later an autoimmune phase related to long-COVID and, in worst case, death [51].

In this publication, we first documented the serial replacement of the different variant classes since the early stadium of the pandemic on worldwide scale and its specifics in different regions of the world. Changing genomes reflect virus evolution towards increased fitness, first of all, in terms of increased transmissibility due to mutations of the spike protein (Figure 11a). The intrinsic reproduction number of the non-VOC of SARS-CoV-2 is about R_0_ = 2.5–3 [42,43] and thus slightly larger than that of common cold. The reproduction number however gained by (10–50)% for the VOIs, by (25–30)% for VOCs alpha and beta, by 50% for gamma and by about 100% or even more for delta [31] thus reflecting increasing transmissibility in the course of virus development. Delta is on a transmission-level comparable with Chicken Pox and thus roughly twice as contiguous as the early SARS-CoV-2 variants and more infectious than Ebola and Pox (https://www.nytimes.com/2021/06/22/health/delta-variant-covid.html; https://www.bbc.com/news/health-57431420).

**Figure A13:**
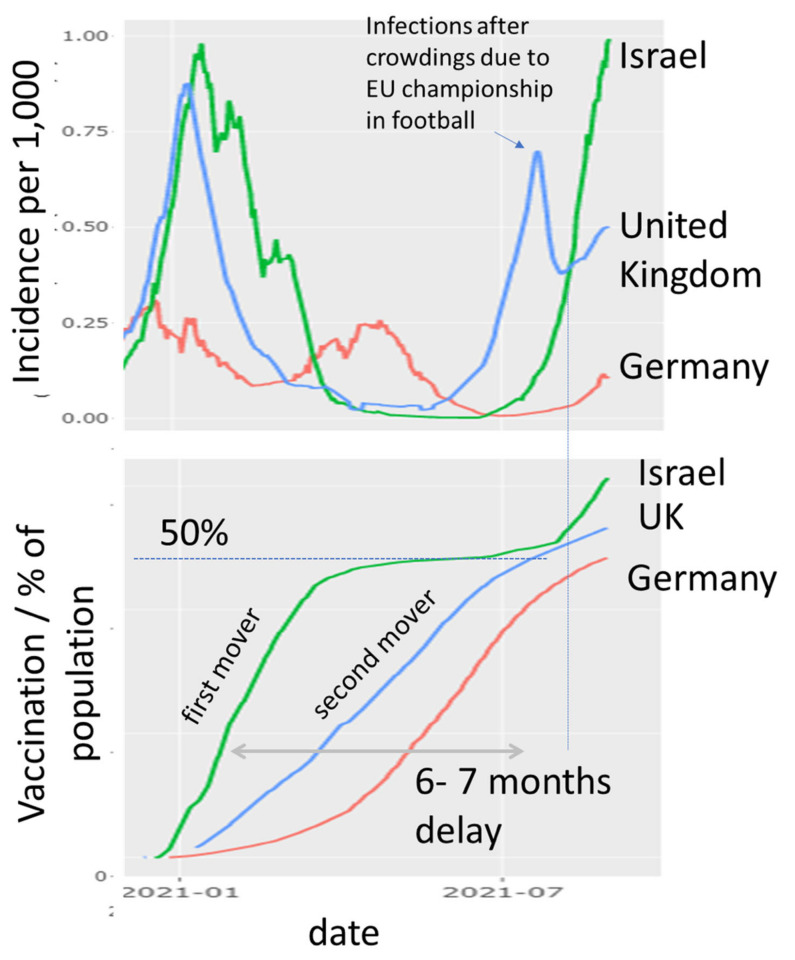
Breakthrough infections after vaccination? Israel was the first vaccination mover reaching vaccination of about 50% of population in February–March 2021. About 6–7 months later the incidence of COVID-19 (delta variant) strongly gains because of unclear reasons. Possible factors are: (i) The amount of vaccination remained clearly below herd immunity level (>80%). (ii) Insufficient and/or waning protection due to immune evasion of SARS-CoV-2 delta. A recent study demonstrated that natural immunity confers longer lasting and stronger protection against infection, symptomatic disease and hospitalization caused by the delta variant of SARS-CoV-2, compared to the BNT162b2 (Biontech-Pfizer) two-dose vaccine-induced immunity [53]. The waning protection is possible due to the too short interval between first and second dose (3–6 weeks) [40]. Delayed intervals of 8–12 weeks provide much higher antibody titres reflecting stronger humoral and cellular immune responses Moreover, the ‘second moving’ country UK shows an increasing incidence after a first peak, which associated with crowds gathering during the European Cup in football. These facts possibly reflect immune evasion of the virus leading to waning immune response which raises the need of repeated booster vaccinations. Plots of incidence and vaccination data were taken from https://www.izbi.uni-leipzig.de/current-projects/covid19-viewer/ accessed on 30 August 2021.

Evolution of the virus can be interpreted as a triple trade-off between virulence (driving zoonosis presumably from bats to humans and also spread of the virus in the first wave of the pandemic), transmissibility (driving the following waves as a series of VOCs/VOIs) and immune evasion with possibly increasing impact in future [44] (Figure 11b). Immunogenic epitopes were identified especially in the N-, S- and partly ORF1a,b proteins showing high mutational loads in the VOCs which presumably shape adaptation of the virus to hosts immune response, especially in the beta, gamma and partly alpha variants [44]. The fatality rate, on the other hand, remained virtually unchanged so far (0.1–1% of diseased persons averaged over all ages) [45] but decays dramatically below 0.001% after full vaccination [46] (https://old.reddit.com/r/dataisbeautiful/comments/osqt5c/oc_covid19_infections_serious_unvaccinated_vs/). Moreover, vaccines seemed to be highly effective at preventing symptomatic and severe COVID-19 including the delta variant [47]. Full vaccination rates of about 50% in Europe and North America are presently opposed by more than two times lower rates in the other parts of the world and still far away from herd immunity (>80%). A recent publication suggested eradicability of COVID-19 without reaching herd immunity by high vaccination coverage combined with SARS-CoV-2 variant control to avoid vaccine-escape [48]. Newest data from Israel, the ‘first vaccination mover’ worldwide, on the other hand, report a new wave of incidence roughly 6–7 months after more than 50% of the population obtained the second vaccination shot (Figure A13). Although the reasons are still not clear, the evolutionary trajectory seems to turn towards immune evasion in an influenza-like scenario and/or waging hosts immune response requiring regular adaptation of vaccines to new variants such as the C.1.2. lineage associated with an increased substitution rate, as was previously observed for the VOCs [49]. Moreover, trends towards less or more severe illness are unpredictable. SARS-CoV-2 is replicating in the upper airways, whereas serious disease, if it develops, comes later, which can make the host sicker maintaining spread just as fast or even faster as before (Figure 11c). Overall, these facts underline the importance of sequence-based surveillance of the pathogen with high temporal and regional resolution and using meaningful nomenclatures based on the genetic relatedness of the sequences to enable their simplified tabulation for integration with epidemiological analysis [50].

### 4.2. Cartography of the Virus Genomes

We applied machine learning by means of Self Organizing Maps (SOM) to portray the mutation patterns of SARS-CoV-2. The method provides ‘personalized’ images of individual virus genomes, which can be simply compared by visual inspection without deeper knowledge of the composition and function of the SARS-CoV-2 genome. We generated mean portraits for classes of different nomenclatures thus visualizing the genomic relatedness between them. Especially, the portraits of the most VOCs and VOIs reflect specific mutational patterns differing from the non-VOC strains, which, in turn, are mutually more similar each to another. The ‘portraits’ of the SARS-CoV-2 classes show specific color patterns visualizing differences in their genomes. We re-classified them using a strictly pattern-driven approach proven in previous SOM-portrayal applications [13,14]. We applied this PAT-classification to the virus genomes not to further increase the ‘Babylonian confusion of tongues’ regarding virus genomes but to judge their diversity as seen by the SOM-portraits. The obtained PATs well reflect VOC/VOI classes in most cases. SOM-portrayal adequately reduces dimension of the data by a factor of thousand and visualizes the virus genomes in an acceptable fashion.

The different portraits were subsumed into a mutational landscape of SARS-CoV-2, which for the first time cartographies the SNV-space of the virus. It resembles the relatedness between the variants in variant-space but, in contrast, visualizes the relatedness between sets of SNVs, co-mutated in a clade-specific fashion. These so-called spots collect MOCs, mutations of concern, driving the fitness of the virus impacting its function preferentially of the spike-protein and its receptor binding domain but also of the nucleocapsid with immune-evasive consequences and also of other genes including the ORFs [35,39,52]. Mutational profiles of the different genes across the lineages provide a simple approach to estimate their impact. Most VOCs are affected by mutually independent SNV patterns, which evolved along different paths from the common root area, including the early virus-spread during the non-VOC period until autumn 2020. This region is evolutionarily uncertain in our map, meaning that it is not clear how near-identical sequences reflect developmental paths of the virus [11]. Predicting the future of the pandemic is uncertain; however, the genetic map visualizes the present situation which was described as follows: “If the original Wuhan variant is like a town, the virus has been taking local trains to explore the surrounding area, but it has not traveled to the next city—not yet.” [44].

SOM portrayal thus combines cartography of the overall mutational landscape of SARS-CoV-2 with a hierarchy of portraits ranging from the mean portraits of the different classes from GISAID, VOC/VOIs and Pangolin down to the ‘personalized’ portraits of the individual samples. Simple visual inspection enables to assign them to most of the VOCs/VOIs or to identify outliers owing to misclassifications or methodical problems such as insufficient sequencing depth. Finding unreliable data is one of the tasks involved in fixing the bioinformatics bottleneck in SARS-CoV-2 genome surveillance [11], e.g., owing to the rush of data sharing prior to sufficient quality control of sequence and metadata in some cases. In parallel to this publication, we provide an interactive tool to browse the presented data set more in detail (see data availability statement below). As a second methodical amendment, we introduced xSOM enabling extension of the existing SOM and demonstrated its performance using 36 virus genomes sequenced recently in Armenia by means of Nanopore technology [26].

A limitation of our study is its retro perspective character. The SOM is based on a data-freeze from April 2021. xSOM enables to consider new genomes from the continuously incoming flow of sequences obtained after the freezing date, given that the new sequence fits into the existing state space of the SARS-CoV-2 SNV. The presented SOM must be ‘renewed’ from time to time to account for novel evolutionary paths. A second limitation is the biased sequencing frequency in time and space. Sequencing frequency gained strongly in the VOC period since autumn 2020. Moreover, it changes strongly between countries from, e.g., more than 50% (of infected cases) in Iceland and Australia, about 5% in Great Britain, 2% in Germany and less than 0.5% in Russia and Brazil (https://www.covid19dataportal.it/highlights/highlight3/, in January 2021). This imbalance will bias the sequence space towards the more frequent variants. SOM partly levels this bias owing to its meta-SNV structure.

## 5. Conclusions

Despite some early hopes, the pandemic is not over. It further evolves, and new waves, driven by new mutations, will arise in time and space, possibly over years. Vaccination in combination with surveillance of the SARS-CoV-2 genome are key to holding the pandemic under control. Genetic control requires close-mashed sequencing combined with ‘bioinformatic surveillance’. Machine learning by means of SOM portrayal provides a novel option for this latter task, with strong odds regarding visualization, intuitive perception and ‘personalization’ of the mutational patterns of the virus genomes.

## Figures and Tables

**Figure A7:**
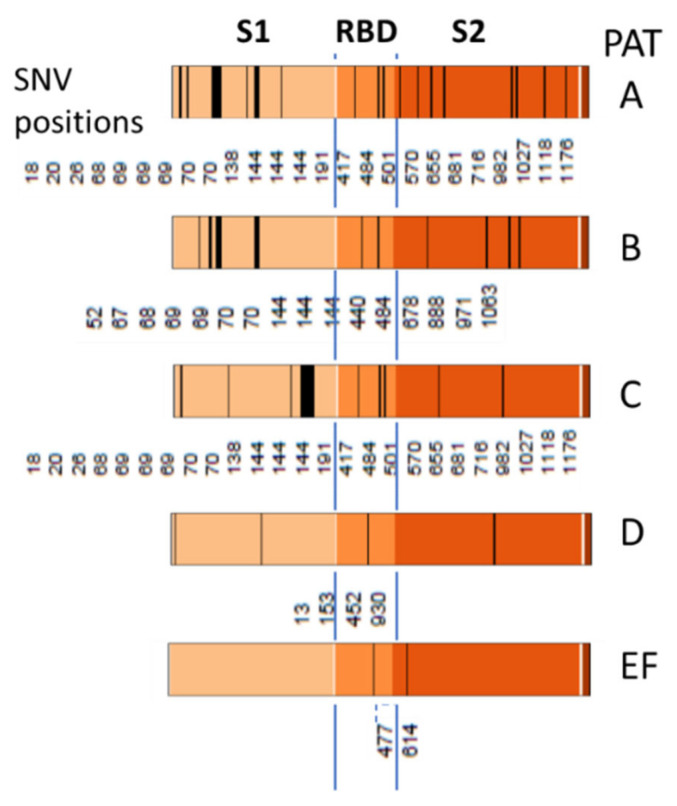
Mutations of the S-gene in the PAT’s. Position of the mutation is provided below the bars.

**Figure A9:**
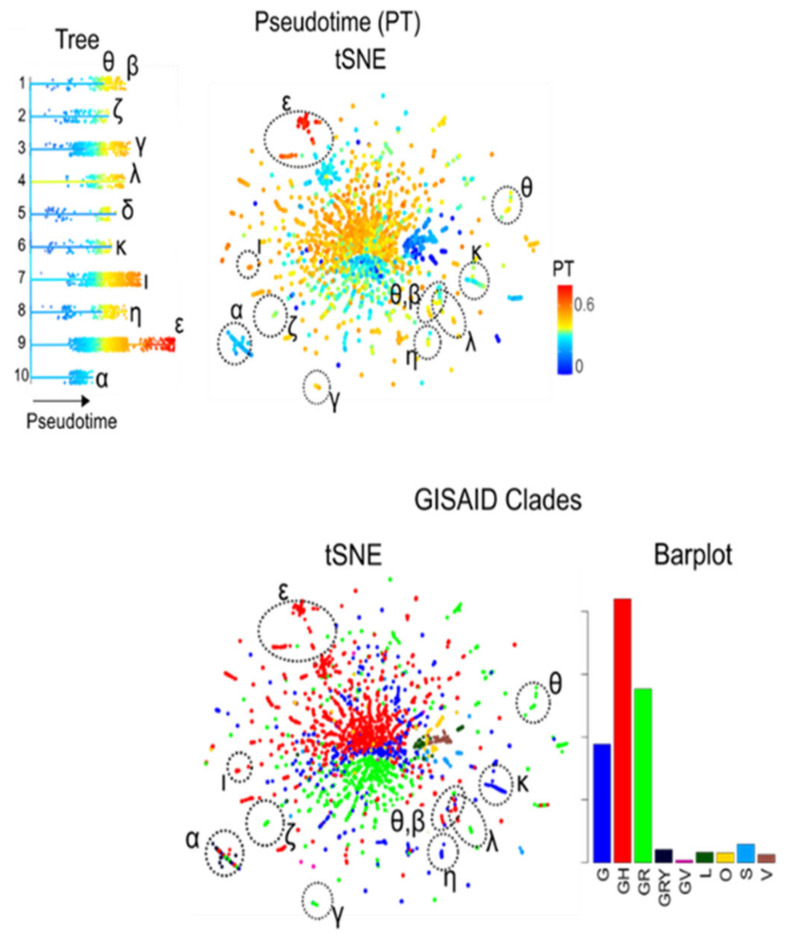
The URD-tree is colored in pseudotime (PT) scale. Most branches cover PT-ranges up to PT = 0.5–0.6. Shortest PT range is obtained for VOI alpha and the longest for VOI epsilon. The PT range seems to be governed by the number of intermediate virus genomes in between the initial and final states. t-SNE (t-distributed neighbor embedding) plots of the variants in PT and GISAID color scale reveal that clade GH (including VOI epsilon) covers the widest PT range.

## Data Availability

Details of the SARS-CoV-2 genome SOM portrayal can be interactively discovered using the oposSOM browser [27] available online via the IZBI web page (https://www.izbi.uni-leipzig.de/opossom-browser/ and https://apps.health-atlas.de/opossom-browser/?dataset=12). The browser enables selection and visualization of SNVs in the genome landscape and assessment of similarity relations between the variants and lineages together with their individual SOM portraits (Figure A11 and Figure A12).

## Supplementary Materials

The following material is available online at https://www.mdpi.com/article/10.3390/v13091764/s1. File S1: Gallery of SOM-portraits of SARS-CoV-2 genomes (File_S1.PDF); File S2: List of SNV (position in the SARS-CoV-2 genome, amino-acid substitution, spot).

## Appendix A. Additional Tables

**Table A1:** SARS-CoV-2 labeling schemes ^a^ and gallery of the respective SOM-portraits.

WHO ^a^	PANGO-LINE	GI-SAID	Next-Strain	PAT	Portrait Standard and Coastline Style
**Variants of Concern (VOCs)**					
alpha ‘British’ variant	B.1.1.1.7	GRY	20I	A	
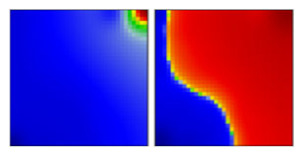
beta ‘South African’ variant	B.1.351	GH	20H	C	
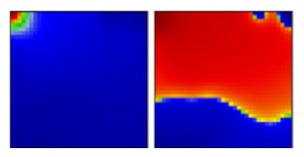
gamma ‘Brasilian’ variant	P1	GR	20J	A	
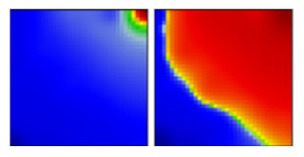
delta ^b^ ‘Indian’ variant	B1.617.2	G	21A	EF	
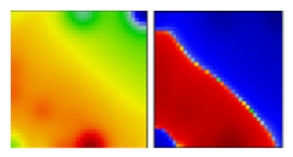
**Variants of Interest (VOIs)**					
lambda ^b^	C37	GR	21G	B	
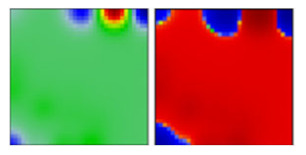
eta	B.1.525	G	21D	B	
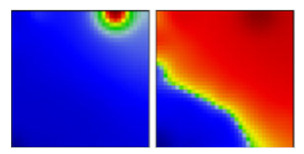
iota	B.1.526	GH	21F	EF	
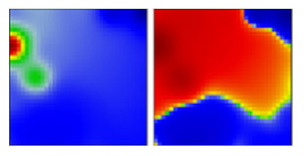
kappa	B.1.617.1	G	21B	EF	
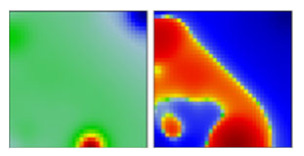
Others					
epsilon	B.1.427, B.1.429	GH	21C	D	
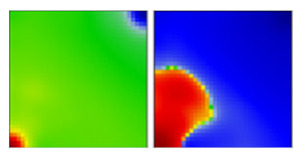
zeta ^b^	P2	GR	20B	EF	
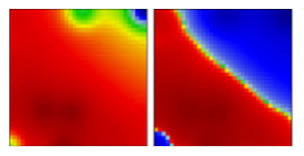
Theta ^b^	P3	GR	21E	EF	
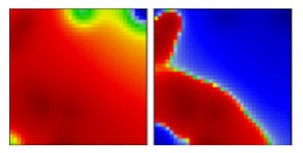
	A	S	19B	EF	
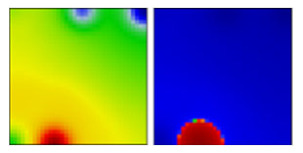
	A.1	S	19B	EF	
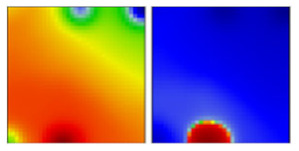
	B	L	19A	EF	
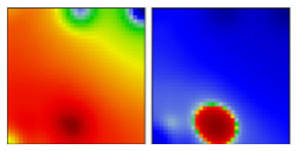
	B.1	G/GH	20A	EF	
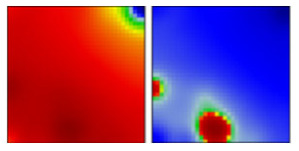
	B.1.1	GR	20B	EF	
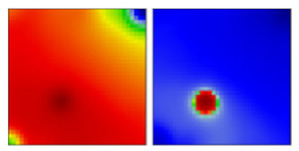
	B.1.1.136	GR	20D	EF	
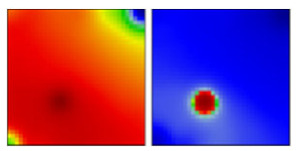
	B.1.1.186	GR	20D	EF	
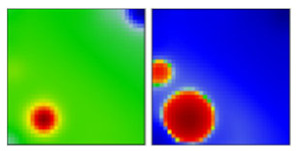
	B.1.1.205	GR	20B	EF	
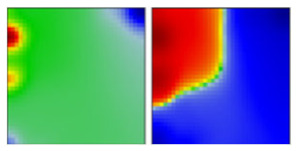
	B.1.1.228	GR	20B	EF	
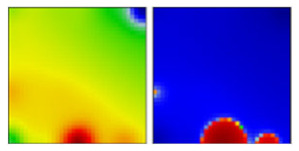
	B.1.1.231	GR	20B	EF	
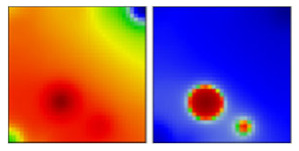
	B.1.1.316	GR	20B	EF	
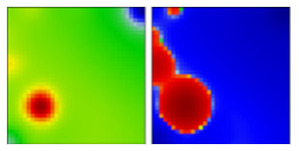
	B.1.1.434	GR	20B	EF	
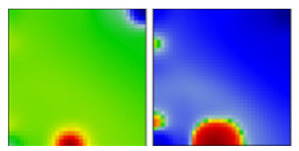
	B.1.1.519	GR	20B	EF	
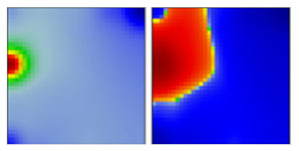
	B.1.110	GH	20A	EF	
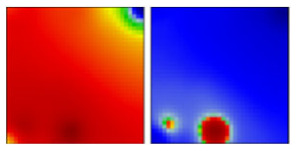
	B.1.139	G	20A	EF	
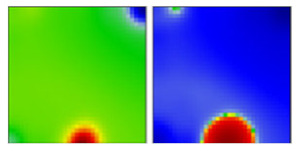
	B.1.2	GH	20C	EF	
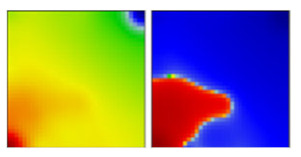
	B.1.234	G	20A	EF	
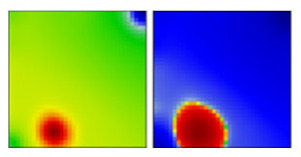
	B.1.274	GH	20A	EF	
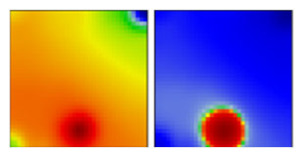
	B.1.298	GH	20A	EF	
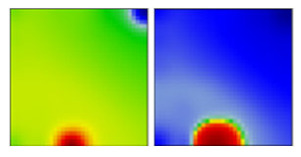
	B.1.305	GH	20C	EF	
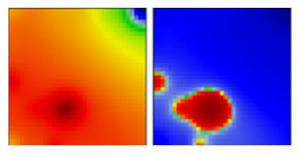
	B.1.360	GH	20C	EF	
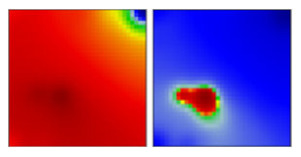
	B.1.400	G	-	EF	
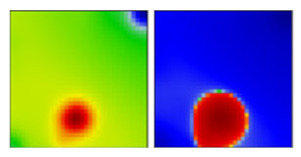
	B.1.517	GH	-	EF	
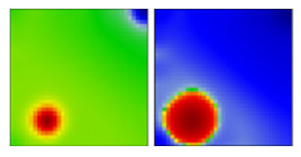
	B.1.595	GH	-	D/EF	
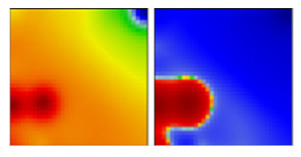
	B.19	L	19A	EF	
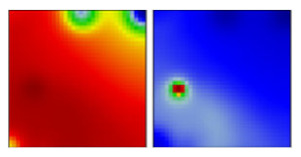
	B.46	L	19A	EF	
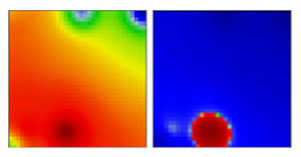
	C.26	GR	20D	EF	
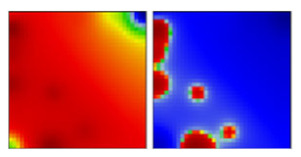
	C.35	GR		EF	
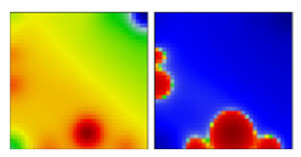
	D.2	GR		EF	
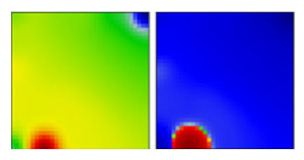
	W.1	GV		EF	
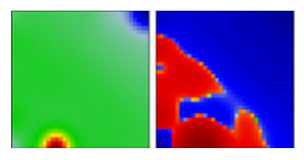

^a^ Adapted from https://www.who.int/en/activities/tracking-SARS-CoV-2-variants/ (10 July 2021); for PANGOLIN see https://cov-lineages.org/lineage_list.html (on 17 July 2021); ^b^ xSOM added.

**Table A2:** Spot characteristics.

Spot	Enriched Lineages	Number of SNVs	SNV in the Spot ^a^
A	Alpha, gamma	72	Orf1ab: 733, 913, 2110, 2749, 3267, 3828, 5388, 5648, 5986, 6319, 6613, 6954, 11288, 11289, 11290, 11291, 11292, 11293, 11294, 11295, 11296, 12778, 13860, 14120, 14676, 15279, 16176, 17259, 17615 S: 21614, 21621, 21638, 21765, 21766, 21767, 21768, 21769, 21770, 21974, 21991, 21992, 21993, 22132, 22812, 23012, 23063, 23271, 23525, 23604, 23709, 24506, 24642, 24914, 25088 Orf3a: 26149 Orf8: 27972 28048 28095 28111 28167 N: 28280 28281 28282 28512 28877 28878 28881 28882 28883 28977 Intergenic: 28271, 29834
B	eta	37	Orf1ab: 1498, 1594, 1807, 2659, 5869, 6285, 8031, 8323, 8593, 9565, 12540, 14407, 18171, 18646, 19684, 20724 S: 21717, 21762, 21764, 22879, 23593, 24224, 24472, 24748 Orf3a:25613 E: 26305 M: 26767 Orf6: 27205, 27206, 27207 Orf7a: 27425 N: 28278, 28279, 28308, 28699 Intergenic: 12, 29543
C	beta	33	Orf1ab: 661, 2692, 2830, 3966, 5100, 5230, 8043, 10323, 13620, 17999, 18525, 19524 S: 21801, 22206, 22281, 22282, 22283, 22284, 22285, 22286, 22287, 22288, 22289, 22813, 23664, 24415 Orf3a: 25904, 26158 E: 26456 Orf8: 28253 Intergenic: 174, 29743, 29754
D	epsilon	46	Orf1ab: 1059, 2395, 2597, 3817, 8083, 8257, 8895, 8947, 9738, 9991, 10319 10641, 10831, 12100, 12878, 13019, 13713, 14805, 16394, 17014, 18424, 19515, 21304 S: 21600, 22018, 22335, 22597, 22917, 23126, 23155, 24349 Orf3a: 25563, 25907 M: 26681 Orf8: 27964, 27987, 28087, 28191 N: 28472, 28869, 28887, 28975, 29362, 29402 Intergenic: 27890, 28272
E	kappa	207	Orf1ab: 445, 490, 1157, 1163, 1578, 1624, 1812, 2227, 2244, 2258, 2488, 2937, 2973, 3114, 3177, 3355, 3564, 3768, 3896, 3951, 3952, 3953, 3984, 4002, 4158, 4303, 5140, 5144, 5974, 6033, 6070, 6317, 6320, 6403, 6441, 6502, 6543, 6606, 6618, 7113, 7540, 7819, 7833, 7945, 7960, 8140, 8149, 8662, 8782, 9204, 9430, 9805, 9875, 9996, 10078, 10332, 10456, 10717, 10741, 11008, 11077, 11453, 11575, 11830, 11866, 12116, 13059, 13094, 13216, 13354, 14187, 14241, 14316, 14808, 14980, 15102, 15327, 15594, 16647, 16728, 17140, 17463, 17642, 17676, 17747, 17858, 18060, 18543, 18555, 18568, 18736, 18981, 19072, 19215, 19422, 19735, 19816, 19983, 20016, 20091, 20268, 20437, 20629, 21077, 21099, 21255, 21390, 21516 S: 21622, 21644, 21773, 21844, 21850, 21986, 22101, 22227, 22326, 22480, 22591, 22852, 22992, 23120, 23401, 23457, 23577, 23608, 23624, 24026, 24034, 24076, 24337, 24370, 24727, 24766, 24771, 24852, 25266 Orf3a: 25459, 25514, 25515, 25710, 25714, 25757, 25785, 25793, 25922, 26072, 26162 E: 26326 M: 26607, 26669, 26690, 26729, 26801, 26882, 27024, 27059, 27110 Orf6: 27213, 27281 Orf7a: 27483, 27579, 27600, 27635, 27679 Orf7b: 27812 Orf8: 27923, 27944, 27957, 28077, 28144 N: 28520, 28657, 28690, 28774, 28854, 28880, 28884, 28885, 28886, 28888, 28889, 28891, 28894, 28896, 28932, 28961, 29095, 29266, 29384, 29412, 29445, 29527 Orf10: 29645 Intergenic: 13, 19, 80, 173, 180, 201, 205, 221, 29546, 29692, 29700, 29710, 29803
F	iota	111	Orf1ab: 565, 686, 687, 688, 689, 690, 691, 692, 693, 694, 1132, 2644, 2683, 2867, 2945, 3140, 3745, 4456, 6015, 6101, 6379, 6479, 6751, 7201, 8809, 8890, 9152, 9190, 9289, 9654, 9867, 10029, 10567, 10705, 10775, 10954, 11117, 11203, 11653, 12043, 12789, 14210, 16396, 16500, 16569, 16859, 17748, 18452, 18647, 19068, 19839, 20262, 20592, 21306 S: 21575, 21642, 21846, 22320, 22957, 22995, 23047, 23248, 23695, 23731, 23756, 24095, 24432, 24799, 24933, 25340 Orf3a: 25517, 25587, 25844, 25948, 25968 M: 26700 Orf7a: 27534, 27630, 27739 Orf8: 27925 N: 28311, 28531, 28706, 28879, 29197, 29311 Orf10: 29566 Intergenic: 140, 203, 222, 29738, 29739, 29740, 29741, 29742, 29744, 29745, 29746, 29747, 29748, 29749, 29750, 29751, 29752, 29753, 29755, 29756, 29757, 29758, 29759, 29760

^a^ SNVs were given as sequence position along the SARS-CoV-2 genome. They include deletions, insertions and substitutions. The respective nucleotides, amino acids and amino acid positions are provided in Supplementary Materials: File S2).
