# Supplementary material for: The Evolving Faces of the SARS-CoV-2 Genome
Source: Viruses. 2021 Sep 3;13(9):1764. doi: 10.3390/v13091764 (PMC8472651; doi:10.3390/v13091764)
Supplement: Supplementary file 1 [file viruses-13-01764-s001.zip › FileS1_Gallery_of_Expression_Portrats_in_Pangolin.pdf]

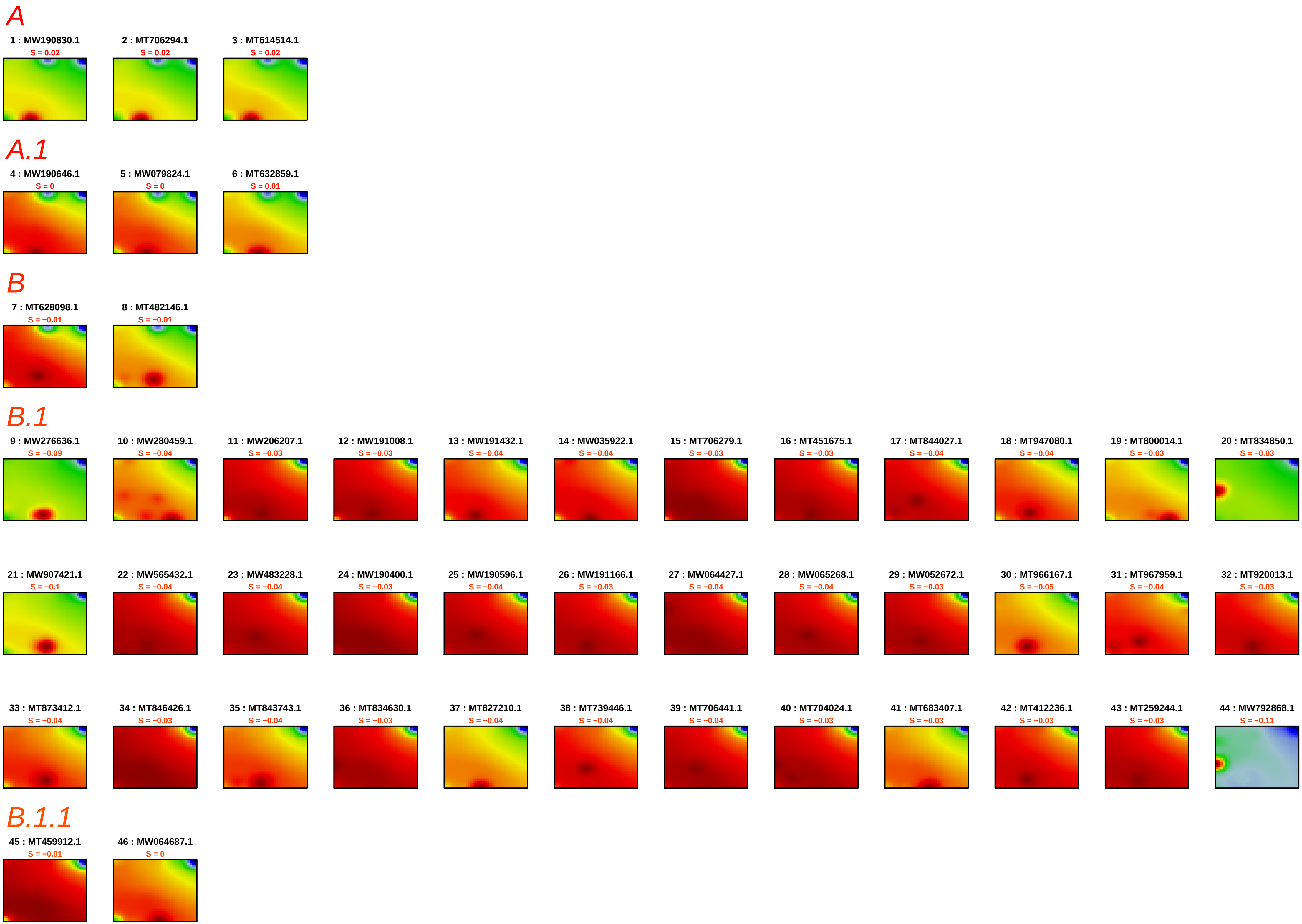
A
1 : MW190830.1
S = 0.02
2 : MT706294.1
S = 0.02
3 : MT614514.1
S = 0.02
A.1
4 : MW190646.1
S = 0
5 : MW079824.1
S = 0
6 : MT632859.1
S = 0.01
B
7 : MT628098.1
S = −0.01
8 : MT482146.1
S = −0.01
B.1
9 : MW276636.1
S = −0.09
10 : MW280459.1
S = −0.04
11 : MW206207.1
S = −0.03
12 : MW191008.1
S = −0.03
13 : MW191432.1
S = −0.04
14 : MW035922.1
S = −0.04
15 : MT706279.1
S = −0.03
16 : MT451675.1
S = −0.03
17 : MT844027.1
S = −0.04
18 : MT947080.1
S = −0.04
19 : MT800014.1
S = −0.03
20 : MT834850.1
S = −0.03
21 : MW907421.1
S = −0.1
22 : MW565432.1
S = −0.04
23 : MW483228.1
S = −0.04
24 : MW190400.1
S = −0.03
25 : MW190596.1
S = −0.04
26 : MW191166.1
S = −0.03
27 : MW064427.1
S = −0.04
28 : MW065268.1
S = −0.04
29 : MW052672.1
S = −0.03
30 : MT966167.1
S = −0.05
31 : MT967959.1
S = −0.04
32 : MT920013.1
S = −0.03
33 : MT873412.1
S = −0.04
34 : MT846426.1
S = −0.03
35 : MT843743.1
S = −0.04
36 : MT834630.1
S = −0.03
37 : MT827210.1
S = −0.04
38 : MT739446.1
S = −0.04
39 : MT706441.1
S = −0.04
40 : MT704024.1
S = −0.03
41 : MT683407.1
S = −0.03
42 : MT412236.1
S = −0.03
43 : MT259244.1
S = −0.03
44 : MW792868.1
S = −0.11
B.1.1
45 : MT459912.1
S = −0.01
46 : MW064687.1
S = 0

B.1.1.136

47 : MT972137.1

S = 0

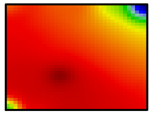

B.1.1.169

48 : MW279423.1

S = 0

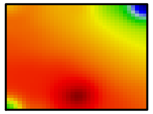

B.1.1.186

49 : MW719920.1

S = 0

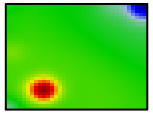

B.1.1.205

50 : MW306370.1

S = 0

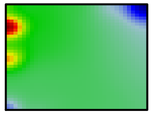

B.1.1.222

51 : MW681161.1

S = 0

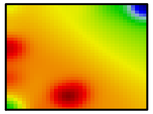

52 : MW653612.1

S = 0

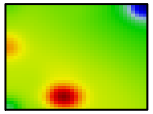

B.1.1.228

53 : MW645720.1

S = −0.01

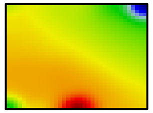

54 : MW206329.1

S = 0.02

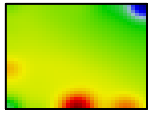

B.1.1.231

55 : MW521681.1

S = 0

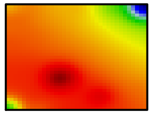

B.1.1.244

56 : MW206601.1
S = 0

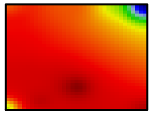

B.1.1.291

57 : MT831212.1
S = −0.01

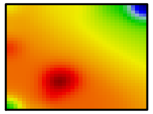

58 : MT831708.1
S = −0.01

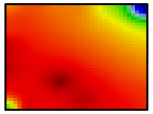

B.1.1.316

59 : MW864396.1
S = 0.12

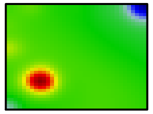

60 : MW666340.1
S = 0.09

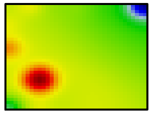

B.1.1.416

61 : MW738279.1
S = 0

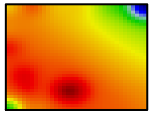

B.1.1.432

62 : MW763480.1
S = 0.01

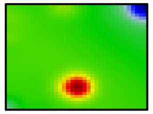

63 : MW521794.1
S = −0.02

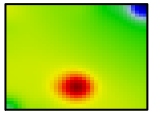

B.1.1.434

64 : MW668844.1
S = 0

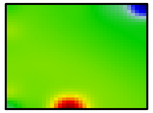

B.1.1.519

65 : MW903995.1
S = 0.25

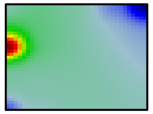

66 : MW849227.1
S = 0.41

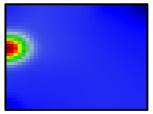

67 : MW849880.1
S = 0.35

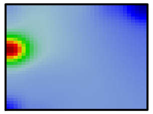

68 : MW681413.1
S = 0.28

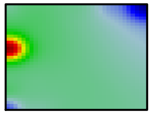

69 : MW667310.1
S = 0.3

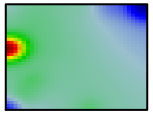

B.1.1.7 _VOC

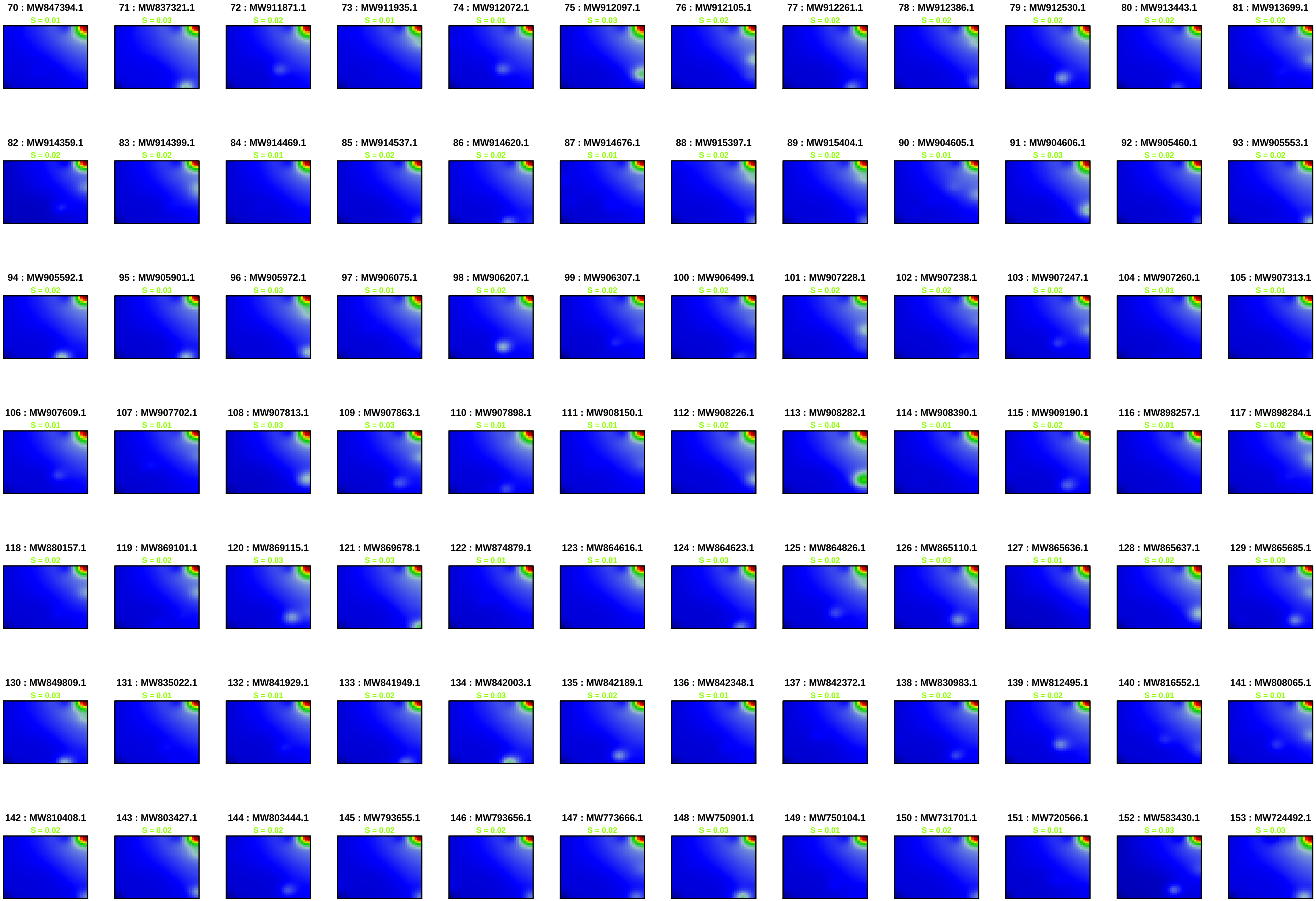
70 : MW847394.1
S = 0.01
71 : MW837321.1
S = 0.03
72 : MW911871.1
S = 0.02
73 : MW911935.1
S = 0.01
74 : MW912072.1
S = 0.01
75 : MW912097.1
S = 0.03
76 : MW912105.1
S = 0.02
77 : MW912261.1
S = 0.02
78 : MW912386.1
S = 0.02
79 : MW912530.1
S = 0.02
80 : MW913443.1
S = 0.02
81 : MW913699.1
S = 0.02
82 : MW914359.1
S = 0.02
83 : MW914399.1
S = 0.02
84 : MW914469.1
S = 0.01
85 : MW914537.1
S = 0.02
86 : MW914620.1
S = 0.02
87 : MW914676.1
S = 0.01
88 : MW915397.1
S = 0.02
89 : MW915404.1
S = 0.02
90 : MW904605.1
S = 0.01
91 : MW904606.1
S = 0.03
92 : MW905460.1
S = 0.02
93 : MW905553.1
S = 0.02
94 : MW905592.1
S = 0.02
95 : MW905901.1
S = 0.03
96 : MW905972.1
S = 0.03
97 : MW906075.1
S = 0.01
98 : MW906207.1
S = 0.02
99 : MW906307.1
S = 0.02
100 : MW906499.1
S = 0.02
101 : MW907228.1
S = 0.02
102 : MW907238.1
S = 0.02
103 : MW907247.1
S = 0.02
104 : MW907260.1
S = 0.01
105 : MW907313.1
S = 0.01
106 : MW907609.1
S = 0.01
107 : MW907702.1
S = 0.01
108 : MW907813.1
S = 0.03
109 : MW907863.1
S = 0.03
110 : MW907898.1
S = 0.01
111 : MW908150.1
S = 0.01
112 : MW908226.1
S = 0.02
113 : MW908282.1
S = 0.04
114 : MW908390.1
S = 0.01
115 : MW909190.1
S = 0.02
116 : MW898257.1
S = 0.01
117 : MW898284.1
S = 0.02
118 : MW880157.1
S = 0.02
119 : MW869101.1
S = 0.02
120 : MW869115.1
S = 0.03
121 : MW869678.1
S = 0.03
122 : MW874879.1
S = 0.01
123 : MW864616.1
S = 0.01
124 : MW864623.1
S = 0.03
125 : MW864826.1
S = 0.02
126 : MW865110.1
S = 0.03
127 : MW865636.1
S = 0.01
128 : MW865637.1
S = 0.02
129 : MW865685.1
S = 0.03
130 : MW849809.1
S = 0.03
131 : MW835022.1
S = 0.01
132 : MW841929.1
S = 0.01
133 : MW841949.1
S = 0.02
134 : MW842003.1
S = 0.03
135 : MW842189.1
S = 0.02
136 : MW842348.1
S = 0.01
137 : MW842372.1
S = 0.01
138 : MW830983.1
S = 0.02
139 : MW812495.1
S = 0.02
140 : MW816552.1
S = 0.01
141 : MW808065.1
S = 0.01
142 : MW810408.1
S = 0.02
143 : MW803427.1
S = 0.02
144 : MW803444.1
S = 0.02
145 : MW793655.1
S = 0.02
146 : MW793656.1
S = 0.02
147 : MW773666.1
S = 0.02
148 : MW750901.1
S = 0.03
149 : MW750104.1
S = 0.01
150 : MW731701.1
S = 0.02
151 : MW720566.1
S = 0.01
152 : MW583430.1
S = 0.03
153 : MW724492.1
S = 0.03

B.1.110

154 : MW364999.1
S = 0

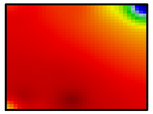

B.1.110.3

155 : MW825510.1
S = 0.17

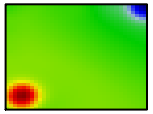

156 : MW813533.1
S = 0.16

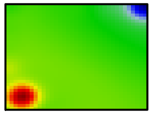

157 : MW772211.1
S = 0.18

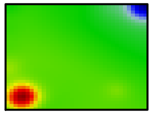

B.1.112

158 : MW206178.1
S = 0

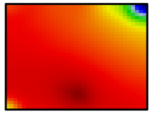

B.1.139

159 : MT750131.1
S = 0

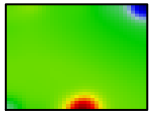

B.1.181

160 : MW079838.1
S = 0

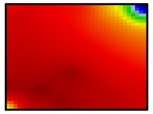

B.1.2

161 : MW903075.1
S = −0.04

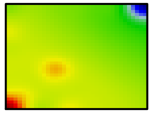

162 : MW898282.1
S = −0.02

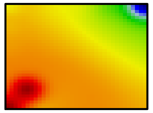

163 : MW891025.1
S = −0.07

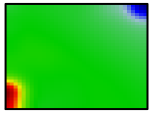

164 : MW892093.1
S = −0.09

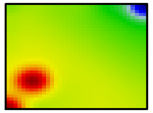

165 : MW897491.1
S = −0.02

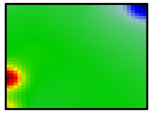

166 : MW888010.1
S = −0.01

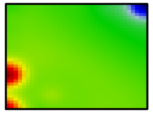

167 : MW871122.1
S = −0.04

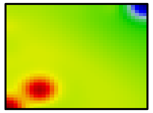

168 : MW865415.1
S = −0.03

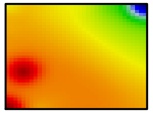

169 : MW865437.1
S = −0.04

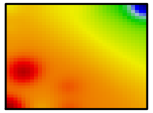

170 : MW849046.1
S = −0.01

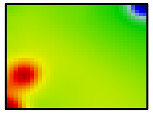

171 : MW849140.1
S = −0.02

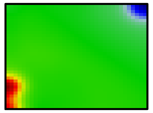

172 : MW850290.1
S = −0.03

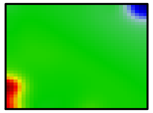

173 : MW837489.1
S = −0.03

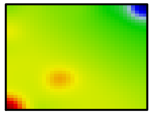

174 : MW813248.1
S = −0.02

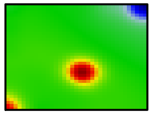

175 : MW816195.1
S = −0.13

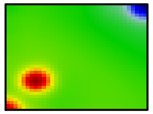

176 : MW804890.1
S = −0.04

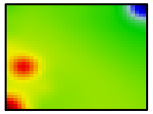

177 : MW782913.1
S = −0.04

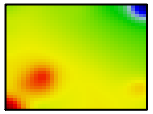

178 : MW782957.1
S = −0.02

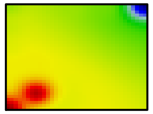

179 : MW773379.1
S = −0.04

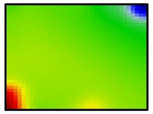

180 : MW766758.1
S = −0.03

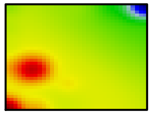

181 : MW739293.1
S = −0.03

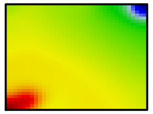

182 : MW739740.1
S = −0.01

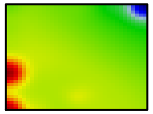

183 : MW731110.1
S = −0.04

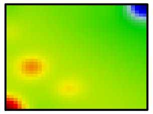

184 : MW731198.1
S = −0.03

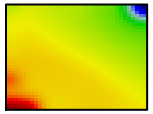

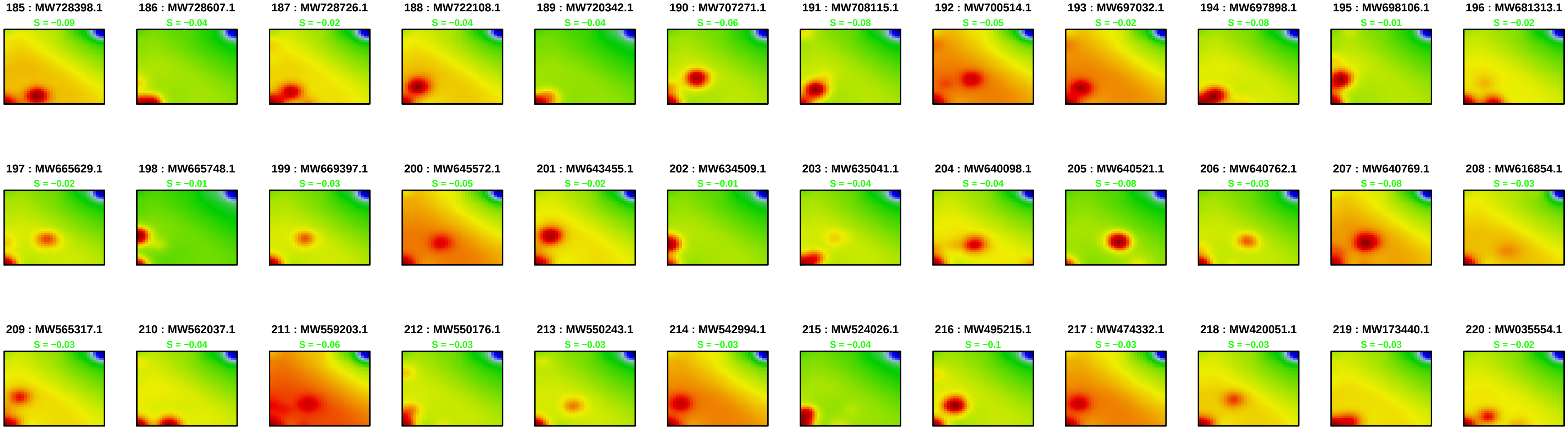

B.1.232

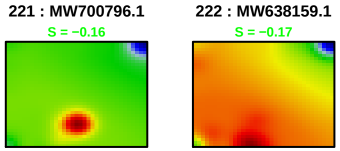

B.1.234

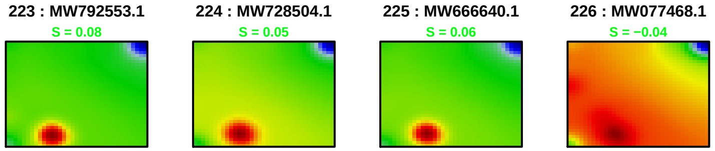

B.1.240

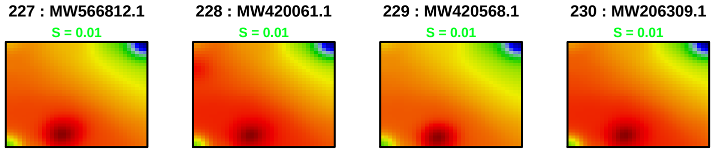

B.1.243

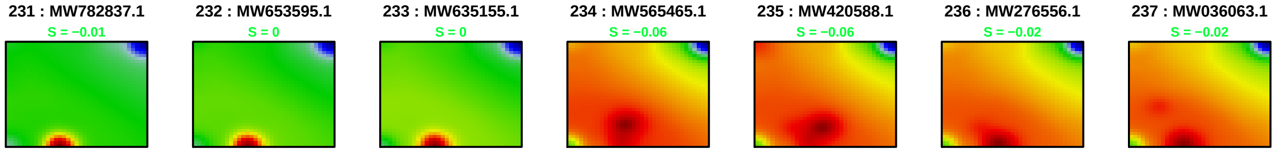

B.1.258.21

238 : MW453084.1

S = 0

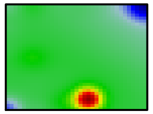

B.1.265

239 : MW708707.1

S = 0

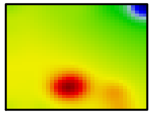

B.1.274

240 : MW206082.1

S = 0

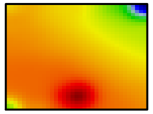

B.1.284

241 : MW064341.1

S = 0

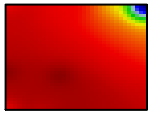

B.1.298

242 : MW709154.1

S = 0

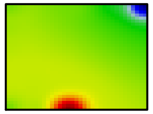

B.1.305

243 : MW815792.1

S = 0

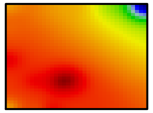

B.1.320

244 : MT641523.1

S = 0.01

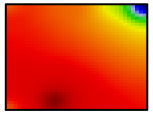

245 : MT628158.1

S = 0.01

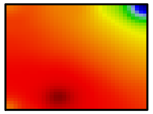

B.1.349

246 : MW668829.1
S = 0

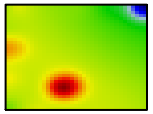

B.1.351 _VOC

247 : MW621453.1
S = 0.56

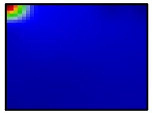

248 : MW913437.1
S = 0.48

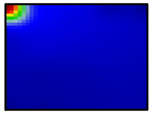

249 : MW914008.1
S = 0.56

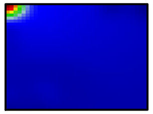

250 : MW914015.1
S = 0.56

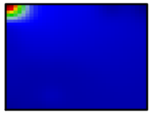

251 : MW914542.1
S = 0.32

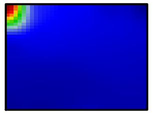

252 : MW905844.1
S = 0.42

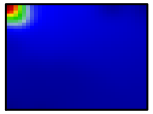

253 : MW907199.1
S = 0.43

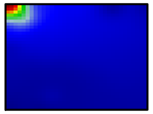

254 : MW907323.1
S = 0.55

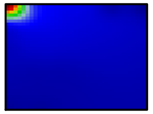

255 : MW908243.1
S = 0.54

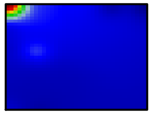

256 : MW908815.1
S = 0.56

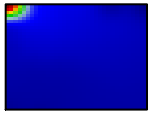

257 : MW909222.1
S = 0.57

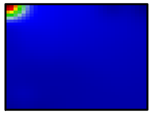

258 : MW849825.1
S = 0.51

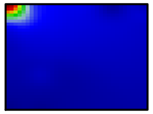

259 : MW795351.1
S = 0.49

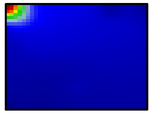

260 : MW796654.1
S = 0.53

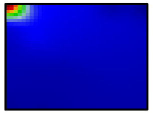

261 : MW792684.1
S = 0.54

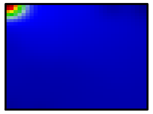

262 : MW792756.1
S = 0.41

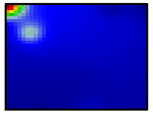

263 : MW793005.1
S = 0.52

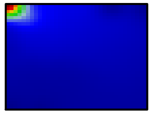

264 : MW773815.1
S = 0.55

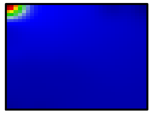

B.1.36.27

265 : MW691153.1
S = 0

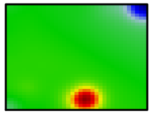

B.1.360

266 : MW064652.1
S = 0

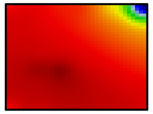

B.1.369

267 : MT843300.1
S = −0.02

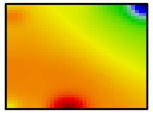

268 : MT827251.1
S = −0.03

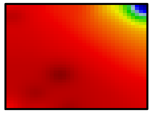

B.1.371

269 : MT994395.1
S = 0

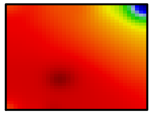

270 : MT642377.1
S = 0

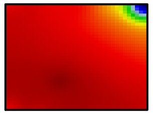

B.1.382

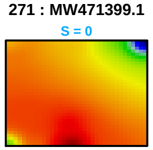

B.1.384

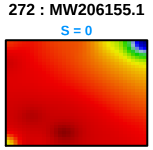

B.1.400

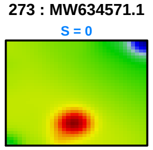

B.1.427 _VOC

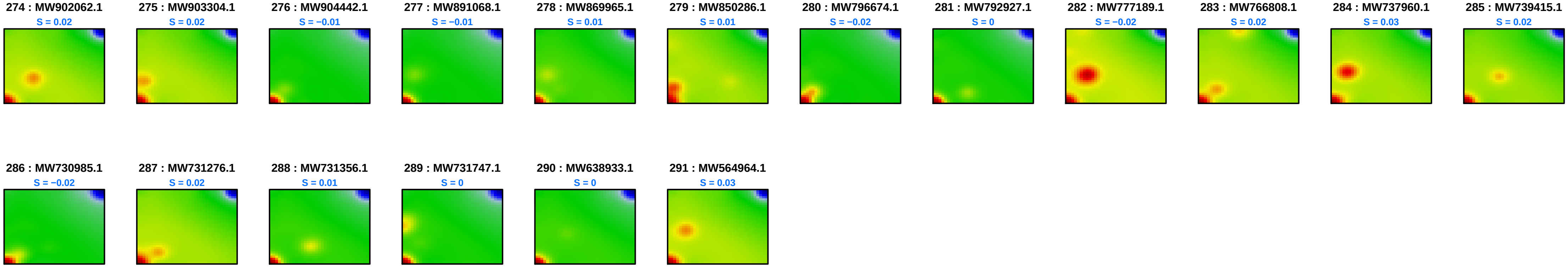

B.1.429 _VOC

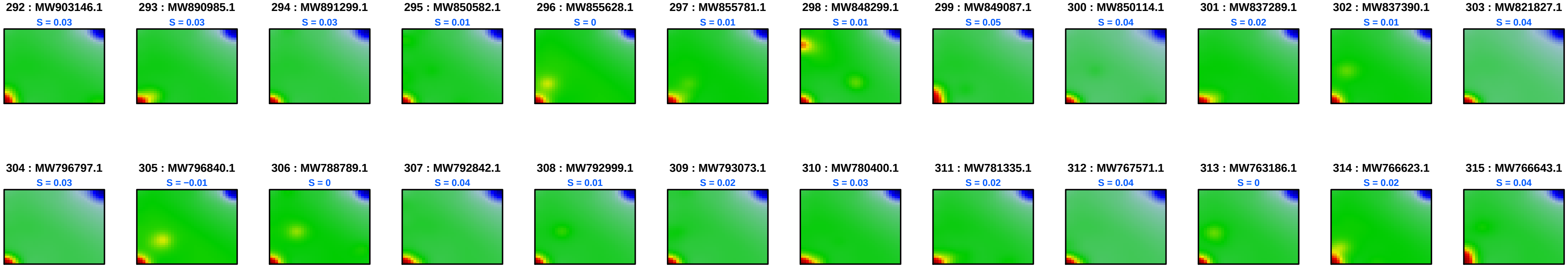

316 : MW766742.1
S = 0.01
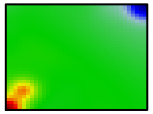

317 : MW739122.1
S = −0.01
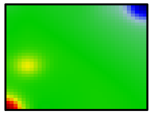

318 : MW731201.1
S = 0.01
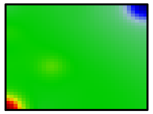

319 : MW731327.1
S = 0.01
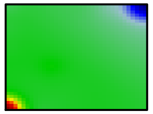

320 : MW731376.1
S = 0.01
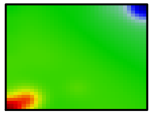

321 : MW731779.1
S = 0.03
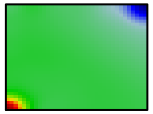

322 : MW734228.1
S = −0.01
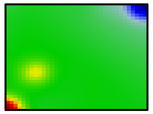

323 : MW734357.1
S = 0.01
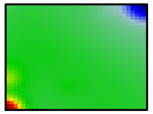

324 : MW708099.1
S = 0.02
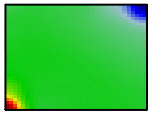

325 : MW708538.1
S = 0.02
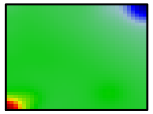

326 : MW701159.1
S = 0.02
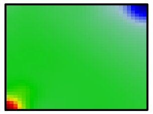

327 : MW701205.1
S = 0.04
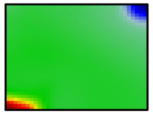

328 : MW702079.1
S = 0.03
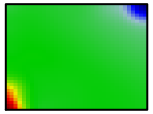

329 : MW702856.1
S = 0
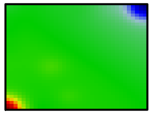

330 : MW703006.1
S = 0.02
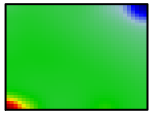

331 : MW692964.1
S = 0.04
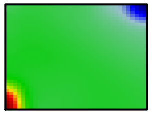

332 : MW545329.1
S = 0.04
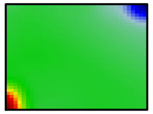

333 : MW460632.1
S = 0.01
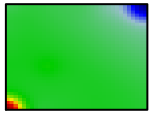

B.1.436

334 : MW279421.1
S = 0
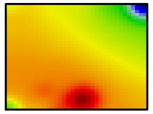

B.1.444

335 : MW420740.1
S = 0
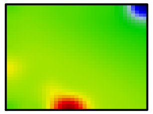

B.1.446

336 : MT358739.1
S = 0
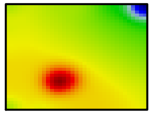

B.1.492

337 : MW645969.1
S = 0
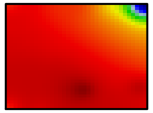

B.1.517

338 : MW782901.1
S = 0
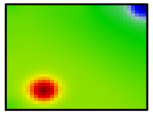

339 : MW783000.1
S = −0.01
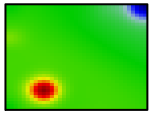

340 : MW715189.1
S = 0
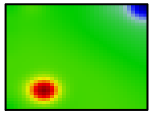

341 : MW550003.1
S = 0.01
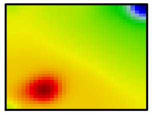

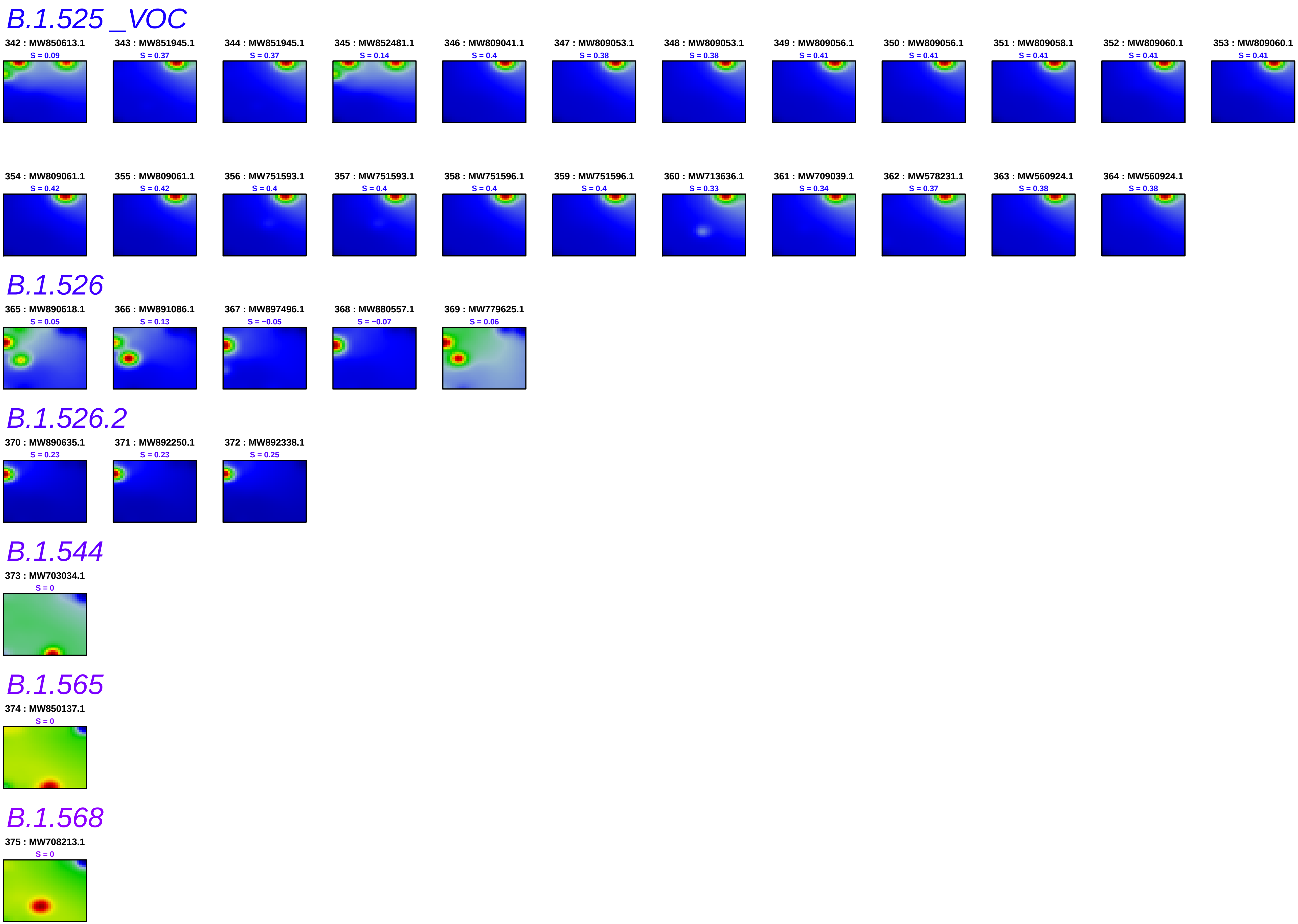

B.1.525_VOC
342 : MW850613.1
S = 0.09
343 : MW851945.1
S = 0.37
344 : MW851945.1
S = 0.37
345 : MW852481.1
S = 0.14
346 : MW809041.1
S = 0.4
347 : MW809053.1
S = 0.38
348 : MW809053.1
S = 0.38
349 : MW809056.1
S = 0.41
350 : MW809056.1
S = 0.41
351 : MW809058.1
S = 0.41
352 : MW809060.1
S = 0.41
353 : MW809060.1
S = 0.41
354 : MW809061.1
S = 0.42
355 : MW809061.1
S = 0.42
356 : MW751593.1
S = 0.4
357 : MW751593.1
S = 0.4
358 : MW751596.1
S = 0.4
359 : MW751596.1
S = 0.4
360 : MW713636.1
S = 0.33
361 : MW709039.1
S = 0.34
362 : MW578231.1
S = 0.37
363 : MW560924.1
S = 0.38
364 : MW560924.1
S = 0.38
B.1.526
365 : MW890618.1
S = 0.05
366 : MW891086.1
S = 0.13
367 : MW897496.1
S = −0.05
368 : MW880557.1
S = −0.07
369 : MW779625.1
S = 0.06
B.1.526.2
370 : MW890635.1
S = 0.23
371 : MW892250.1
S = 0.23
372 : MW892338.1
S = 0.25
B.1.544
373 : MW703034.1
S = 0
B.1.565
374 : MW850137.1
S = 0
B.1.568
375 : MW708213.1
S = 0

B.1.575

376 : MW902922.1

S = 0

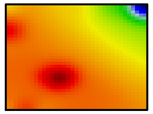

B.1.590

377 : MW191464.1

S = 0

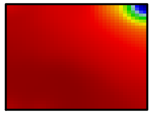

B.1.595

378 : MW735956.1

S = −0.15

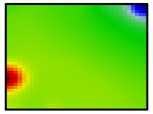

379 : MW728639.1

S = −0.2

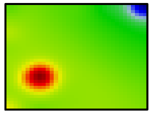

B.1.595.1

380 : MW286639.1

S = 0

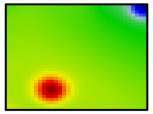

B.1.596

381 : MW904425.1

S = 0.03

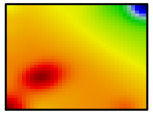

382 : MW891324.1

S = 0.05

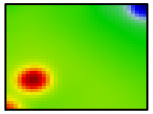

383 : MW820254.1

S = 0.02

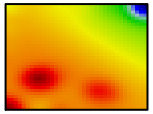

B.1.617.1 _VOC

384 : MW903294.1

S = 0

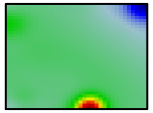

B.19

385 : MT438750.1

S = 0

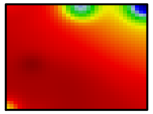

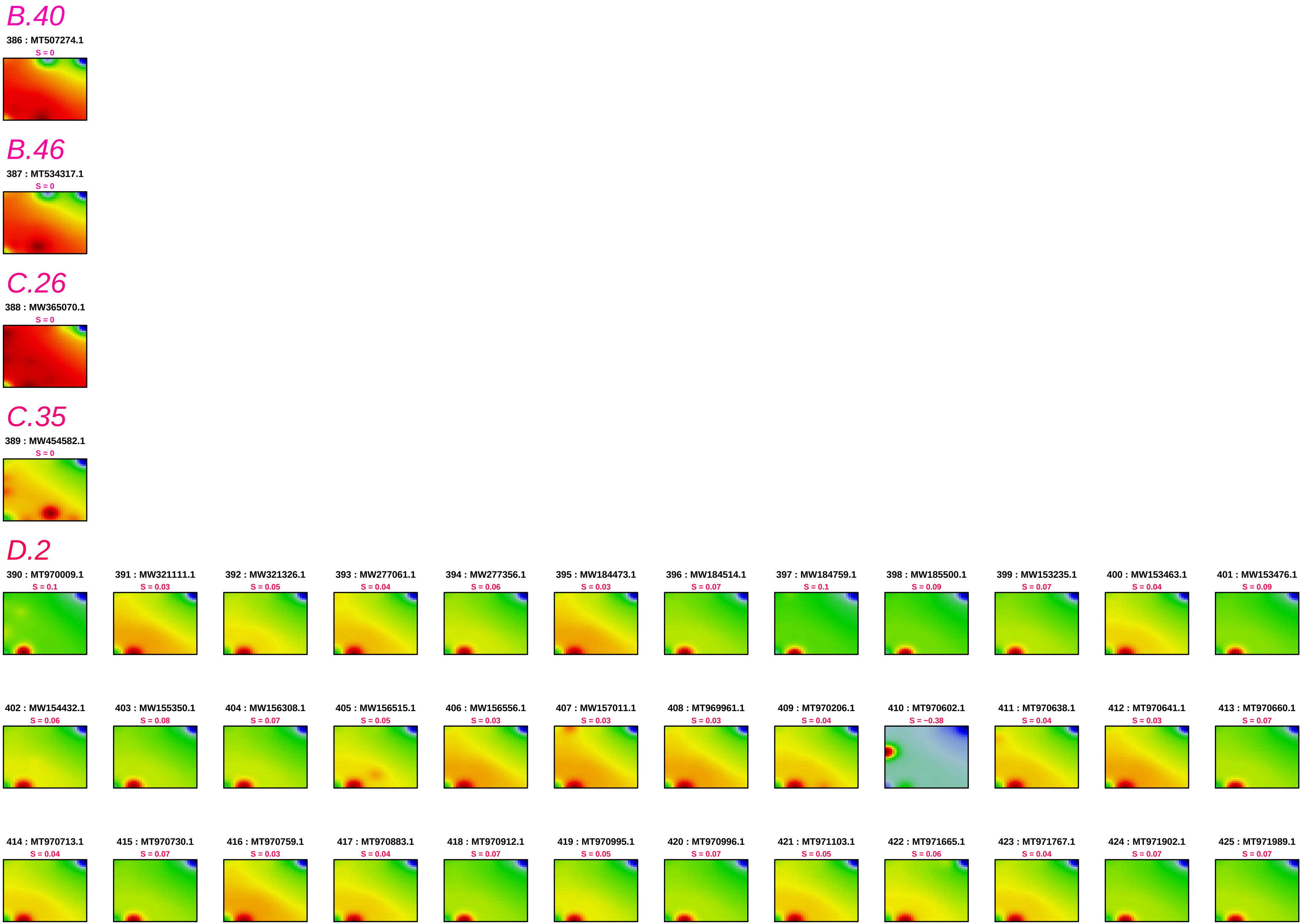

B.40
386 : MT507274.1
S = 0
B.46
387 : MT534317.1
S = 0
C.26
388 : MW365070.1
S = 0
C.35
389 : MW454582.1
S = 0
D.2
390 : MT970009.1
S = 0.1
391 : MW321111.1
S = 0.03
392 : MW321326.1
S = 0.05
393 : MW277061.1
S = 0.04
394 : MW277356.1
S = 0.06
395 : MW184473.1
S = 0.03
396 : MW184514.1
S = 0.07
397 : MW184759.1
S = 0.1
398 : MW185500.1
S = 0.09
399 : MW153235.1
S = 0.07
400 : MW153463.1
S = 0.04
401 : MW153476.1
S = 0.09
402 : MW154432.1
S = 0.06
403 : MW155350.1
S = 0.08
404 : MW156308.1
S = 0.07
405 : MW156515.1
S = 0.05
406 : MW156556.1
S = 0.03
407 : MW157011.1
S = 0.03
408 : MT969961.1
S = 0.03
409 : MT970206.1
S = 0.04
410 : MT970602.1
S = −0.38
411 : MT970638.1
S = 0.04
412 : MT970641.1
S = 0.03
413 : MT970660.1
S = 0.07
414 : MT970713.1
S = 0.04
415 : MT970730.1
S = 0.07
416 : MT970759.1
S = 0.03
417 : MT970883.1
S = 0.04
418 : MT970912.1
S = 0.07
419 : MT970995.1
S = 0.05
420 : MT970996.1
S = 0.07
421 : MT971103.1
S = 0.05
422 : MT971665.1
S = 0.06
423 : MT971767.1
S = 0.04
424 : MT971902.1
S = 0.07
425 : MT971989.1
S = 0.07

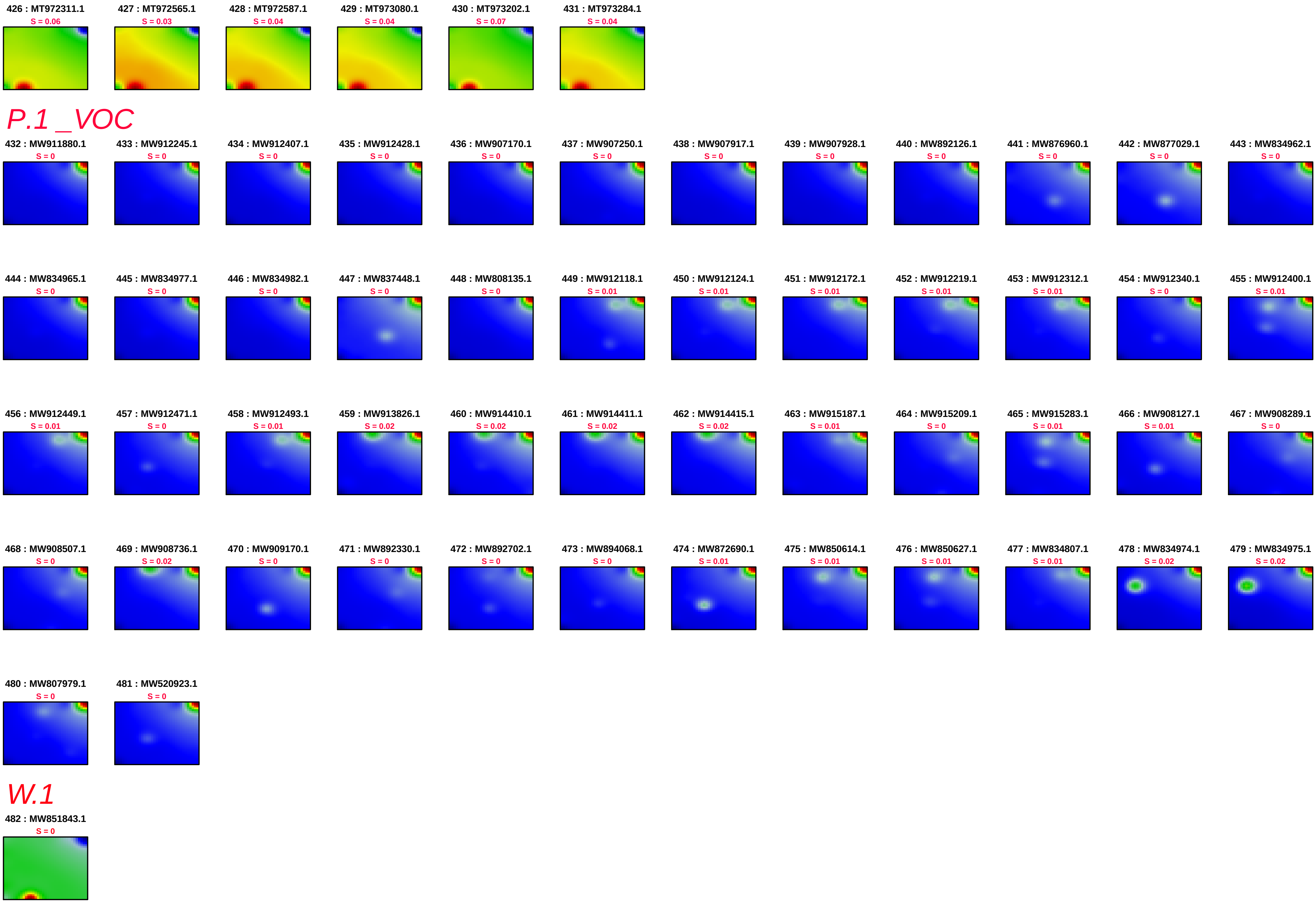
426 : MT972311.1
S = 0.06
427 : MT972565.1
S = 0.03
428 : MT972587.1
S = 0.04
429 : MT973080.1
S = 0.04
430 : MT973202.1
S = 0.07
431 : MT973284.1
S = 0.04
P.1 _VOC
432 : MW911880.1
S = 0
433 : MW912245.1
S = 0
434 : MW912407.1
S = 0
435 : MW912428.1
S = 0
436 : MW907170.1
S = 0
437 : MW907250.1
S = 0
438 : MW907917.1
S = 0
439 : MW907928.1
S = 0
440 : MW892126.1
S = 0
441 : MW876960.1
S = 0
442 : MW877029.1
S = 0
443 : MW834962.1
S = 0
444 : MW834965.1
S = 0
445 : MW834977.1
S = 0
446 : MW834982.1
S = 0
447 : MW837448.1
S = 0
448 : MW808135.1
S = 0
449 : MW912118.1
S = 0.01
450 : MW912124.1
S = 0.01
451 : MW912172.1
S = 0.01
452 : MW912219.1
S = 0.01
453 : MW912312.1
S = 0.01
454 : MW912340.1
S = 0
455 : MW912400.1
S = 0.01
456 : MW912449.1
S = 0.01
457 : MW912471.1
S = 0
458 : MW912493.1
S = 0.01
459 : MW913826.1
S = 0.02
460 : MW914410.1
S = 0.02
461 : MW914411.1
S = 0.02
462 : MW914415.1
S = 0.02
463 : MW915187.1
S = 0.01
464 : MW915209.1
S = 0
465 : MW915283.1
S = 0.01
466 : MW908127.1
S = 0.01
467 : MW908289.1
S = 0
468 : MW908507.1
S = 0
469 : MW908736.1
S = 0.02
470 : MW909170.1
S = 0
471 : MW892330.1
S = 0
472 : MW892702.1
S = 0
473 : MW894068.1
S = 0
474 : MW872690.1
S = 0.01
475 : MW850614.1
S = 0.01
476 : MW850627.1
S = 0.01
477 : MW834807.1
S = 0.01
478 : MW834974.1
S = 0.02
479 : MW834975.1
S = 0.02
480 : MW807979.1
S = 0
481 : MW520923.1
S = 0
W.1
482 : MW851843.1
S = 0

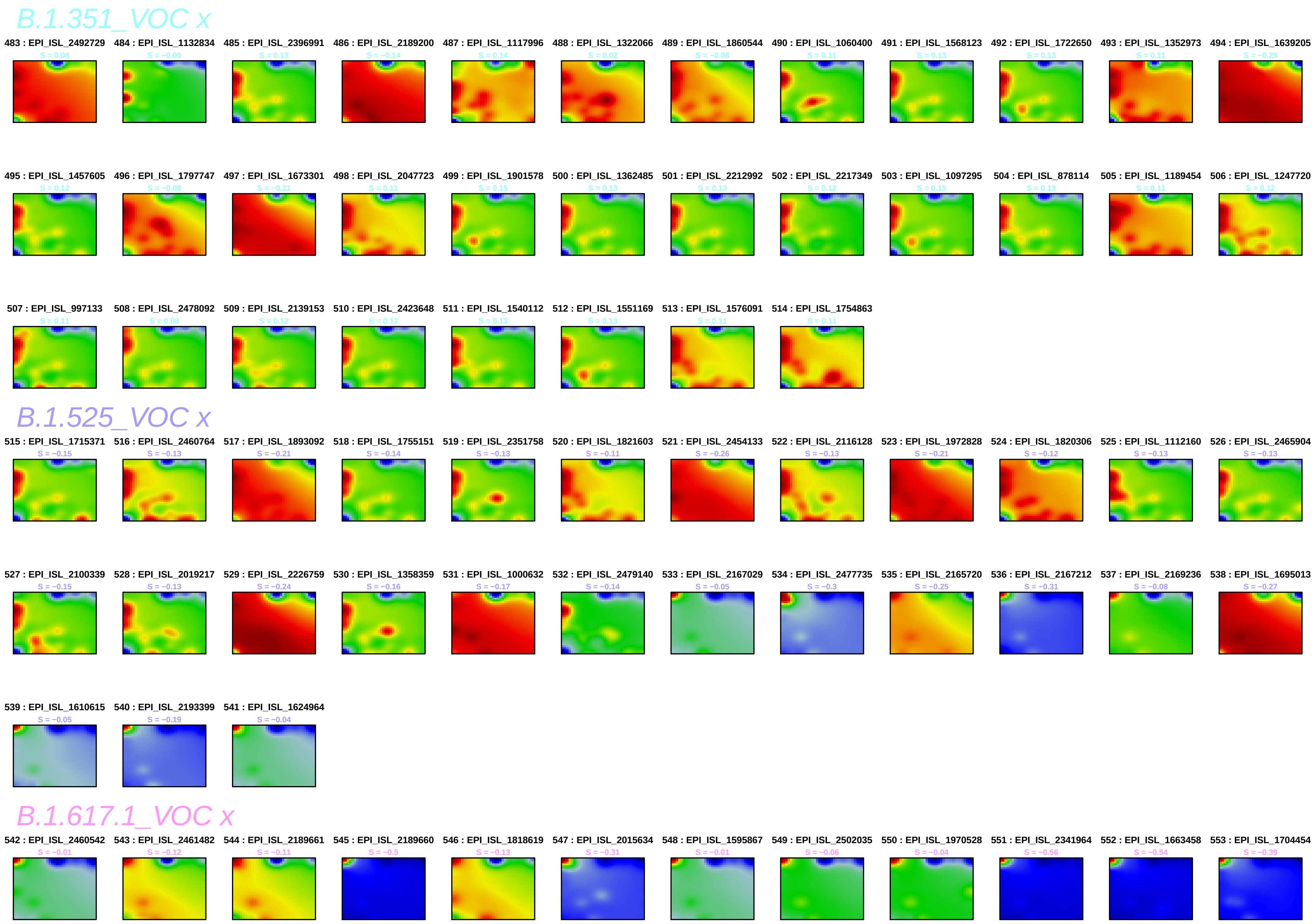

B.1.351_VOC x
483 : EPI_ISL_2492729
S = 0.06
484 : EPI_ISL_1132834
S = −0.09
485 : EPI_ISL_2396991
S = 0.13
486 : EPI_ISL_2189200
S = −0.14
487 : EPI_ISL_1117996
S = 0.14
488 : EPI_ISL_1322066
S = 0.02
489 : EPI_ISL_1860544
S = −0.08
490 : EPI_ISL_1060400
S = 0.11
491 : EPI_ISL_1568123
S = 0.13
492 : EPI_ISL_1722650
S = 0.13
493 : EPI_ISL_1352973
S = 0.11
494 : EPI_ISL_1639205
S = −0.29
495 : EPI_ISL_1457605
S = 0.12
496 : EPI_ISL_1797747
S = −0.08
497 : EPI_ISL_1673301
S = −0.21
498 : EPI_ISL_2047723
S = 0.11
499 : EPI_ISL_1901578
S = 0.15
500 : EPI_ISL_1362485
S = 0.13
501 : EPI_ISL_2212992
S = 0.13
502 : EPI_ISL_2217349
S = 0.12
503 : EPI_ISL_1097295
S = 0.15
504 : EPI_ISL_878114
S = 0.13
505 : EPI_ISL_1189454
S = 0.11
506 : EPI_ISL_1247720
S = 0.12
507 : EPI_ISL_997133
S = 0.11
508 : EPI_ISL_2478092
S = 0.08
509 : EPI_ISL_2139153
S = 0.12
510 : EPI_ISL_2423648
S = 0.12
511 : EPI_ISL_1540112
S = 0.13
512 : EPI_ISL_1551169
S = 0.13
513 : EPI_ISL_1576091
S = 0.11
514 : EPI_ISL_1754863
S = 0.11
B.1.525_VOC x
515 : EPI_ISL_1715371
S = −0.15
516 : EPI_ISL_2460764
S = −0.13
517 : EPI_ISL_1893092
S = −0.21
518 : EPI_ISL_1755151
S = −0.14
519 : EPI_ISL_2351758
S = −0.13
520 : EPI_ISL_1821603
S = −0.11
521 : EPI_ISL_2454133
S = −0.26
522 : EPI_ISL_2116128
S = −0.13
523 : EPI_ISL_1972828
S = −0.21
524 : EPI_ISL_1820306
S = −0.12
525 : EPI_ISL_1112160
S = −0.13
526 : EPI_ISL_2465904
S = −0.13
527 : EPI_ISL_2100339
S = −0.15
528 : EPI_ISL_2019217
S = −0.13
529 : EPI_ISL_2226759
S = −0.24
530 : EPI_ISL_1358359
S = −0.16
531 : EPI_ISL_1000632
S = −0.17
532 : EPI_ISL_2479140
S = −0.14
533 : EPI_ISL_2167029
S = −0.05
534 : EPI_ISL_2477735
S = −0.3
535 : EPI_ISL_2165720
S = −0.25
536 : EPI_ISL_2167212
S = −0.31
537 : EPI_ISL_2169236
S = −0.08
538 : EPI_ISL_1695013
S = −0.27
539 : EPI_ISL_1610615
S = −0.05
540 : EPI_ISL_2193399
S = −0.19
541 : EPI_ISL_1624964
S = −0.04
B.1.617.1_VOC x
542 : EPI_ISL_2460542
S = −0.01
543 : EPI_ISL_2461482
S = −0.12
544 : EPI_ISL_2189661
S = −0.11
545 : EPI_ISL_2189660
S = −0.5
546 : EPI_ISL_1818619
S = −0.13
547 : EPI_ISL_2015634
S = −0.31
548 : EPI_ISL_1595867
S = −0.01
549 : EPI_ISL_2502035
S = −0.06
550 : EPI_ISL_1970528
S = −0.04
551 : EPI_ISL_2341964
S = −0.56
552 : EPI_ISL_1663458
S = −0.54
553 : EPI_ISL_1704454
S = −0.39

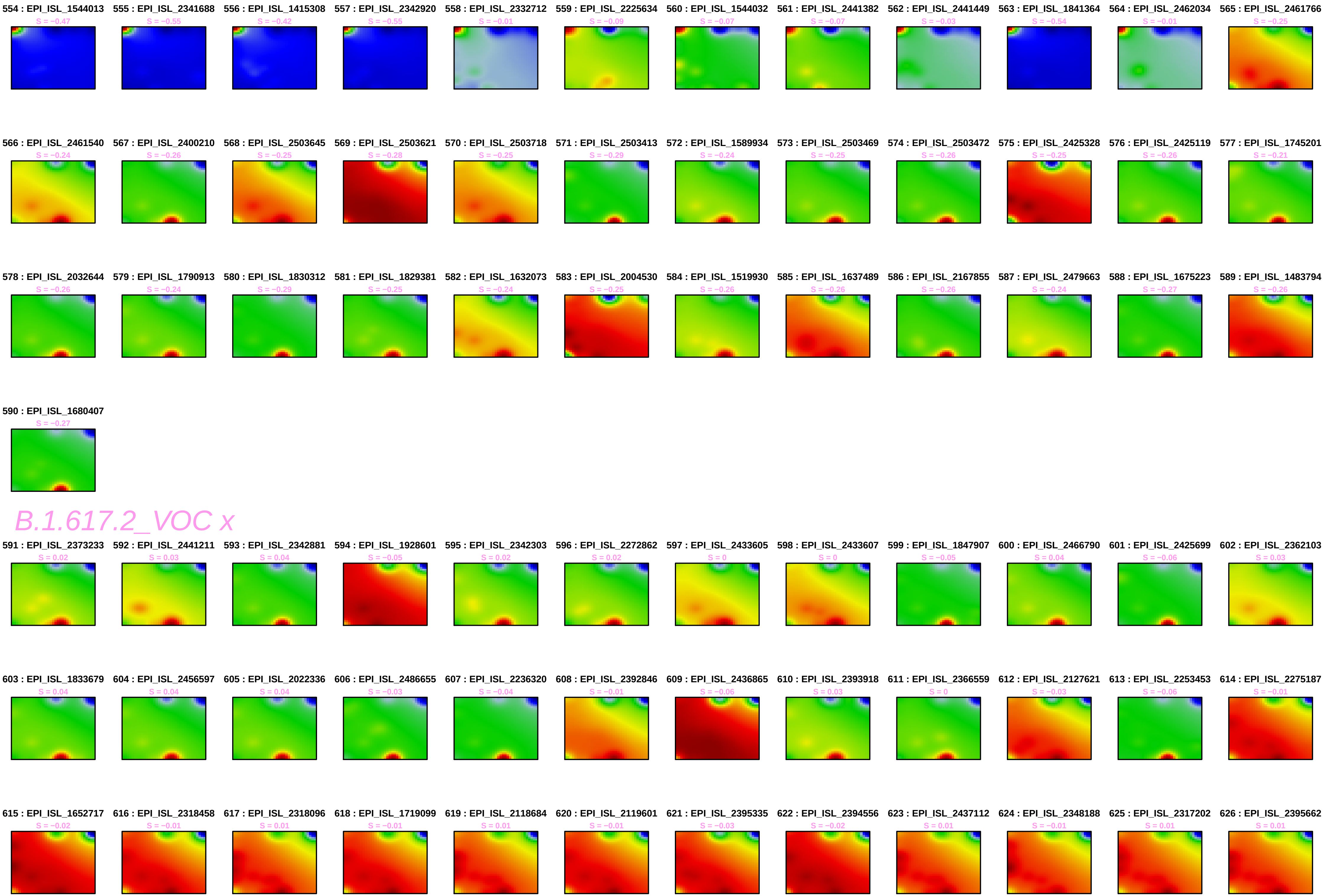
554 : EPI_ISL_1544013
S = −0.47
555 : EPI_ISL_2341688
S = −0.55
556 : EPI_ISL_1415308
S = −0.42
557 : EPI_ISL_2342920
S = −0.55
558 : EPI_ISL_2332712
S = −0.01
559 : EPI_ISL_2225634
S = −0.09
560 : EPI_ISL_1544032
S = −0.07
561 : EPI_ISL_2441382
S = −0.07
562 : EPI_ISL_2441449
S = −0.03
563 : EPI_ISL_1841364
S = −0.54
564 : EPI_ISL_2462034
S = −0.01
565 : EPI_ISL_2461766
S = −0.25
566 : EPI_ISL_2461540
S = −0.24
567 : EPI_ISL_2400210
S = −0.26
568 : EPI_ISL_2503645
S = −0.25
569 : EPI_ISL_2503621
S = −0.28
570 : EPI_ISL_2503718
S = −0.25
571 : EPI_ISL_2503413
S = −0.29
572 : EPI_ISL_1589934
S = −0.24
573 : EPI_ISL_2503469
S = −0.25
574 : EPI_ISL_2503472
S = −0.26
575 : EPI_ISL_2425328
S = −0.25
576 : EPI_ISL_2425119
S = −0.26
577 : EPI_ISL_1745201
S = −0.21
578 : EPI_ISL_2032644
S = −0.26
579 : EPI_ISL_1790913
S = −0.24
580 : EPI_ISL_1830312
S = −0.29
581 : EPI_ISL_1829381
S = −0.25
582 : EPI_ISL_1632073
S = −0.24
583 : EPI_ISL_2004530
S = −0.25
584 : EPI_ISL_1519930
S = −0.26
585 : EPI_ISL_1637489
S = −0.26
586 : EPI_ISL_2167855
S = −0.26
587 : EPI_ISL_2479663
S = −0.24
588 : EPI_ISL_1675223
S = −0.27
589 : EPI_ISL_1483794
S = −0.26
590 : EPI_ISL_1680407
S = −0.27
B.1.617.2_VOC x
591 : EPI_ISL_2373233
S = 0.02
592 : EPI_ISL_2441211
S = 0.03
593 : EPI_ISL_2342881
S = 0.04
594 : EPI_ISL_1928601
S = −0.05
595 : EPI_ISL_2342303
S = 0.02
596 : EPI_ISL_2272862
S = 0.02
597 : EPI_ISL_2433605
S = 0
598 : EPI_ISL_2433607
S = 0
599 : EPI_ISL_1847907
S = −0.05
600 : EPI_ISL_2466790
S = 0.04
601 : EPI_ISL_2425699
S = −0.06
602 : EPI_ISL_2362103
S = 0.03
603 : EPI_ISL_1833679
S = 0.04
604 : EPI_ISL_2456597
S = 0.04
605 : EPI_ISL_2022336
S = 0.04
606 : EPI_ISL_2486655
S = −0.03
607 : EPI_ISL_2236320
S = −0.04
608 : EPI_ISL_2392846
S = −0.01
609 : EPI_ISL_2436865
S = −0.06
610 : EPI_ISL_2393918
S = 0.03
611 : EPI_ISL_2366559
S = 0
612 : EPI_ISL_2127621
S = −0.03
613 : EPI_ISL_2253453
S = −0.06
614 : EPI_ISL_2275187
S = −0.01
615 : EPI_ISL_1652717
S = −0.02
616 : EPI_ISL_2318458
S = −0.01
617 : EPI_ISL_2318096
S = 0.01
618 : EPI_ISL_1719099
S = −0.01
619 : EPI_ISL_2118684
S = 0.01
620 : EPI_ISL_2119601
S = −0.01
621 : EPI_ISL_2395335
S = −0.03
622 : EPI_ISL_2394556
S = −0.02
623 : EPI_ISL_2437112
S = 0.01
624 : EPI_ISL_2348188
S = −0.01
625 : EPI_ISL_2317202
S = 0.01
626 : EPI_ISL_2395662
S = 0.01

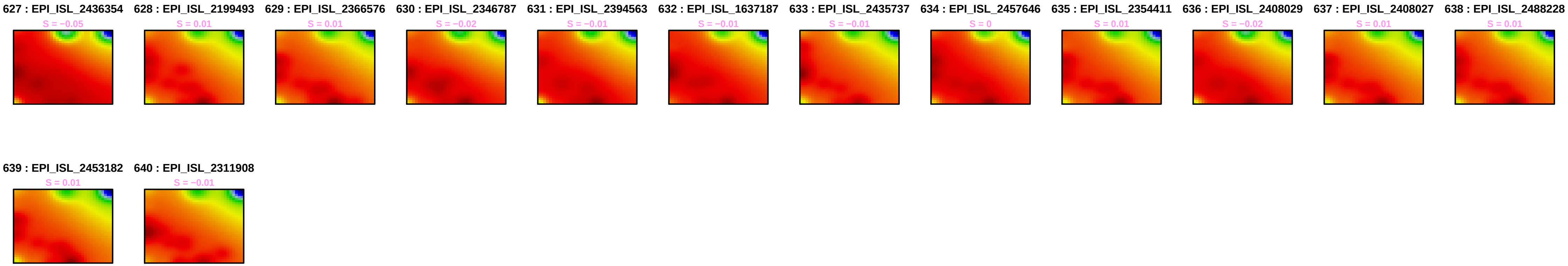
627 : EPI_ISL_2436354
S = −0.05
628 : EPI_ISL_2199493
S = 0.01
629 : EPI_ISL_2366576
S = 0.01
630 : EPI_ISL_2346787
S = −0.02
631 : EPI_ISL_2394563
S = −0.01
632 : EPI_ISL_1637187
S = −0.01
633 : EPI_ISL_2435737
S = −0.01
634 : EPI_ISL_2457646
S = 0
635 : EPI_ISL_2354411
S = 0.01
636 : EPI_ISL_2408029
S = −0.02
637 : EPI_ISL_2408027
S = 0.01
638 : EPI_ISL_2488228
S = 0.01
639 : EPI_ISL_2453182
S = 0.01
640 : EPI_ISL_2311908
S = −0.01

C.37_VOC x

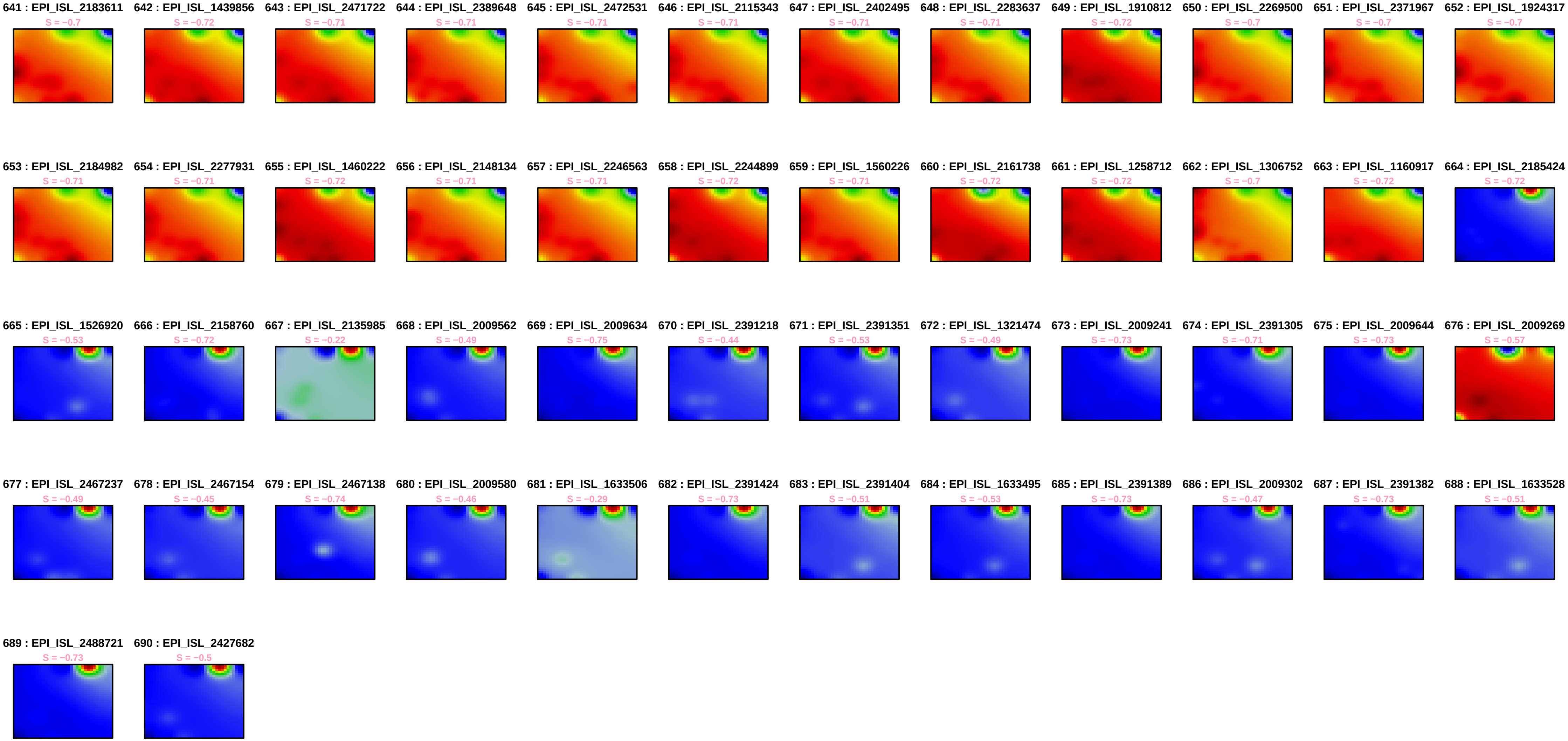
641 : EPI_ISL_2183611
S = −0.7
642 : EPI_ISL_1439856
S = −0.72
643 : EPI_ISL_2471722
S = −0.71
644 : EPI_ISL_2389648
S = −0.71
645 : EPI_ISL_2472531
S = −0.71
646 : EPI_ISL_2115343
S = −0.71
647 : EPI_ISL_2402495
S = −0.71
648 : EPI_ISL_2283637
S = −0.71
649 : EPI_ISL_1910812
S = −0.72
650 : EPI_ISL_2269500
S = −0.7
651 : EPI_ISL_2371967
S = −0.7
652 : EPI_ISL_1924317
S = −0.7
653 : EPI_ISL_2184982
S = −0.71
654 : EPI_ISL_2277931
S = −0.71
655 : EPI_ISL_1460222
S = −0.72
656 : EPI_ISL_2148134
S = −0.71
657 : EPI_ISL_2246563
S = −0.71
658 : EPI_ISL_2244899
S = −0.72
659 : EPI_ISL_1560226
S = −0.71
660 : EPI_ISL_2161738
S = −0.72
661 : EPI_ISL_1258712
S = −0.72
662 : EPI_ISL_1306752
S = −0.7
663 : EPI_ISL_1160917
S = −0.72
664 : EPI_ISL_2185424
S = −0.72
665 : EPI_ISL_1526920
S = −0.53
666 : EPI_ISL_2158760
S = −0.72
667 : EPI_ISL_2135985
S = −0.22
668 : EPI_ISL_2009562
S = −0.49
669 : EPI_ISL_2009634
S = −0.75
670 : EPI_ISL_2391218
S = −0.44
671 : EPI_ISL_2391351
S = −0.53
672 : EPI_ISL_1321474
S = −0.49
673 : EPI_ISL_2009241
S = −0.73
674 : EPI_ISL_2391305
S = −0.71
675 : EPI_ISL_2009644
S = −0.73
676 : EPI_ISL_2009269
S = −0.57
677 : EPI_ISL_2467237
S = −0.49
678 : EPI_ISL_2467154
S = −0.45
679 : EPI_ISL_2467138
S = −0.74
680 : EPI_ISL_2009580
S = −0.46
681 : EPI_ISL_1633506
S = −0.29
682 : EPI_ISL_2391424
S = −0.73
683 : EPI_ISL_2391404
S = −0.51
684 : EPI_ISL_1633495
S = −0.53
685 : EPI_ISL_2391389
S = −0.73
686 : EPI_ISL_2009302
S = −0.47
687 : EPI_ISL_2391382
S = −0.73
688 : EPI_ISL_1633528
S = −0.51
689 : EPI_ISL_2488721
S = −0.73
690 : EPI_ISL_2427682
S = −0.5

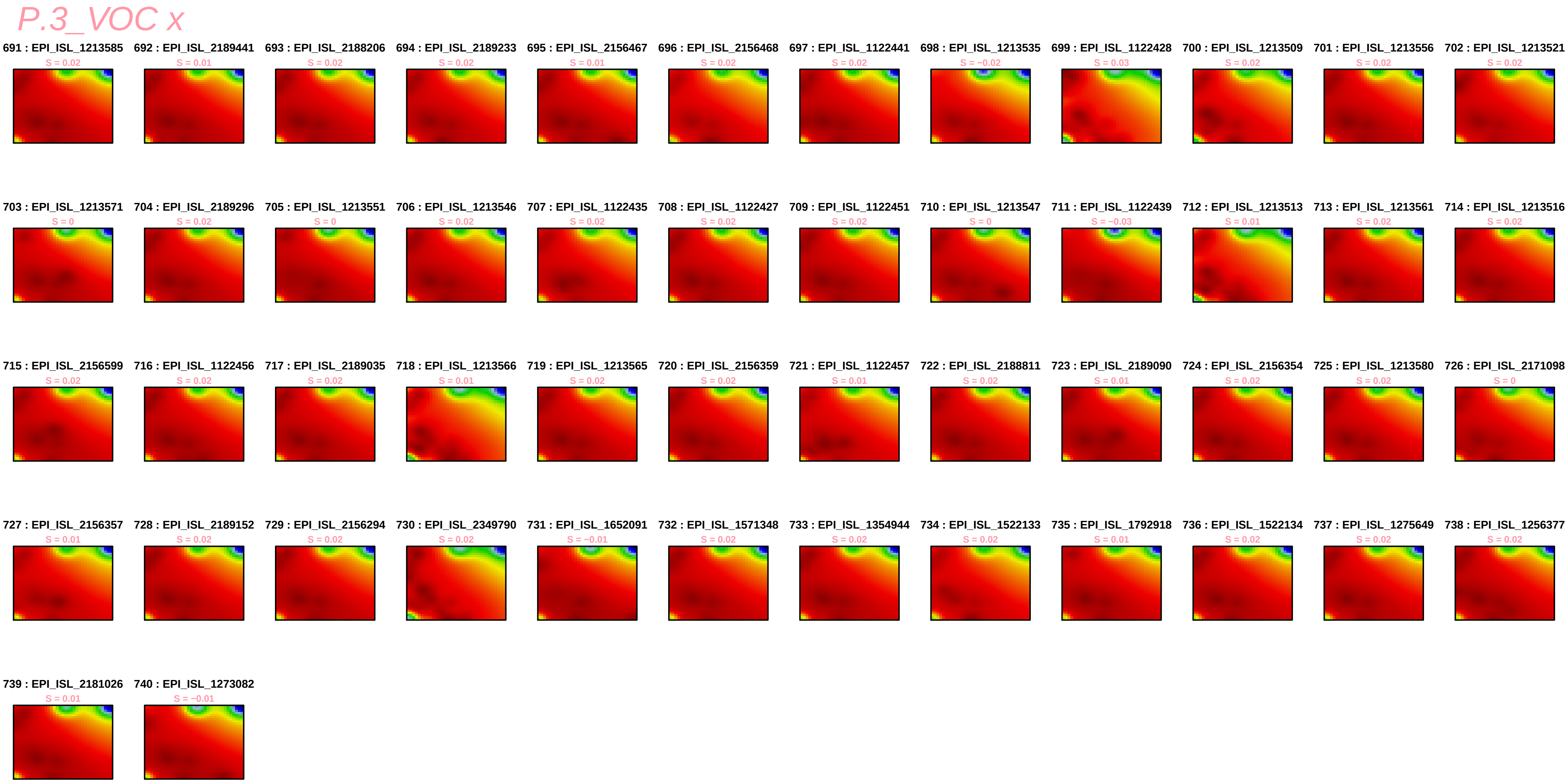
P.3_VOC x
691 : EPI_ISL_1213585
S = 0.02
692 : EPI_ISL_2189441
S = 0.01
693 : EPI_ISL_2188206
S = 0.02
694 : EPI_ISL_2189233
S = 0.02
695 : EPI_ISL_2156467
S = 0.01
696 : EPI_ISL_2156468
S = 0.02
697 : EPI_ISL_1122441
S = 0.02
698 : EPI_ISL_1213535
S = −0.02
699 : EPI_ISL_1122428
S = 0.03
700 : EPI_ISL_1213509
S = 0.02
701 : EPI_ISL_1213556
S = 0.02
702 : EPI_ISL_1213521
S = 0.02
703 : EPI_ISL_1213571
S = 0
704 : EPI_ISL_2189296
S = 0.02
705 : EPI_ISL_1213551
S = 0
706 : EPI_ISL_1213546
S = 0.02
707 : EPI_ISL_1122435
S = 0.02
708 : EPI_ISL_1122427
S = 0.02
709 : EPI_ISL_1122451
S = 0.02
710 : EPI_ISL_1213547
S = 0
711 : EPI_ISL_1122439
S = −0.03
712 : EPI_ISL_1213513
S = 0.01
713 : EPI_ISL_1213561
S = 0.02
714 : EPI_ISL_1213516
S = 0.02
715 : EPI_ISL_2156599
S = 0.02
716 : EPI_ISL_1122456
S = 0.02
717 : EPI_ISL_2189035
S = 0.02
718 : EPI_ISL_1213566
S = 0.01
719 : EPI_ISL_1213565
S = 0.02
720 : EPI_ISL_2156359
S = 0.02
721 : EPI_ISL_1122457
S = 0.01
722 : EPI_ISL_2188811
S = 0.02
723 : EPI_ISL_2189090
S = 0.01
724 : EPI_ISL_2156354
S = 0.02
725 : EPI_ISL_1213580
S = 0.02
726 : EPI_ISL_2171098
S = 0
727 : EPI_ISL_2156357
S = 0.01
728 : EPI_ISL_2189152
S = 0.02
729 : EPI_ISL_2156294
S = 0.02
730 : EPI_ISL_2349790
S = 0.02
731 : EPI_ISL_1652091
S = −0.01
732 : EPI_ISL_1571348
S = 0.02
733 : EPI_ISL_1354944
S = 0.02
734 : EPI_ISL_1522133
S = 0.02
735 : EPI_ISL_1792918
S = 0.01
736 : EPI_ISL_1522134
S = 0.02
737 : EPI_ISL_1275649
S = 0.02
738 : EPI_ISL_1256377
S = 0.02
739 : EPI_ISL_2181026
S = 0.01
740 : EPI_ISL_1273082
S = −0.01

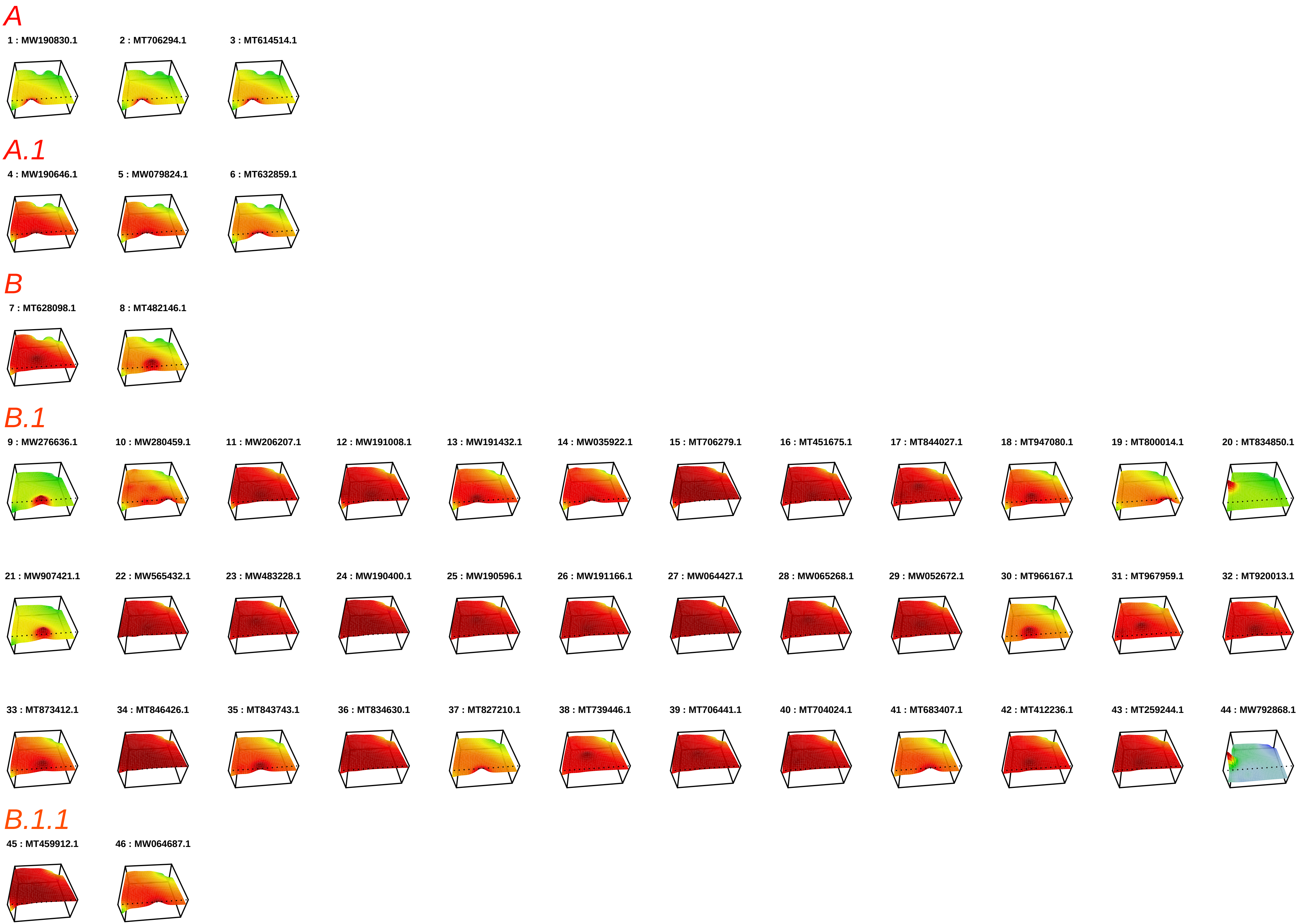
A
1 : MW190830.1
2 : MT706294.1
3 : MT614514.1
A.1
4 : MW190646.1
5 : MW079824.1
6 : MT632859.1
B
7 : MT628098.1
8 : MT482146.1
B.1
9 : MW276636.1
10 : MW280459.1
11 : MW206207.1
12 : MW191008.1
13 : MW191432.1
14 : MW035922.1
15 : MT706279.1
16 : MT451675.1
17 : MT844027.1
18 : MT947080.1
19 : MT800014.1
20 : MT834850.1
21 : MW907421.1
22 : MW565432.1
23 : MW483228.1
24 : MW190400.1
25 : MW190596.1
26 : MW191166.1
27 : MW064427.1
28 : MW065268.1
29 : MW052672.1
30 : MT966167.1
31 : MT967959.1
32 : MT920013.1
33 : MT873412.1
34 : MT846426.1
35 : MT843743.1
36 : MT834630.1
37 : MT827210.1
38 : MT739446.1
39 : MT706441.1
40 : MT704024.1
41 : MT683407.1
42 : MT412236.1
43 : MT259244.1
44 : MW792868.1
B.1.1
45 : MT459912.1
46 : MW064687.1

B.1.1.136

47 : MT972137.1

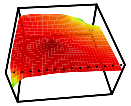

B.1.1.169

48 : MW279423.1

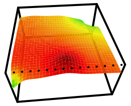

B.1.1.186

49 : MW719920.1

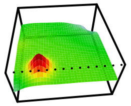

B.1.1.205

50 : MW306370.1

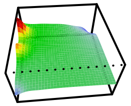

B.1.1.222

51 : MW681161.1

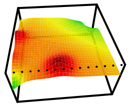

52 : MW653612.1

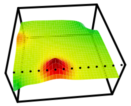

B.1.1.228

53 : MW645720.1

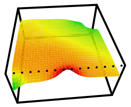

54 : MW206329.1

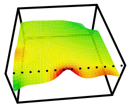

B.1.1.231

55 : MW521681.1

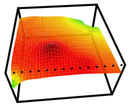

B.1.1.244

56 : MW206601.1

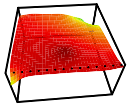

B.1.1.291

57 : MT831212.1

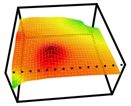

58 : MT831708.1

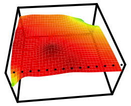

B.1.1.316

59 : MW864396.1

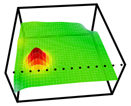

60 : MW666340.1

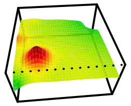

B.1.1.416

61 : MW738279.1

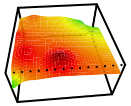

B.1.1.432

62 : MW763480.1

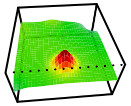

63 : MW521794.1

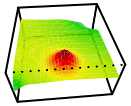

B.1.1.434

64 : MW668844.1

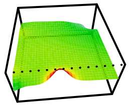

B.1.1.519

65 : MW903995.1

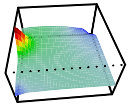

66 : MW849227.1

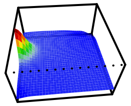

67 : MW849880.1

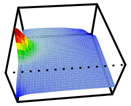

68 : MW681413.1

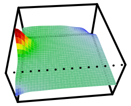

69 : MW667310.1

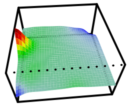

B.1.1.7 _VOC

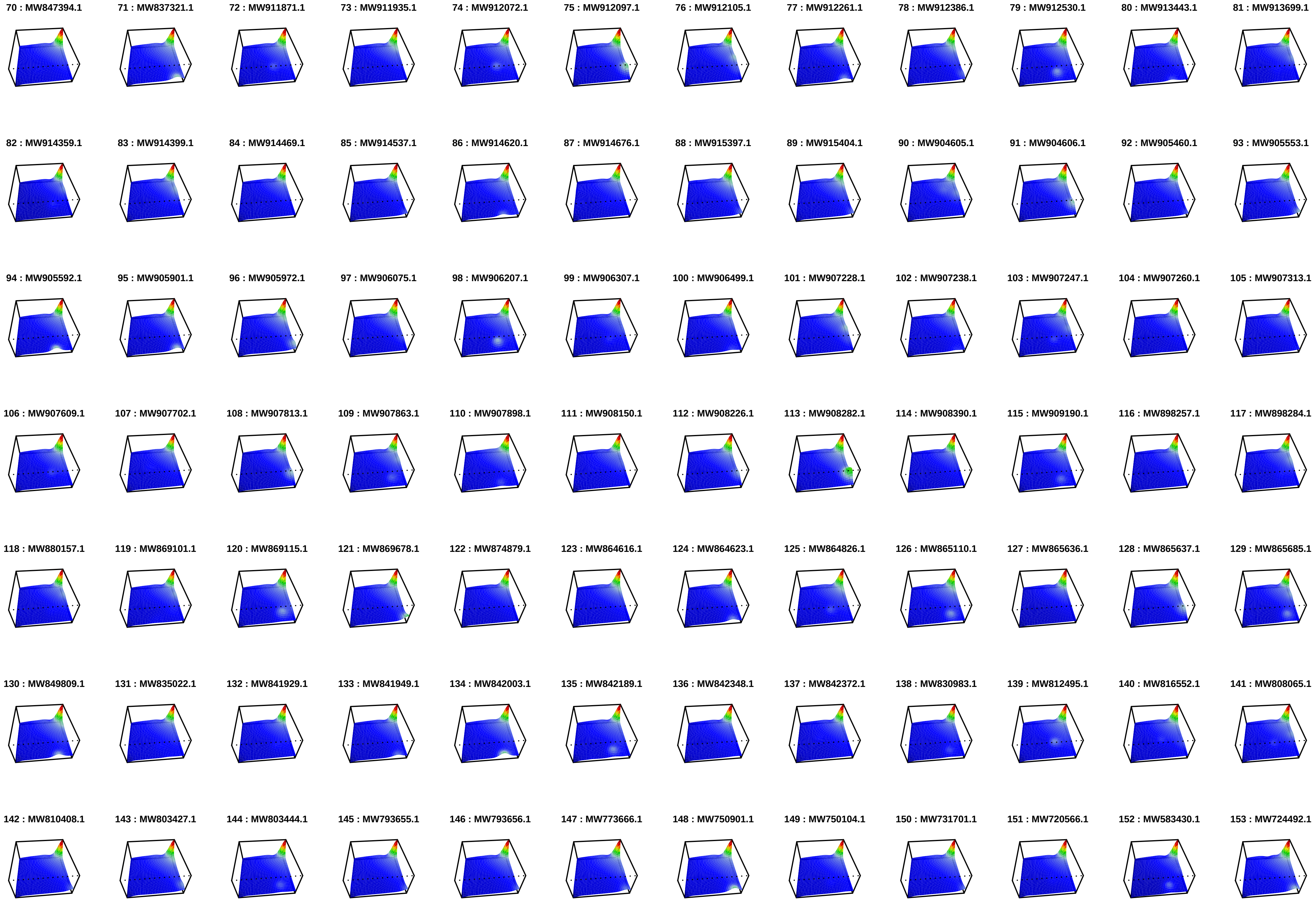

70 : MW847394.1
71 : MW837321.1
72 : MW911871.1
73 : MW911935.1
74 : MW912072.1
75 : MW912097.1
76 : MW912105.1
77 : MW912261.1
78 : MW912386.1
79 : MW912530.1
80 : MW913443.1
81 : MW913699.1
82 : MW914359.1
83 : MW914399.1
84 : MW914469.1
85 : MW914537.1
86 : MW914620.1
87 : MW914676.1
88 : MW915397.1
89 : MW915404.1
90 : MW904605.1
91 : MW904606.1
92 : MW905460.1
93 : MW905553.1
94 : MW905592.1
95 : MW905901.1
96 : MW905972.1
97 : MW906075.1
98 : MW906207.1
99 : MW906307.1
100 : MW906499.1
101 : MW907228.1
102 : MW907238.1
103 : MW907247.1
104 : MW907260.1
105 : MW907313.1
106 : MW907609.1
107 : MW907702.1
108 : MW907813.1
109 : MW907863.1
110 : MW907898.1
111 : MW908150.1
112 : MW908226.1
113 : MW908282.1
114 : MW908390.1
115 : MW909190.1
116 : MW898257.1
117 : MW898284.1
118 : MW880157.1
119 : MW869101.1
120 : MW869115.1
121 : MW869678.1
122 : MW874879.1
123 : MW864616.1
124 : MW864623.1
125 : MW864826.1
126 : MW865110.1
127 : MW865636.1
128 : MW865637.1
129 : MW865685.1
130 : MW849809.1
131 : MW835022.1
132 : MW841929.1
133 : MW841949.1
134 : MW842003.1
135 : MW842189.1
136 : MW842348.1
137 : MW842372.1
138 : MW830983.1
139 : MW812495.1
140 : MW816552.1
141 : MW808065.1
142 : MW810408.1
143 : MW803427.1
144 : MW803444.1
145 : MW793655.1
146 : MW793656.1
147 : MW773666.1
148 : MW750901.1
149 : MW750104.1
150 : MW731701.1
151 : MW720566.1
152 : MW583430.1
153 : MW724492.1

B.1.110

154 : MW364999.1

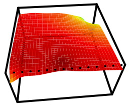

B.1.110.3

155 : MW825510.1

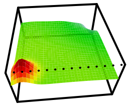

156 : MW813533.1

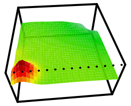

157 : MW772211.1

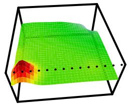

B.1.112

158 : MW206178.1

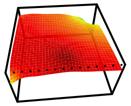

B.1.139

159 : MT750131.1

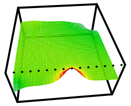

B.1.181

160 : MW079838.1

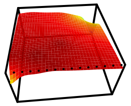

B.1.2

161 : MW903075.1

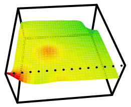

162 : MW898282.1

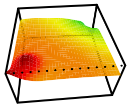

163 : MW891025.1

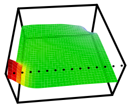

164 : MW892093.1

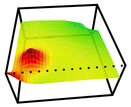

165 : MW897491.1

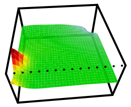

166 : MW888010.1

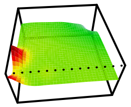

167 : MW871122.1

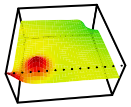

168 : MW865415.1

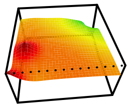

169 : MW865437.1

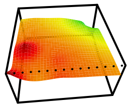

170 : MW849046.1

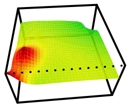

171 : MW849140.1

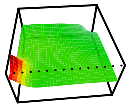

172 : MW850290.1

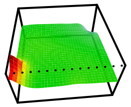

173 : MW837489.1

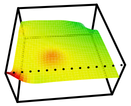

174 : MW813248.1

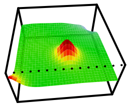

175 : MW816195.1

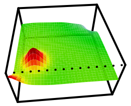

176 : MW804890.1

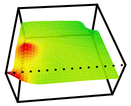

177 : MW782913.1

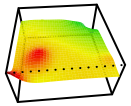

178 : MW782957.1

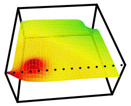

179 : MW773379.1

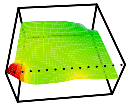

180 : MW766758.1

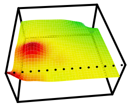

181 : MW739293.1

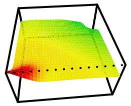

182 : MW739740.1

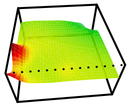

183 : MW731110.1

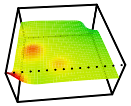

184 : MW731198.1

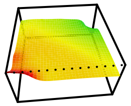

185 : MW728398.1 186 : MW728607.1 187 : MW728726.1 188 : MW722108.1 189 : MW720342.1 190 : MW707271.1 191 : MW708115.1 192 : MW700514.1 193 : MW697032.1 194 : MW697898.1 195 : MW698106.1 196 : MW681313.1

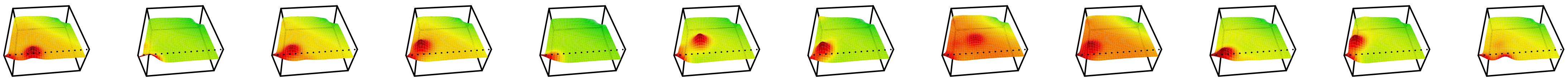

197 : MW665629.1 198 : MW665748.1 199 : MW669397.1 200 : MW645572.1 201 : MW643455.1 202 : MW634509.1 203 : MW635041.1 204 : MW640098.1 205 : MW640521.1 206 : MW640762.1 207 : MW640769.1 208 : MW616854.1

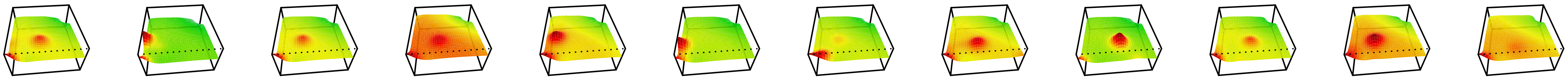

209 : MW565317.1 210 : MW562037.1 211 : MW559203.1 212 : MW550176.1 213 : MW550243.1 214 : MW542994.1 215 : MW524026.1 216 : MW495215.1 217 : MW474332.1 218 : MW420051.1 219 : MW173440.1 220 : MW035554.1

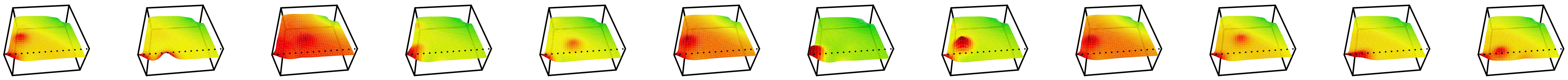

B.1.232

221 : MW700796.1 222 : MW638159.1

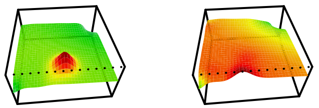

B.1.234

223 : MW792553.1 224 : MW728504.1 225 : MW666640.1 226 : MW077468.1

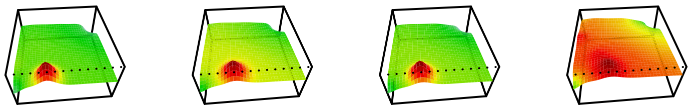

B.1.240

227 : MW566812.1 228 : MW420061.1 229 : MW420568.1 230 : MW206309.1

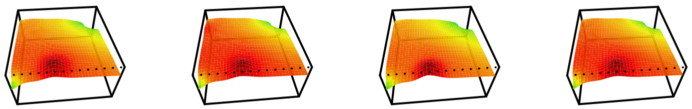

B.1.243

231 : MW782837.1 232 : MW653595.1 233 : MW635155.1 234 : MW565465.1 235 : MW420588.1 236 : MW276556.1 237 : MW036063.1

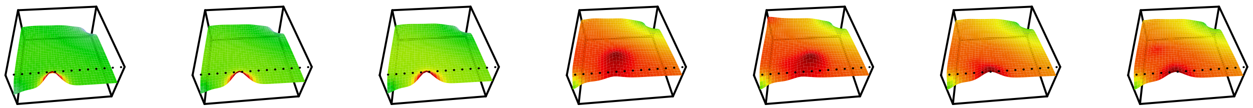

B.1.258.21

238 : MW453084.1

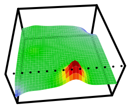

B.1.265

239 : MW708707.1

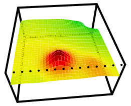

B.1.274

240 : MW206082.1

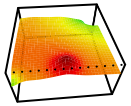

B.1.284

241 : MW064341.1

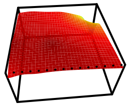

B.1.298

242 : MW709154.1

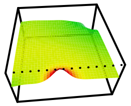

B.1.305

243 : MW815792.1

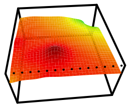

B.1.320

244 : MT641523.1

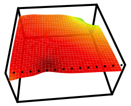

245 : MT628158.1

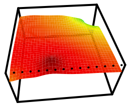

B.1.349

246 : MW668829.1

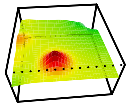

B.1.351 _VOC

247 : MW621453.1

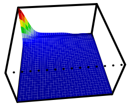

248 : MW913437.1

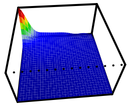

249 : MW914008.1

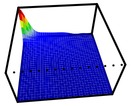

250 : MW914015.1

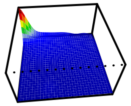

251 : MW914542.1

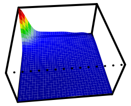

252 : MW905844.1

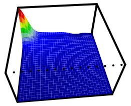

253 : MW907199.1

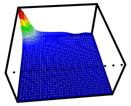

254 : MW907323.1

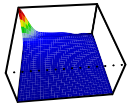

255 : MW908243.1

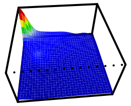

256 : MW908815.1

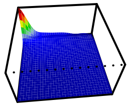

257 : MW909222.1

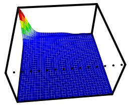

258 : MW849825.1

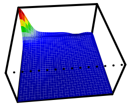

259 : MW795351.1

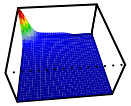

260 : MW796654.1

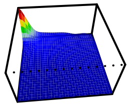

261 : MW792684.1

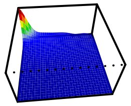

262 : MW792756.1

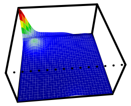

263 : MW793005.1

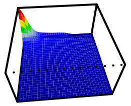

264 : MW773815.1

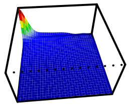

B.1.36.27

265 : MW691153.1

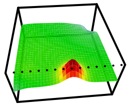

B.1.360

266 : MW064652.1

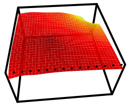

B.1.369

267 : MT843300.1

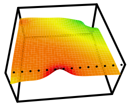

268 : MT827251.1

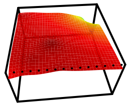

B.1.371

269 : MT994395.1

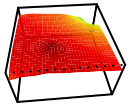

270 : MT642377.1

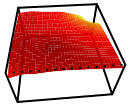

B.1.382

271 : MW471399.1

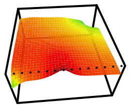

B.1.384

272 : MW206155.1

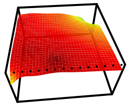

B.1.400

273 : MW634571.1

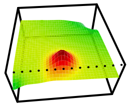

B.1.427 _VOC

274 : MW902062.1 | 275 : MW903304.1 | 276 : MW904442.1 | 277 : MW891068.1 | 278 : MW869965.1 | 279 : MW850286.1 | 280 : MW796674.1 | 281 : MW792927.1 | 282 : MW777189.1 | 283 : MW766808.1 | 284 : MW737960.1 | 285 : MW739415.1

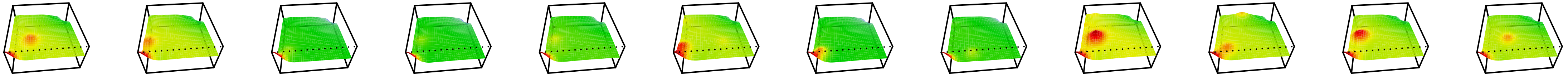

286 : MW730985.1 | 287 : MW731276.1 | 288 : MW731356.1 | 289 : MW731747.1 | 290 : MW638933.1 | 291 : MW564964.1

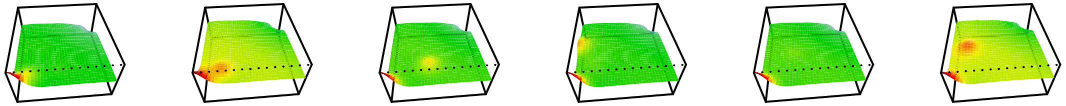

B.1.429 _VOC

292 : MW903146.1 | 293 : MW890985.1 | 294 : MW891299.1 | 295 : MW850582.1 | 296 : MW855628.1 | 297 : MW855781.1 | 298 : MW848299.1 | 299 : MW849087.1 | 300 : MW850114.1 | 301 : MW837289.1 | 302 : MW837390.1 | 303 : MW821827.1

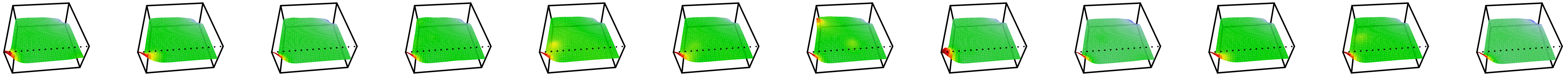

304 : MW796797.1 | 305 : MW796840.1 | 306 : MW788789.1 | 307 : MW792842.1 | 308 : MW792999.1 | 309 : MW793073.1 | 310 : MW780400.1 | 311 : MW781335.1 | 312 : MW767571.1 | 313 : MW763186.1 | 314 : MW766623.1 | 315 : MW766643.1

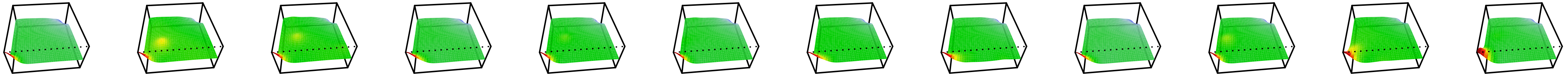

316 : MW766742.1

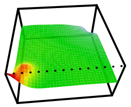

317 : MW739122.1

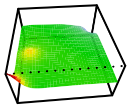

318 : MW731201.1

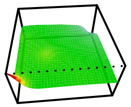

319 : MW731327.1

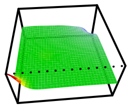

320 : MW731376.1

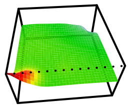

321 : MW731779.1

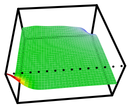

322 : MW734228.1

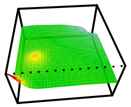

323 : MW734357.1

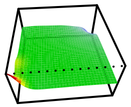

324 : MW708099.1

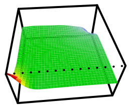

325 : MW708538.1

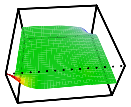

326 : MW701159.1

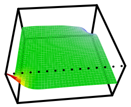

327 : MW701205.1

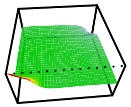

328 : MW702079.1

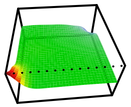

329 : MW702856.1

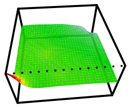

330 : MW703006.1

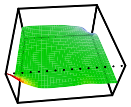

331 : MW692964.1

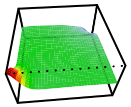

332 : MW545329.1

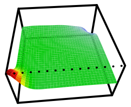

333 : MW460632.1

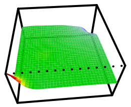

B.1.436

334 : MW279421.1

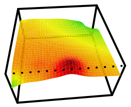

B.1.444

335 : MW420740.1

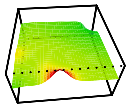

B.1.446

336 : MT358739.1

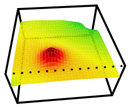

B.1.492

337 : MW645969.1

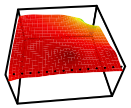

B.1.517

338 : MW782901.1

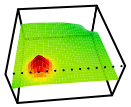

339 : MW783000.1

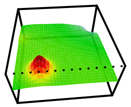

340 : MW715189.1

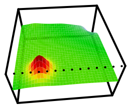

341 : MW550003.1

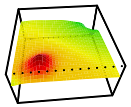

B.1.525 _VOC

342 : MW850613.1
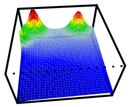
343 : MW851945.1
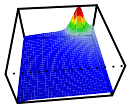
344 : MW851945.1
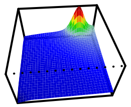
345 : MW852481.1
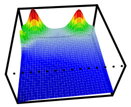
346 : MW809041.1
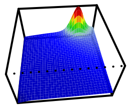
347 : MW809053.1
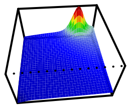
348 : MW809053.1
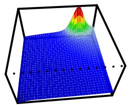
349 : MW809056.1
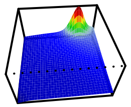
350 : MW809056.1
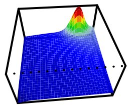
351 : MW809058.1
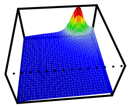
352 : MW809060.1
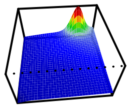
353 : MW809060.1
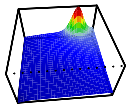
354 : MW809061.1
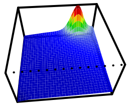
355 : MW809061.1
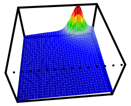
356 : MW751593.1
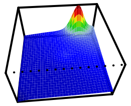
357 : MW751593.1
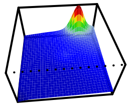
358 : MW751596.1
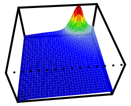
359 : MW751596.1
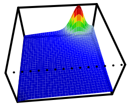
360 : MW713636.1
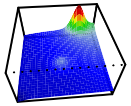
361 : MW709039.1
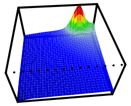
362 : MW578231.1
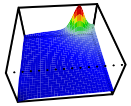
363 : MW560924.1
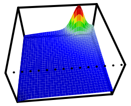
364 : MW560924.1
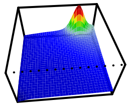

B.1.526

365 : MW890618.1
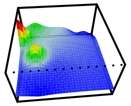
366 : MW891086.1
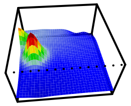
367 : MW897496.1
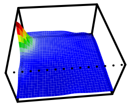
368 : MW880557.1
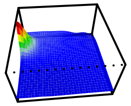
369 : MW779625.1
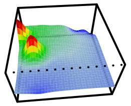

B.1.526.2

370 : MW890635.1
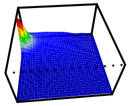
371 : MW892250.1
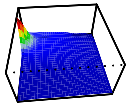
372 : MW892338.1
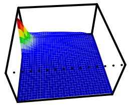

B.1.544

373 : MW703034.1
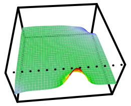

B.1.565

374 : MW850137.1
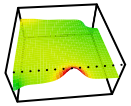

B.1.568

375 : MW708213.1
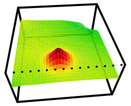

B.1.575

376 : MW902922.1

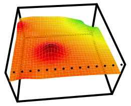

B.1.590

377 : MW191464.1

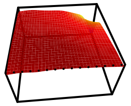

B.1.595

378 : MW735956.1

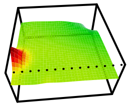

379 : MW728639.1

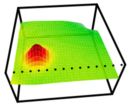

B.1.595.1

380 : MW286639.1

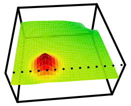

B.1.596

381 : MW904425.1

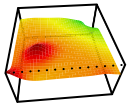

382 : MW891324.1

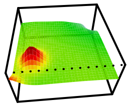

383 : MW820254.1

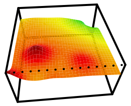

B.1.617.1 _VOC

384 : MW903294.1

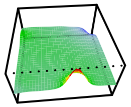

B.19

385 : MT438750.1

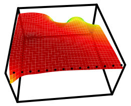

B.40

386 : MT507274.1

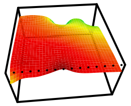

B.46

387 : MT534317.1

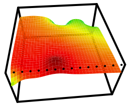

C.26

388 : MW365070.1

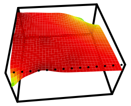

C.35

389 : MW454582.1

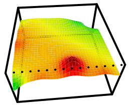

D.2

390 : MT970009.1

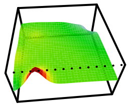

391 : MW321111.1

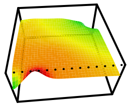

392 : MW321326.1

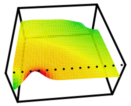

393 : MW277061.1

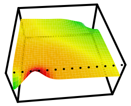

394 : MW277356.1

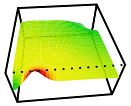

395 : MW184473.1

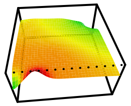

396 : MW184514.1

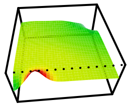

397 : MW184759.1

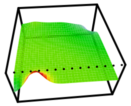

398 : MW185500.1

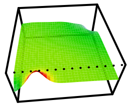

399 : MW153235.1

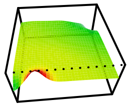

400 : MW153463.1

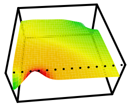

401 : MW153476.1

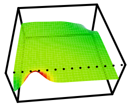

402 : MW154432.1

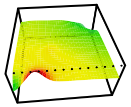

403 : MW155350.1

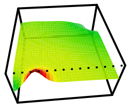

404 : MW156308.1

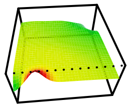

405 : MW156515.1

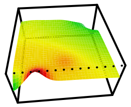

406 : MW156556.1

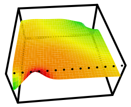

407 : MW157011.1

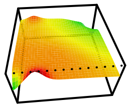

408 : MT969961.1

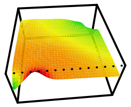

409 : MT970206.1

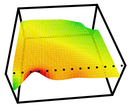

410 : MT970602.1

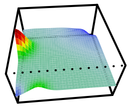

411 : MT970638.1

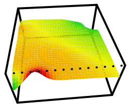

412 : MT970641.1

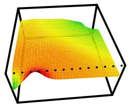

413 : MT970660.1

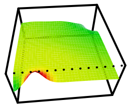

414 : MT970713.1

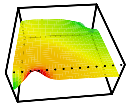

415 : MT970730.1

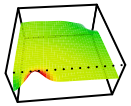

416 : MT970759.1

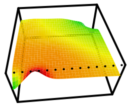

417 : MT970883.1

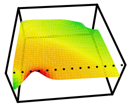

418 : MT970912.1

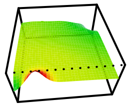

419 : MT970995.1

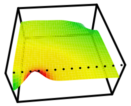

420 : MT970996.1

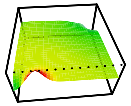

421 : MT971103.1

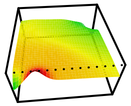

422 : MT971665.1

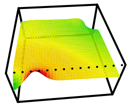

423 : MT971767.1

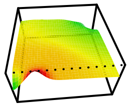

424 : MT971902.1

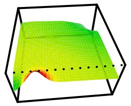

425 : MT971989.1

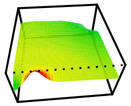

426 : MT972311.1 427 : MT972565.1 428 : MT972587.1 429 : MT973080.1 430 : MT973202.1 431 : MT973284.1

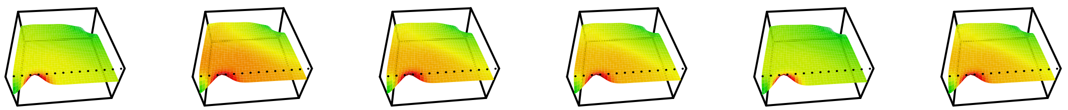

P.1 _VOC

432 : MW911880.1 433 : MW912245.1 434 : MW912407.1 435 : MW912428.1 436 : MW907170.1 437 : MW907250.1 438 : MW907917.1 439 : MW907928.1 440 : MW892126.1 441 : MW876960.1 442 : MW877029.1 443 : MW834962.1

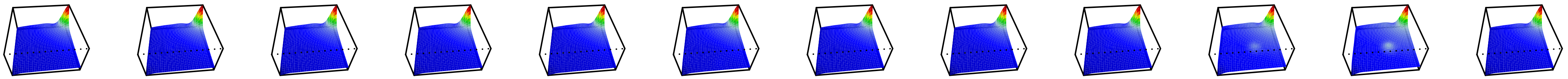

444 : MW834965.1 445 : MW834977.1 446 : MW834982.1 447 : MW837448.1 448 : MW808135.1 449 : MW912118.1 450 : MW912124.1 451 : MW912172.1 452 : MW912219.1 453 : MW912312.1 454 : MW912340.1 455 : MW912400.1

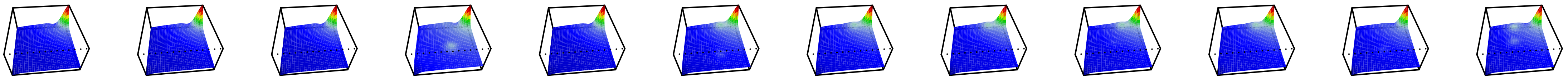

456 : MW912449.1 457 : MW912471.1 458 : MW912493.1 459 : MW913826.1 460 : MW914410.1 461 : MW914411.1 462 : MW914415.1 463 : MW915187.1 464 : MW915209.1 465 : MW915283.1 466 : MW908127.1 467 : MW908289.1

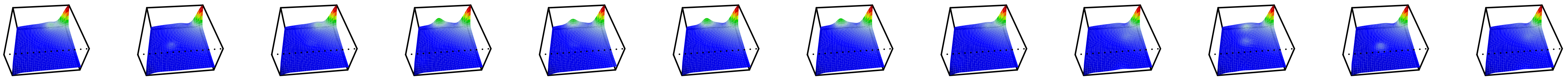

468 : MW908507.1 469 : MW908736.1 470 : MW909170.1 471 : MW892330.1 472 : MW892702.1 473 : MW894068.1 474 : MW872690.1 475 : MW850614.1 476 : MW850627.1 477 : MW834807.1 478 : MW834974.1 479 : MW834975.1

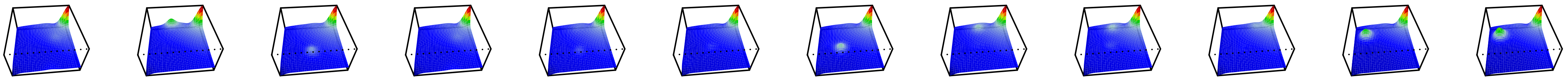

480 : MW807979.1 481 : MW520923.1

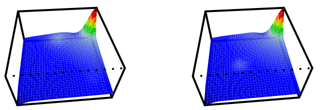

W.1

482 : MW851843.1

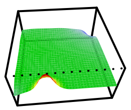

B.1.351_VOC x

483 : EPI_ISL_2492729 484 : EPI_ISL_1132834 485 : EPI_ISL_2396991 486 : EPI_ISL_2189200 487 : EPI_ISL_1117996 488 : EPI_ISL_1322066 489 : EPI_ISL_1860544 490 : EPI_ISL_1060400 491 : EPI_ISL_1568123 492 : EPI_ISL_1722650 493 : EPI_ISL_1352973 494 : EPI_ISL_1639205

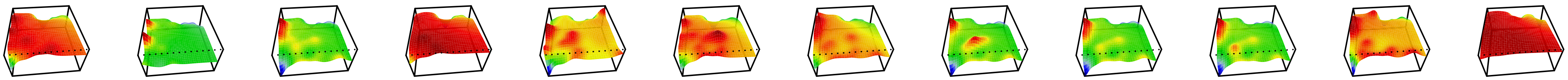

495 : EPI_ISL_1457605 496 : EPI_ISL_1797747 497 : EPI_ISL_1673301 498 : EPI_ISL_2047723 499 : EPI_ISL_1901578 500 : EPI_ISL_1362485 501 : EPI_ISL_2212992 502 : EPI_ISL_2217349 503 : EPI_ISL_1097295 504 : EPI_ISL_878114 505 : EPI_ISL_1189454 506 : EPI_ISL_1247720

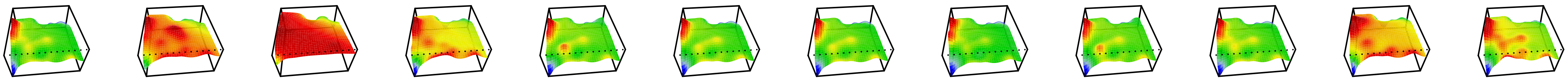

507 : EPI_ISL_997133 508 : EPI_ISL_2478092 509 : EPI_ISL_2139153 510 : EPI_ISL_2423648 511 : EPI_ISL_1540112 512 : EPI_ISL_1551169 513 : EPI_ISL_1576091 514 : EPI_ISL_1754863

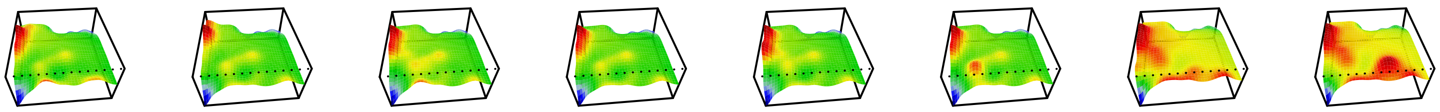

B.1.525_VOC x

515 : EPI_ISL_1715371 516 : EPI_ISL_2460764 517 : EPI_ISL_1893092 518 : EPI_ISL_1755151 519 : EPI_ISL_2351758 520 : EPI_ISL_1821603 521 : EPI_ISL_2454133 522 : EPI_ISL_2116128 523 : EPI_ISL_1972828 524 : EPI_ISL_1820306 525 : EPI_ISL_1112160 526 : EPI_ISL_2465904

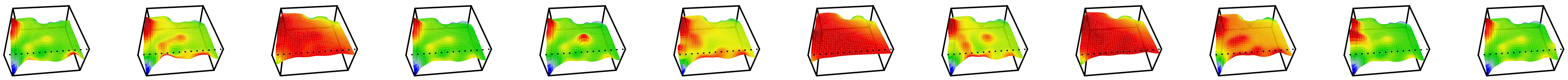

527 : EPI_ISL_2100339 528 : EPI_ISL_2019217 529 : EPI_ISL_2226759 530 : EPI_ISL_1358359 531 : EPI_ISL_1000632 532 : EPI_ISL_2479140 533 : EPI_ISL_2167029 534 : EPI_ISL_2477735 535 : EPI_ISL_2165720 536 : EPI_ISL_2167212 537 : EPI_ISL_2169236 538 : EPI_ISL_1695013

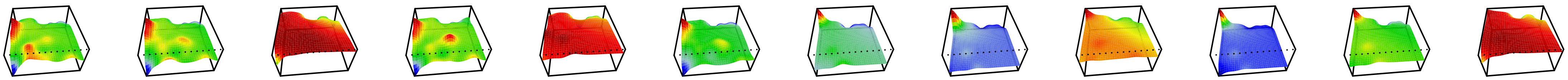

539 : EPI_ISL_1610615 540 : EPI_ISL_2193399 541 : EPI_ISL_1624964

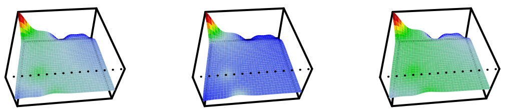

B.1.617.1_VOC x

542 : EPI_ISL_2460542 543 : EPI_ISL_2461482 544 : EPI_ISL_2189661 545 : EPI_ISL_2189660 546 : EPI_ISL_1818619 547 : EPI_ISL_2015634 548 : EPI_ISL_1595867 549 : EPI_ISL_2502035 550 : EPI_ISL_1970528 551 : EPI_ISL_2341964 552 : EPI_ISL_1663458 553 : EPI_ISL_1704454

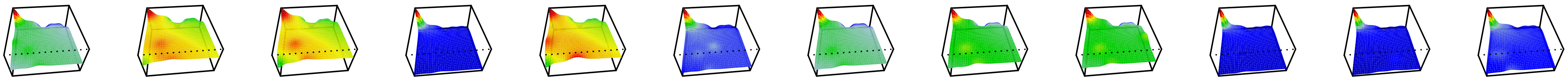

554 : EPI_ISL_1544013 555 : EPI_ISL_2341688 556 : EPI_ISL_1415308 557 : EPI_ISL_2342920 558 : EPI_ISL_2332712 559 : EPI_ISL_2225634 560 : EPI_ISL_1544032 561 : EPI_ISL_2441382 562 : EPI_ISL_2441449 563 : EPI_ISL_1841364 564 : EPI_ISL_2462034 565 : EPI_ISL_2461766

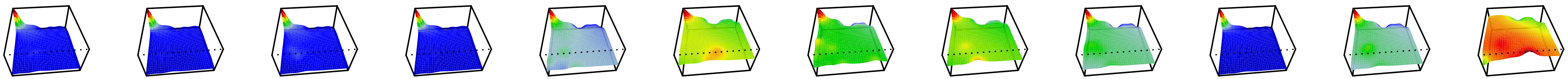

566 : EPI_ISL_2461540 567 : EPI_ISL_2400210 568 : EPI_ISL_2503645 569 : EPI_ISL_2503621 570 : EPI_ISL_2503718 571 : EPI_ISL_2503413 572 : EPI_ISL_1589934 573 : EPI_ISL_2503469 574 : EPI_ISL_2503472 575 : EPI_ISL_2425328 576 : EPI_ISL_2425119 577 : EPI_ISL_1745201

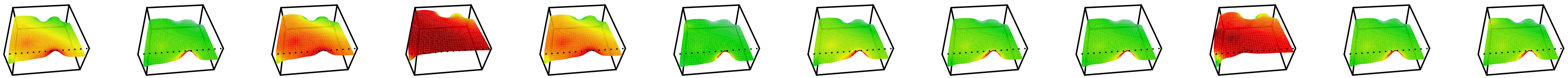

578 : EPI_ISL_2032644 579 : EPI_ISL_1790913 580 : EPI_ISL_1830312 581 : EPI_ISL_1829381 582 : EPI_ISL_1632073 583 : EPI_ISL_2004530 584 : EPI_ISL_1519930 585 : EPI_ISL_1637489 586 : EPI_ISL_2167855 587 : EPI_ISL_2479663 588 : EPI_ISL_1675223 589 : EPI_ISL_1483794

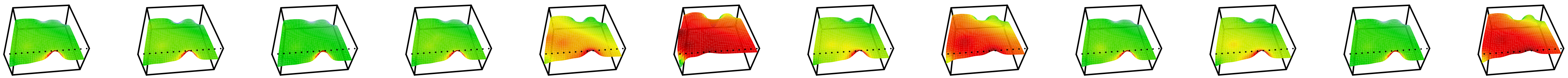

590 : EPI_ISL_1680407

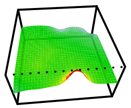

B.1.617.2_VOC x

591 : EPI_ISL_2373233 592 : EPI_ISL_2441211 593 : EPI_ISL_2342881 594 : EPI_ISL_1928601 595 : EPI_ISL_2342303 596 : EPI_ISL_2272862 597 : EPI_ISL_2433605 598 : EPI_ISL_2433607 599 : EPI_ISL_1847907 600 : EPI_ISL_2466790 601 : EPI_ISL_2425699 602 : EPI_ISL_2362103

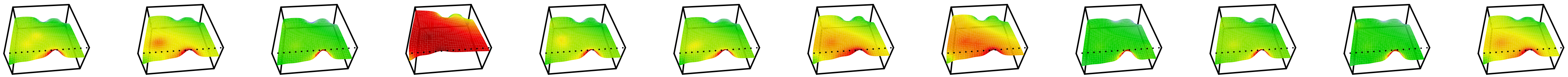

603 : EPI_ISL_1833679 604 : EPI_ISL_2456597 605 : EPI_ISL_2022336 606 : EPI_ISL_2486655 607 : EPI_ISL_2236320 608 : EPI_ISL_2392846 609 : EPI_ISL_2436865 610 : EPI_ISL_2393918 611 : EPI_ISL_2366559 612 : EPI_ISL_2127621 613 : EPI_ISL_2253453 614 : EPI_ISL_2275187

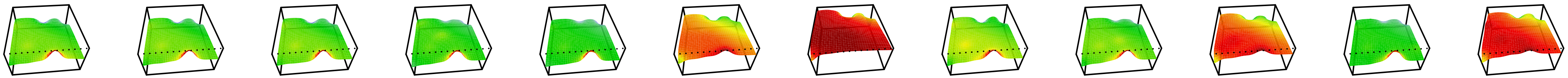

615 : EPI_ISL_1652717 616 : EPI_ISL_2318458 617 : EPI_ISL_2318096 618 : EPI_ISL_1719099 619 : EPI_ISL_2118684 620 : EPI_ISL_2119601 621 : EPI_ISL_2395335 622 : EPI_ISL_2394556 623 : EPI_ISL_2437112 624 : EPI_ISL_2348188 625 : EPI_ISL_2317202 626 : EPI_ISL_2395662

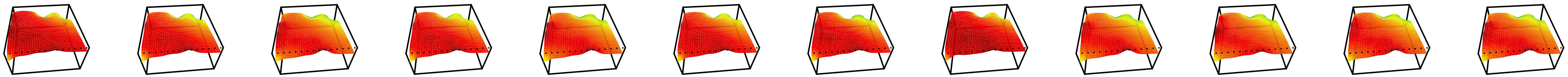

627 : EPI_ISL_2436354 628 : EPI_ISL_2199493 629 : EPI_ISL_2366576 630 : EPI_ISL_2346787 631 : EPI_ISL_2394563 632 : EPI_ISL_1637187 633 : EPI_ISL_2435737 634 : EPI_ISL_2457646 635 : EPI_ISL_2354411 636 : EPI_ISL_2408029 637 : EPI_ISL_2408027 638 : EPI_ISL_2488228

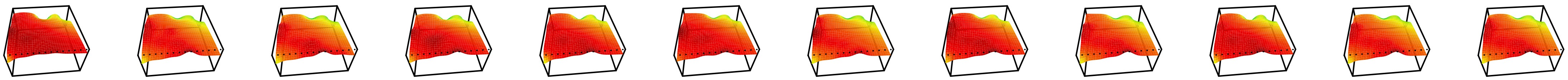

639 : EPI_ISL_2453182 640 : EPI_ISL_2311908

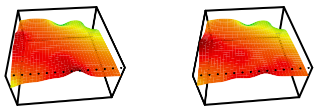

C.37_VOC x

641 : EPI_ISL_2183611 642 : EPI_ISL_1439856 643 : EPI_ISL_2471722 644 : EPI_ISL_2389648 645 : EPI_ISL_2472531 646 : EPI_ISL_2115343 647 : EPI_ISL_2402495 648 : EPI_ISL_2283637 649 : EPI_ISL_1910812 650 : EPI_ISL_2269500 651 : EPI_ISL_2371967 652 : EPI_ISL_1924317

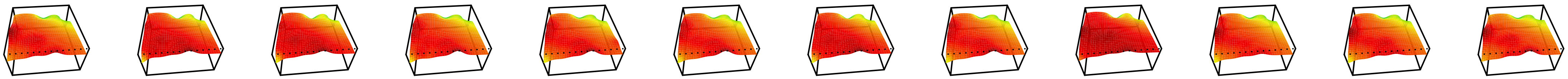

653 : EPI_ISL_2184982 654 : EPI_ISL_2277931 655 : EPI_ISL_1460222 656 : EPI_ISL_2148134 657 : EPI_ISL_2246563 658 : EPI_ISL_2244899 659 : EPI_ISL_1560226 660 : EPI_ISL_2161738 661 : EPI_ISL_1258712 662 : EPI_ISL_1306752 663 : EPI_ISL_1160917 664 : EPI_ISL_2185424

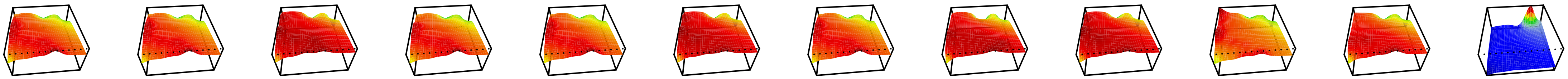

665 : EPI_ISL_1526920 666 : EPI_ISL_2158760 667 : EPI_ISL_2135985 668 : EPI_ISL_2009562 669 : EPI_ISL_2009634 670 : EPI_ISL_2391218 671 : EPI_ISL_2391351 672 : EPI_ISL_1321474 673 : EPI_ISL_2009241 674 : EPI_ISL_2391305 675 : EPI_ISL_2009644 676 : EPI_ISL_2009269

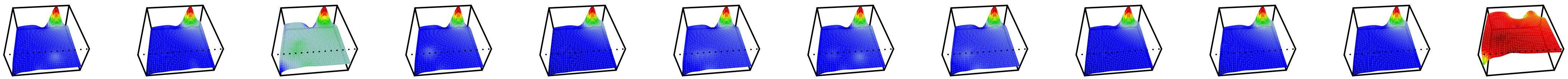

677 : EPI_ISL_2467237 678 : EPI_ISL_2467154 679 : EPI_ISL_2467138 680 : EPI_ISL_2009580 681 : EPI_ISL_1633506 682 : EPI_ISL_2391424 683 : EPI_ISL_2391404 684 : EPI_ISL_1633495 685 : EPI_ISL_2391389 686 : EPI_ISL_2009302 687 : EPI_ISL_2391382 688 : EPI_ISL_1633528

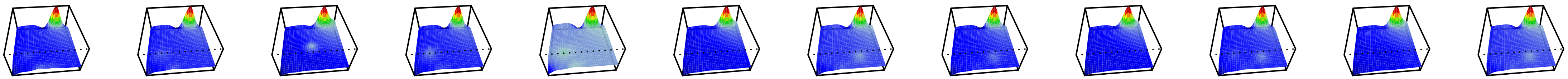

689 : EPI_ISL_2488721 690 : EPI_ISL_2427682

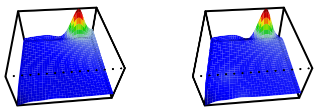

P.3_VOC x

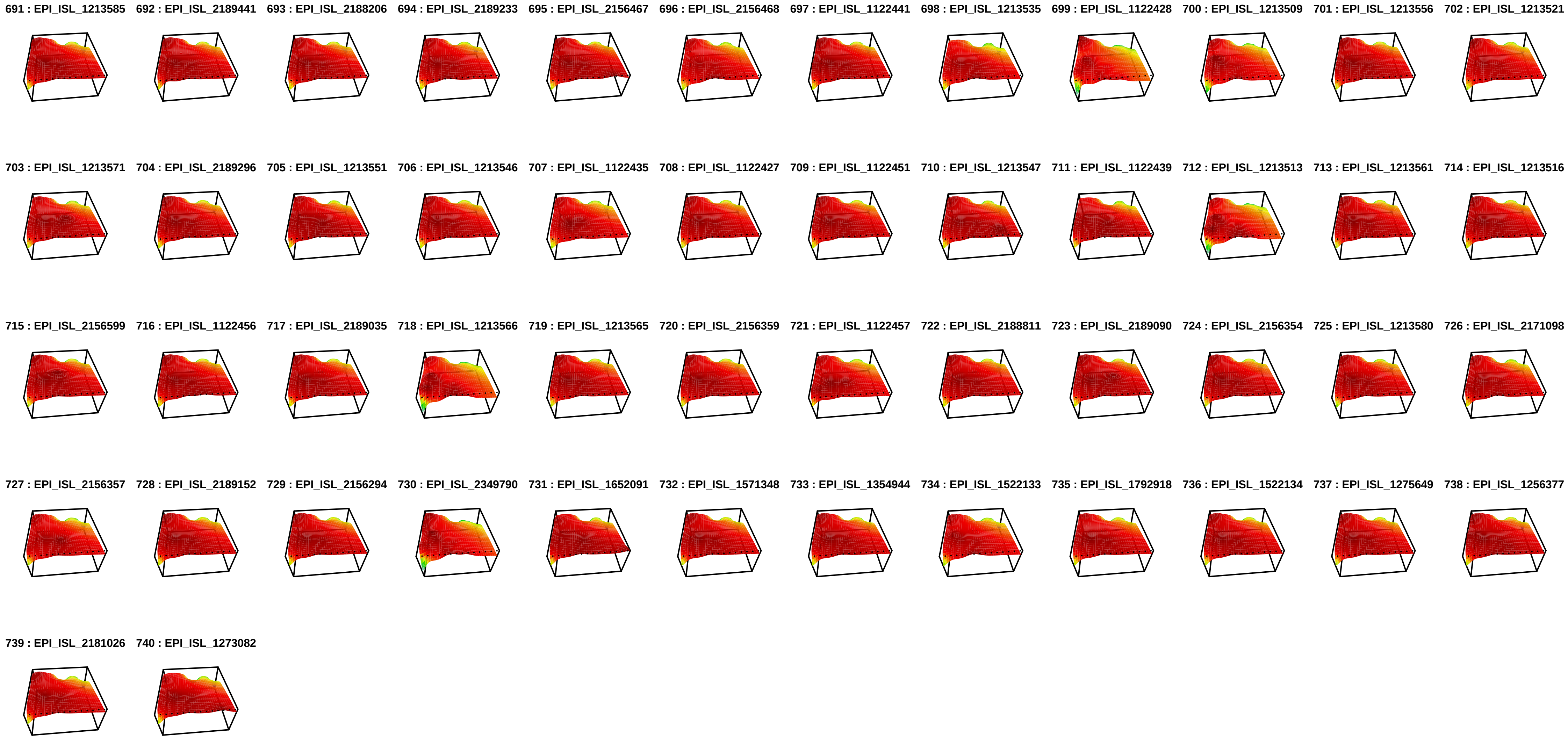
691 : EPI_ISL_1213585
692 : EPI_ISL_2189441
693 : EPI_ISL_2188206
694 : EPI_ISL_2189233
695 : EPI_ISL_2156467
696 : EPI_ISL_2156468
697 : EPI_ISL_1122441
698 : EPI_ISL_1213535
699 : EPI_ISL_1122428
700 : EPI_ISL_1213509
701 : EPI_ISL_1213556
702 : EPI_ISL_1213521
703 : EPI_ISL_1213571
704 : EPI_ISL_2189296
705 : EPI_ISL_1213551
706 : EPI_ISL_1213546
707 : EPI_ISL_1122435
708 : EPI_ISL_1122427
709 : EPI_ISL_1122451
710 : EPI_ISL_1213547
711 : EPI_ISL_1122439
712 : EPI_ISL_1213513
713 : EPI_ISL_1213561
714 : EPI_ISL_1213516
715 : EPI_ISL_2156599
716 : EPI_ISL_1122456
717 : EPI_ISL_2189035
718 : EPI_ISL_1213566
719 : EPI_ISL_1213565
720 : EPI_ISL_2156359
721 : EPI_ISL_1122457
722 : EPI_ISL_2188811
723 : EPI_ISL_2189090
724 : EPI_ISL_2156354
725 : EPI_ISL_1213580
726 : EPI_ISL_2171098
727 : EPI_ISL_2156357
728 : EPI_ISL_2189152
729 : EPI_ISL_2156294
730 : EPI_ISL_2349790
731 : EPI_ISL_1652091
732 : EPI_ISL_1571348
733 : EPI_ISL_1354944
734 : EPI_ISL_1522133
735 : EPI_ISL_1792918
736 : EPI_ISL_1522134
737 : EPI_ISL_1275649
738 : EPI_ISL_1256377
739 : EPI_ISL_2181026
740 : EPI_ISL_1273082
